# Precision prebiotics: Engineering food-derived polysaccharides to target specific SCFA-producing taxa for neuroprotection via the microbiota-gut-brain axis

**DOI:** 10.1016/j.crfs.2026.101471

**Published:** 2026-06-18

**Authors:** Yanmei Yin, Yugang Li, Xiaobin Zhang, Mengwei Jia, Shu Zhu, Chengcheng Liu

**Affiliations:** a Department of Rehabilitation, Shengjing Hospital of China Medical University, No. 16 Puhe Road, Shenyang North New Area, Shenyang, Liaoning Province, 110134, China; b Department of Neurology/Stroke Center, The First Hospital of China Medical University, China; c Liaoning Provincial Key Laboratory of Big Data for Neurological Diseases, China; d Department of Neurosurgery, General Hospital of Northern Theater Command of the People's Liberation Army, Shenyang, China; e Department of Critical Care Medicine, The Fourth Affiliated Hospital of China Medical University, Shenyang, Liaoning Province, 110032, China

**Keywords:** Food polysaccharides, Precision prebiotics, Short-chain fatty acids, Gut microbiota, Gut-brain axis, Neuroprotection

## Abstract

Neurodegenerative and neuropsychiatric disorders lack disease-modifying therapies. The microbiota-gut-brain (MGB) axis, particularly short-chain fatty acid (SCFA)-producing microbiota dysbiosis, has emerged as a conserved driver of neuroinjury pathogenesis. Natural food-derived polysaccharides have been explored as prebiotic substrates, but their clinical translation is hindered by poor target specificity, high interindividual heterogeneity, and low bioavailability. Engineered food-derived polysaccharides, as a next-generation precision prebiotic platform, enable rational tailoring of molecular fine structures via targeted physical, chemical, biological, and combinatorial modification technologies, aiming for strain-specific directional modulation of intestinal SCFA-producing microbiota and multi-pathway neuroprotection through the MGB axis. In this review, we systematically delineate the bidirectional regulatory mechanisms between SCFA-producing microbiota and neural homeostasis, dissect disease-specific pathological cascades driven by SCFA-producing microbiota dysbiosis, and discuss conflicting findings on the dual effects of SCFAs. We further propose a full-chain framework of the structure-activity relationship of engineered polysaccharides, dissecting core modification strategies, strain-specific targeting mechanisms, and a multi-dimensional efficacy evaluation system for these precision prebiotics. Additionally, we assess safety evaluation status, major global regulatory differences, and core clinical translation bottlenecks. Finally, we outline key unresolved challenges and propose a conceptual roadmap for AI-assisted rational design of precision prebiotics, personalized microbiota-adapted intervention strategies, and multicenter clinical translation directions. This review provides a mechanism-driven theoretical framework and practical guidance for developing engineered food-derived polysaccharides as precision nutrition interventions for neuroinjury-related disorders.

## Highlights

• Two molecular screening barriers establish predictable structure-activity rules for polysaccharide-microbiota targeting.
• Customized polysaccharides for AD, PD, MDD, and PSCI specify optimal intervention windows and structural parameters.
• Synthesis, delivery, safety, and bacterial enrichment are integrated into a precise polysaccharide modification system.

## Graphical abstract

**Figure:**
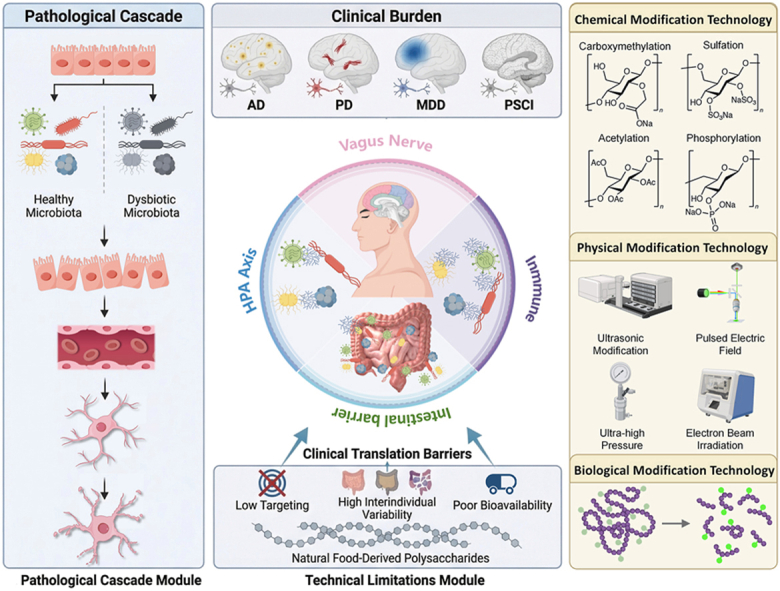

## 1 Introduction

In recent years, central nervous system (CNS) disorders have become a major global public health challenge. According to the latest data from the Global Burden of Disease (GBD) study, the number of people affected by these disorders has exceeded 150 million, with an annual growth rate of 4.2%. They are one of the leading causes of disability and premature death among the global adult population, placing a heavy burden on healthcare systems and socio-economic development worldwide (Paul and Natarajan, 2025). These diseases are generally characterized by complex pathogenesis, disease progression, marked clinical phenotypic heterogeneity, and insidious early onset. Current interventions primarily focus on symptomatic relief, and treatment outcomes vary significantly among individuals. Traditional intervention models struggle to achieve personalized and precise treatment, and the lack of targeted therapeutic strategies poses significant challenges for disease prevention and control (Guo et al., 2025a).

**Fig. 1:**
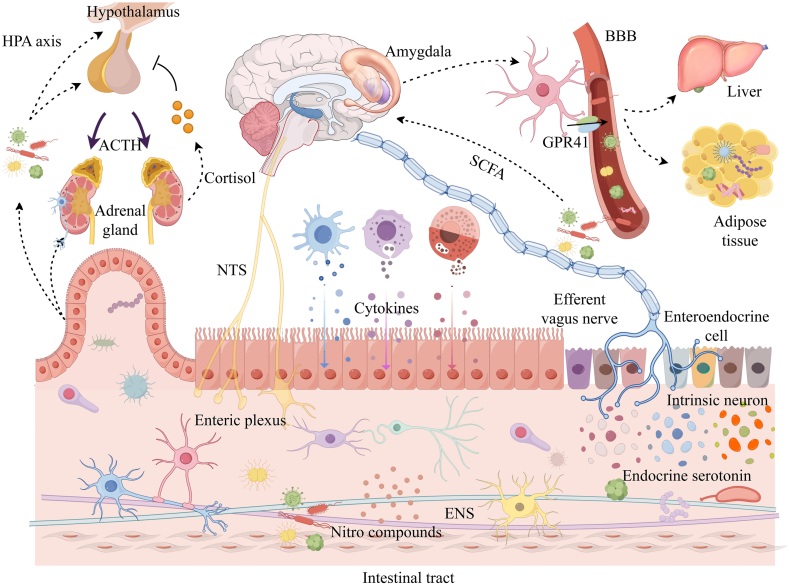
Schematic diagram of the bidirectional regulatory network of the MGB Axis This figure illustrates the bidirectional signaling network of the MGB axis, encompassing the hypothalamic-pituitary-adrenal (HPA) axis, vagus nerve, enteric nervous system (ENS), and short-chain fatty acids (SCFAs) as core metabolites. SCFAs traverse the blood-brain barrier (BBB) to activate GPR41, while cytokines, serotonin, and cortisol mediate endocrine and inflammatory crosstalk between the intestinal tract, brain, liver, and adipose tissue, underlying the pathological and regulatory mechanisms of central nervous system (CNS) diseases.

In response to this clinical challenge, in-depth exploration of MGB axis has opened up new avenues of research and intervention targets for the prevention and treatment of CNS diseases. Existing studies have confirmed that the homeostasis of the gut microbiota plays a crucial regulatory role in brain physiology and neurological health. The MGB axis, along with its core metabolites, short-chain fatty acids (SCFAs), plays a pivotal role in the pathological progression and targeted intervention of CNS diseases, serving as the core communication network for bidirectional signal transduction between the intestinal microecosystem and the CNS (Fig. 1). Numerous population-based cohort studies have now widely observed that dysbiosis of SCFA-producing bacteria, associated with an imbalance in the MGB axis, is significantly correlated with the onset and progression of various CNS diseases. SCFAs are the primary terminal metabolites of dietary fiber through anaerobic fermentation by gut microbiota, with acetic acid, propionic acid, and butyric acid as the main functional components (Yang et al., 2025). As important messenger molecules in the MGB axis, SCFAs mediate bidirectional gut-brain regulation through multiple synergistic pathways, making strategies targeting SCFA-producing microbiota a key breakthrough in neuroprotection and CNS disease intervention research. Food-derived polysaccharides, with the inherent advantages of wide natural sources, excellent biocompatibility, and low toxicity, are widely recognized as promising prebiotic substrates for regulating intestinal SCFA-producing microbiota. These macromolecular carbohydrates, abundant in edible plants, macrofungi, and algae, can selectively promote the proliferation of beneficial gut bacteria including *Bifidobacterium*, *Lactobacillus*, and *Roseburia*, enhance intestinal SCFA synthesis, and exert indirect neuroprotective effects via the MGB axis (Cantu-Jungles et al., 2024), confirming their application potential in neuroprotection through the MGB axis.

However, natural food-derived polysaccharides face multiple bottlenecks in practical application, including poor water solubility, high structural heterogeneity, low bioavailability, and inconsistent inter-individual responses, which substantially hinder their precise and efficient regulation of target SCFA-producing microbiota (Chen et al., 2025). To address these limitations, a comprehensive technical system for engineered polysaccharide modification has been developed in the fields of food science and precision nutrition. Researchers can precisely regulate the molecular weight, monosaccharide composition, glycosidic bond configuration, branched chain topology, and surface functional groups of polysaccharides through physical, chemical, or enzymatic modification methods. Such engineered modification can endows polysaccharides with improved dissolution stability, higher targeting selectivity for gut microbiota, and more consistent host metabolic response (Zhao et al., 2025a). These engineered dietary fibers address the inherent defects of natural polysaccharides, enabling customized nutritional interventions based on an individual's baseline gut microbiota, disease type, and progression stage (Deisenhofer et al., 2024). Despite broad theoretical prospects, key scientific and technical bottlenecks remain in this field. Most existing studies focus on verifying the phenotypic effects of natural polysaccharides, with no systematic analysis of the structure-activity relationship and molecular mechanism of engineered modification and neuroprotective effects, and often fail to distinguish phenotypic correlation from causal effects. Meanwhile, the lack of mature intervention strategies targeting individual microbiota heterogeneity further complicates the clinical translation and application expansion of engineered polysaccharides (Wang et al., 2025a; Fusco et al., 2023).

At the same time, much of the current literature on prebiotics, the MGB axis, and SCFAs tends to focuses on the prebiotic effects of natural polysaccharides, the physiological functions of SCFAs, or the mechanisms of action of the MGB axis in individual CNS disorders. Furthermore, most relevant reviews discuss neuropathological mechanisms and polysaccharide engineering techniques separately, and only provide scattered introductions to modification methods for engineered polysaccharides. These contents have not been integrated into a systematic framework; there has been no systematic analysis of the specific characteristics of SCFA-producing microbial dysbiosis in different CNS disorders, nor is there clarity regarding the differences in targeted intervention sites for various diseases. There is an clear need to establish a systematic theoretical framework at the intersection of food science and neuroscience.

In response to the gaps in the aforementioned field and the systematic limitations of existing reviews, this paper proposes novel concept of precision prebiotics. Building on the foundation of neurodisease subtype classification and pathological target analysis, this approach aims to involve the specialized, precise customization and optimization of functional polysaccharide molecular structures to achieve strain-level, specific enrichment of the gut microbiota. This, in turn, regulates the differential production and homeostatic balance of various SCFA subtypes, ultimately enabling targeted, precise intervention in pathological pathways associated with neurological damage. Furthermore, this paper examines the mechanism by which engineered food-derived polysaccharides protect neurons through the microbiota-gut-brain axis by regulating SCFA-producing bacteria. It reviews the latest research advances at the intersection of food science, gut microbiology, and neuroscience; analyzes the process by which engineered polysaccharides regulate gut microbiota metabolism and neural function; evaluates the advantages and limitations of existing modification technologies; and summarizes a multidimensional technical framework for assessing prebiotic efficacy. Meanwhile, this review analyzes the safety evaluation status and clinical translation bottlenecks of engineered food-derived polysaccharides, and proposes key future research directions. Ultimately, this work seeks to provide a theoretical basis and practical guidance for the clinical translation of engineered food-derived polysaccharides as precision nutrition intervention tools for CNS diseases. This article is presented as a mechanistic review, which defines the research scope, summarizes relevant mechanisms, and outlines the research objectives in this interdisciplinary field.

### 1.1 Eligibility criteria

#### 1.1.1 Inclusion criteria

Literature theme: Studies focusing on food-derived polysaccharides that simultaneously address the regulatory relationships among “structural/engineered modification”, “SCFAs-producing microbiota”, and the “microbiota–gut–brain axis”.

Keyword search strategy:

Primary keywords: food-derived polysaccharides, structural modification, SCFAs-producing microbiota, microbiota–gut–brain axis, neuroprotection.

Secondary qualification terms: engineered polysaccharides, precision modulation of gut flora, SCFA-mediated neural signaling, MGB axis intervention – to ensure that the included literature focuses on the precise regulation of SCFA-producing microbiota by engineered polysaccharides and the associated neuroprotective mechanisms.

Time frame: Priority is given to peer-reviewed studies published between 2020 and 2026, covering basic mechanisms, animal models, and preclinical evidence.

Study types: Mechanistic studies, in vitro/in vivo experiments, integrative analyses of microbiota-metabolome-neurobehavioral correlations, and functional validation studies based on the microbiota-gut-brain axis.

#### 1.1.2 Exclusion criteria

Studies not involving food-derived polysaccharides.

Prebiotic neuroeffect studies that do not use SCFAs as core mediator molecules.

Studies that only describe the basic phenotype of native polysaccharides without performing any form of engineered structural modification.

Literature that lacks mechanistic elaboration of the gut microbiota–brain axis pathway or focuses exclusively on local intestinal effects without addressing the central nervous system.

## 2 The microbiota-gut-brain axis: bidirectional synergistic regulation between SCFA-producing microbiota and neural homeostasis

**Fig. 2:**
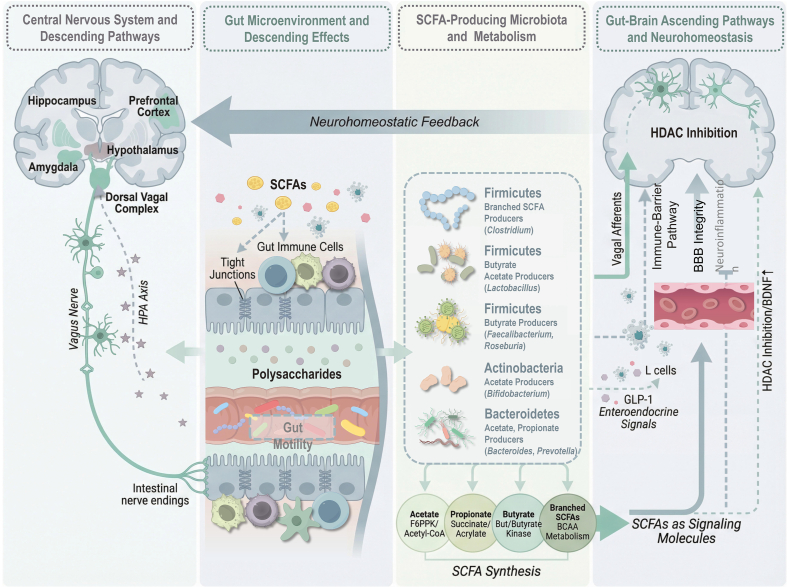
Schematic of Polysaccharide-Modulated SCFA-Producing Microbiota and Neurohomeostasis via the Gut-Brain Axis This figure depicts the bidirectional gut-brain axis network, where polysaccharides shape the gut microenvironment and enrich SCFA-producing microbiota (Firmicutes, Actinobacteria, Bacteroidetes) to synthesize SCFAs. As signaling molecules, SCFAs activate vagal afferents, strengthen blood-brain barrier integrity, inhibit HDAC and neuroinflammation, and regulate neurohomeostasis via the HPA axis and BDNF, while neural feedback modulates gut motility and immune function.

The MGB axis represents a key bidirectional communication network linking intestinal microecology to CNS function. Short-chain fatty acid (SCFA)-producing microbiota and their metabolites are important mediators of this crosstalk. The CNS may indirectly modulate the colonization and metabolic activity of SCFA-producing microbiota via the hypothalamic-pituitary-adrenal (HPA) axis, autonomic nervous system, and intestinal microenvironment. Conversely, acetate, propionate, and butyrate can affect the CNS through immune regulation, vagal nerve transmission, endocrine signaling, and epigenetic modifications, thereby contributing to neuroinflammation, neuroplasticity, and stress homeostasis (Fig. 2). Current research has primarily focused on the gut-to-brain ascending pathway, whereas reciprocal feedback loops remain underexplored. Most evidence derives from rodent models, and human cohort data show notable variability. Moreover, unresolved questions, including the potential neurotoxicity of high SCFA concentrations and mechanisms underlying CNS lesion-induced gut microbiota suppression—hinder the clinical translation of microbiota-targeted interventions. A detailed understanding of the taxonomic and metabolic features of SCFA-producing microbiota is therefore necessary to elucidate the regulatory mechanisms of the MGB axis.

### 2.1 Taxonomic affiliation of gut SCFA-producing microbiota and the molecular metabolic basis of SCFA biosynthesis

SCFA-producing microbiota ferment carbohydrates such as dietary fiber that are indigestible by the host, with SCFAs as their primary terminal metabolites. These microbiota display clear taxonomic affiliation, substrate utilization preferences and metabolic pathway specificity, and act as functional communities mediating signal transmission along MGB axis (Butyrate: a key mediator of). Highly efficient SCFA-producing bacteria in the human gut exhibit distinct taxonomic features and functional differentiation, and mainly include members of three phyla, namely Firmicutes, Bacteroidetes and Actinobacteria. These phyla collectively form the functional framework for SCFA biosynthesis in the intestine. Among them, Firmicutes serves as the core functional microbiota for butyrate production in the gut. Representative genera include *Faecalibacterium* and *Roseburia* belonging to *Lachnospiraceae*, which contribute most of the butyrate in the human gut through dedicated metabolic pathways (Pu et al., 2024). *Ruminococcus* from *Ruminococcaceae* acts as a secondary butyrate-producing genus. Within *Bacteroidetes*, *Bacteroides* and *Prevotella* dominate the biosynthesis of acetate and propionate. These bacteria possess a strong polysaccharide-degrading capacity encoded by carbohydrate-active enzymes (CAZymes) genes, which greatly promotes SCFA production (Vavricka et al., 2025). *Actinobacteria* is represented by *Bifidobacterium*, a genus specialized for acetate synthesis and a key probiotic group in the infant gut (Mukhopadhya and Louis, 2025). The taxonomic classification and synthetic functions of each phylum are highly specific.

**Fig. 3:**
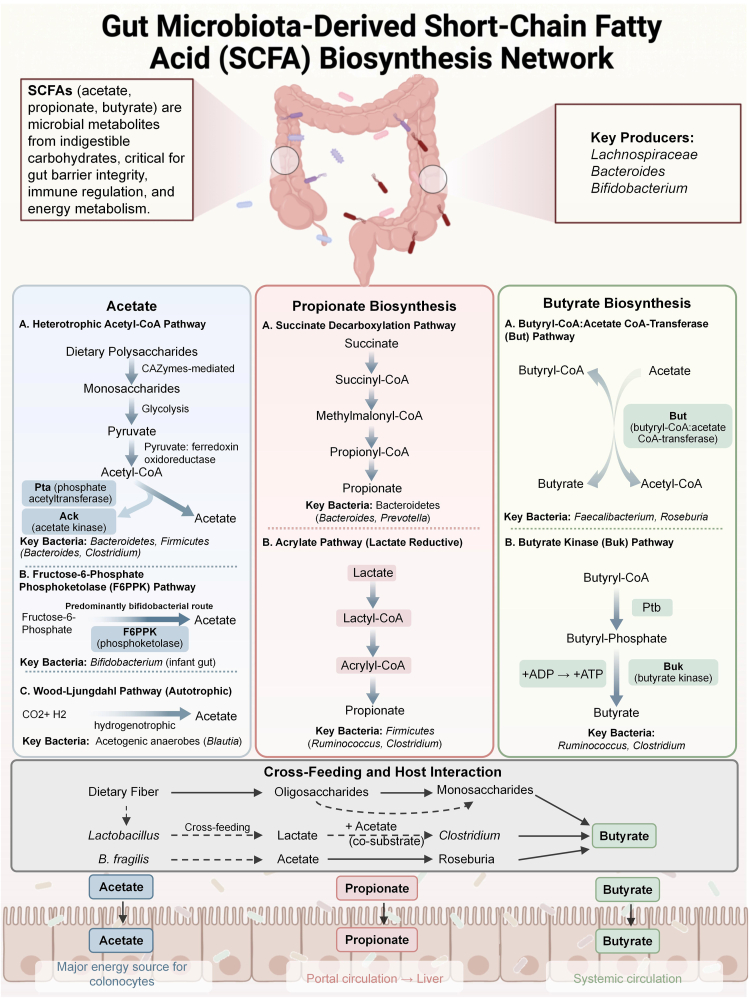
Synthesis of Gut Microbiota-Derived Short-Chain Fatty Acids This figure illustrates the main pathways for acetate, propionate, and butyrate synthesis by gut microbiota, highlights key producing bacteria and cross-feeding processes, and depicts their metabolic fates and physiological functions in the host.

Driven by differences in taxonomic features, each group of SCFA-producing microbiota has exclusive metabolic synthetic pathways, terminal metabolite types and substrate utilization preferences. Highly specific correspondences exist between different genera and these attributes. The core pathways for acetate synthesis include the acetyl-coenzyme A pathway and the fructose-6-phosphate phosphoketolase pathway unique to *Bifidobacterium* (He et al., 2023). Propionate synthesis is dominated by the succinate decarboxylation pathway driven by *Bacteroidetes*, and is supplemented by the acrylate pathway mediated by *Firmicutes* (Reichardt et al., 2014; Louis and Flint, 2017). Butyrate synthesis mainly relies on the butyryl-coenzyme A, acetate-coenzyme A transferase pathway, which is exclusive to *Firmicutes*. The butyrate kinase pathway is a minor supplementary route (Li et al., 2012; Lee et al., 2024a). Different genera show remarkable differences in polysaccharide substrate preferences, which provide a scientific basis for strain-specific enrichment and precise regulation of SCFA profiles via engineered modification of food-derived polysaccharides. The taxonomic affiliation, synthetic pathways, key enzyme systems and substrate-product correlations of these microbiota are detailed in Fig. 3.

Of particular importance, SCFAs in the gut exist mainly as a mixture of acetate, propionate and butyrate. The three metabolites show both synergistic metabolism and functional antagonism, and their dynamic balance is a key factor determining the final physiological effects of SCFAs. From a metabolic perspective, acetate produced by *Bacteroidetes* greatly improves butyrate synthesis efficiency through metabolic cross-feeding among gut microbiota, forming a collaborative metabolic model from acetate production to butyrate conversion. In terms of functional activity, the three SCFAs competitively bind to free fatty acid receptors such as GPR41/43. Divergent binding affinities of different receptor subtypes for fatty acids change the activation status of downstream signaling pathways, leading to functional mutual antagonism (Lee et al., 2024b). The dynamic balance formed by synergistic and antagonistic interactions among SCFAs not only determines their final physiological effects, but also explains the inherent mechanism for the distinct differences between supplementation with a single SCFA and global SCFA level regulation via microbiota modulation.

### 2.2 Core pathological pathways mediating neurological injury induced by SCFA-producing microbiota dysbiosis

SCFA-producing microbiota regulate neural homeostasis mainly through their metabolic end products, SCFAs. SCFAs are capable of modulating central nervous system (CNS) functions via multiple physiological routes of the MGB axis (Chib et al., 2024). Metabolic pathway variations among different SCFA-producing strains determine the relative proportions of acetate, propionate, butyrate and other SCFAs, and further affect the scope and intensity of their regulatory effects.

Immunoinflammatory regulation serves as the core pathway underlying the neuroprotective effects of SCFAs. Microbiota dysbiosis is accompanied by reduced butyrate synthesis, which may abolish the endogenous inhibitory effect on the nuclear factor kappa B (NF-κB) signaling pathway. As a master switch for pro-inflammatory transcription, NF-κB remains persistently activated in the absence of butyrate modulation. This persistent activation stimulates the synchronous secretion of numerous pro-inflammatory cytokines in both peripheral and central systems, such as TNF-α, IL-1β and IL-6, thereby triggering an inflammatory cascade effect (Abreu et al., 2025). Recent studies have demonstrated that Th1/Th17-type cytokines such as IFN-γ and IL-17A not only participate in peripheral immune activation but also rapidly disrupt the tight junction structure of the blood-brain barrier (BBB) and intestinal epithelial barrier within 1 h. These cytokines induce the redistribution of ZO-1, occludin and claudin-5 proteins and synergistically amplify the invasion of neuroinflammatory factors (Zheng and Luo, 2025). At the central level, microglia undergo phenotypic polarization under sustained exposure to high concentrations of inflammatory mediators. Microglia transform from homeostatic M2 phenotype to pro-inflammatory M1 phenotype, release reactive oxygen species and proteases, damage synaptic structures and induce neuronal apoptosis (Sun et al., 2025). Astrocytes are synchronously activated to form glial scars that hinder axonal regeneration. All these pathological changes collectively lead to irreversible neurological injury and cognitive decline. Nevertheless, the anti-inflammatory effects of butyrate exhibit strict concentration and brain region specificity. Existing studies predominantly focus on the protective properties of butyrate, while the effective concentration window and safety threshold of butyrate under pathological conditions remain poorly defined. In addition, butyrate modulates neuroinflammation through dual microbiota-dependent and microbiota-independent routes. Multiple germ-free animal experiments have verified that the central neuroprotective effects of butyrate are markedly attenuated under germ-free conditions (Roseburia hominis Alleviates Neuroinflammation via; Lahariya et al., 2026a; Lahariya et al., 2026b). This finding indicates that the major functions of butyrate rely on secondary metabolic regulation of intestinal microbiota rather than direct central actions, which also explains the significant discrepancies between conclusions derived from in vitro cell experiments and in vivo animal studies.

Human disease cohort studies have only confirmed that butyrate deficiency caused by SCFA-producing microbiota dysbiosis is significantly correlated with structural and functional damage to the intestinal epithelial barrier and blood-brain barrier (Mishra et al., 2025). At the intestinal level, butyrate acts as the primary energy substrate for colonic epithelial cells. Butyrate deficiency impairs mitochondrial ATP synthesis, reduces the mRNA stability of tight junction proteins including occludin and claudin family members, and decreases their protein expression levels by 30% to 50%. The loss of epithelial barrier integrity results in intestinal leakage, which allows massive entry of endotoxin (LPS), bacterial DNA fragments and pro-inflammatory cytokines such as IL-17A and IFN-γ into the bloodstream (Beltran-Velasco and Clemente-Suárez, 2025). These substances reach cerebral microvascular endothelial cells through the circulatory system. They bind to EGFR/PAR2 receptors or activate the STAT3 pathway, induce the dissociation of claudin-5 and ZO-1 from paracellular gaps within 1 to 3 h, and trigger the collapse of F-actin cytoskeleton. The permeability of the blood-brain barrier accordingly increases by 2 to 3 folds and permits free infiltration of peripheral inflammatory mediators into the brain parenchyma (Arteaga-Blanco et al., 2026). Animal model studies have shown that intestinal barrier damage induced by chronic stress can elevate cerebral TNF-α levels by 4.7 times, reduce the dendritic spine density of neurons in the hippocampal CA1 region and cause the loss of synaptic protein PSD-95. These pathological alterations eventually manifest as learning and memory impairment (Lapmanee et al., 2024). Longitudinal cohort investigations on Alzheimer's disease (AD), Parkinson's disease (PD) and other neurological diseases have revealed that abnormal intestinal permeability cannot be detected in some patients before the onset of clinical symptoms. This evidence suggests that increased intestinal permeability acts more as an amplifying factor for disease progression rather than a universal initiating factor for all neurological disorders. Current research overgeneralizes the universal applicability of dual barrier damage across different diseases and disease stages (Davoody et al., 2025). Based on available evidence, this study regards dual barrier damage as a crucial pathway for neurological injury mediated by SCFA-producing microbiota dysbiosis instead of a common initiating factor for all neurological diseases.

Reduced contents of acetate and butyrate resulting from SCFA-producing microbiota imbalance impair the functions of intestinal endocrine L cells. L cells are mainly distributed in the distal ileum and colon, and the GPR41/FFAR3 receptors expressed on their surface function as sensors for SCFAs. Insufficient SCFA concentrations reduce the secretion of GLP-1 and PYY in L cells by 40% to 60%. On one hand, this reduction weakens the activation effect on POMC neurons in the hypothalamic arcuate nucleus and further causes appetite dysregulation and metabolic syndrome. On the other hand, decreased circulating GLP-1 levels relieve its inhibitory effect on CRH neurons in the hypothalamic paraventricular nucleus (PVN). This process leads to persistent hyperactivity of the hypothalamic-pituitary-adrenal (HPA) axis and elevates plasma cortisol concentrations by 2 to 3 folds (Liu et al., 2025a; Panjwani et al., 2025). Animal model experiments have validated that chronic high cortisol status damages hippocampal dentate gyrus neurogenesis by inhibiting the BDNF/TrkB signaling pathway, reduces the number of newborn neurons, and induces neuronal dendritic atrophy and synaptic plasticity dysfunction. Meanwhile, excessive cortisol activates the glutamatergic system in the prefrontal cortex and causes the accumulation of excitotoxicity together with relative insufficiency of GABAergic inhibitory function. These changes ultimately lead to anxiety-like and depression-like behaviors as well as working memory deficits (Krasner et al., 2025). Such phenotypic changes can be reversed by intervention with GLP-1 receptor agonists, which preliminarily confirms the regulatory mechanism of this pathway. However, conclusions differ between the vagus nerve-mediated mode and the direct blood circulation action mode. The contribution ratio and applicable conditions of the two modes remain unclear.

The epigenetic regulatory pathway serves as the final link that drives the progression of neurological injury induced by SCFA-producing microbiota dysbiosis from reversible damage to irreversible degeneration. In cellular experiments, butyrate functions as a histone deacetylase (HDAC) inhibitor (Rahmani et al., 2026). Whether physiological concentrations of butyrate in vivo can reach the effective functional concentration in the central nervous system remains controversial. In in vitro neuronal culture systems, butyrate inhibits the activity of class I/II HDAC enzymes, increases the acetylation level of histone H3K9/K14 in hippocampal and prefrontal cortical neurons, and remodels chromatin structure to an open state. These changes facilitate CREB binding to the BDNF exon IV promoter region and further promote the transcription of BDNF gene. Butyrate deficiency induced by SCFA-producing microbiota dysbiosis results in uncontrolled HDAC activity, which causes histone hypoacetylation and CpG island hypermethylation in the BDNF promoter region and ultimately leads to gene silencing. Animal model experiments have confirmed the above pathological process. SCFA microbiota dysbiosis markedly downregulates the protein expression of hippocampal BDNF and suppresses neuronal synaptic plasticity and neurogenesis. Exogenous butyrate supplementation can reverse such pathological changes and preliminarily verify the correlation between butyrate deficiency and BDNF dysregulation (Duan et al., 2025). In addition, methyl-CpG binding protein 2 (MeCP2) recruits the HDAC/Sin3A complex to further strengthen the transcriptional inhibition of BDNF. This regulatory mechanism has been validated in animal models of Rett syndrome (Lorincz et al., 2001). The reduction of SCFAs caused by microbiota dysbiosis firstly induces intestinal barrier damage and peripheral immune disorder to complete the peripheral initiation of pathological processes. It then triggers central neuroinflammation through the damaged blood-brain barrier and amplifies disease signals in the central nervous system. Epigenetic modification finally consolidates neuronal injury and promotes the progression of diseases from reversible acute inflammation to irreversible chronic degeneration. Although this pathological model has been clarified, the physiological feasibility of central epigenetic regulation mediated by butyrate is still questionable (Stilling et al., 2016). Existing studies mostly confirm the HDAC inhibitory effect of butyrate based on in vitro assays. Peripheral butyrate hardly penetrates the intact blood-brain barrier to achieve effective inhibitory concentrations under physiological conditions. The cerebrospinal fluid concentration of butyrate after oral administration in humans is only 1/50 to 1/20 of that in peripheral blood. This indicates that the in vivo effects of butyrate may derive indirectly from peripheral inflammation inhibition, and obvious inconsistencies exist between in vitro and in vivo functional outcomes.

The four major pathological pathways do not function independently. They form intercrossed and positive feedback cascaded damaging effects with close mutual connections. The crosstalk among these pathways acts as a critical driving factor for the progression of neurological injury from reversible acute damage to irreversible chronic degeneration. Bidirectional malignant crosstalk exists between the immunoinflammatory pathway and barrier integrity pathway. Pro-inflammatory cytokines directly destroy the tight junction structure of intestinal epithelial and blood-brain barriers. The endotoxin translocation caused by impaired barrier integrity further amplifies systemic and central inflammatory responses and forms a vicious cycle of inflammation aggravation triggered by barrier damage. The neuroendocrine pathway is deeply interconnected with the above two pathways. Cortisol released by persistent HPA axis hyperactivity exacerbates intestinal barrier damage and neuroinflammatory activation. Peripheral and central inflammatory signals in turn weaken the negative feedback regulatory capacity of the HPA axis and aggravate endocrine homeostatic imbalance. As the terminal link for the consolidation of neurological injury, the epigenetic regulatory pathway is coordinately modulated by the three upstream pathways. Chronic inflammation, barrier damage and endocrine disorder collectively drive the epigenetic silencing of neuroprotective genes such as BDNF. Epigenetic abnormalities conversely aggravate pathological damage of upstream pathways and eventually result in persistent neurological impairment.

### 2.3 Reciprocal regulation of SCFA-producing microbiota by the CNS through descending brain-gut pathways

The MGB axis functions as a dynamic bidirectional regulatory system. The central nervous system (CNS) actively shapes the intestinal microenvironment through multiple routes and exerts reciprocal modulation on the abundance and physiological activity of SCFA-producing microbiota. Functional disruption of this descending brain-gut regulatory pathway acts as one of the major contributors to the progressive deterioration of SCFA-producing microbiota dysbiosis and unsatisfactory intervention outcomes during the progression of neurological diseases. The CNS achieves reciprocal regulation on SCFA-producing microbiota mainly via three primary pathways, and such regulatory patterns present clear disease-specific profiles across different neurological disorders.

The central nervous system directly modulates intestinal peristalsis and barrier function through the autonomic nervous pathway. Psychological stress and central neuropathological abnormalities can activate the sympathetic nervous system and suppress the activity of parasympathetic vagus nerve. These physiological changes downregulate the expression of intestinal epithelial tight junction proteins, increase intestinal permeability and trigger intestinal leakage, while altering the rate of intestinal peristalsis simultaneously. Such pathological conditions not only directly inhibit oxygen-sensitive butyrate-producing symbiotic bacteria such as *Faecalibacterium* and *Roseburia*, but also create ecological niches for the proliferation of pathogenic bacteria including *Escherichia coli* and *Clostridium*. These alterations ultimately lead to a reduction in the total production of SCFAs. Oxygen-sensitive butyrate-producing bacteria are the most susceptible population affected by this pathological process. This regulatory pathway exhibits unique specific characteristics in different disease contexts. Degeneration of the central cholinergic system in AD patients impairs the efferent function of the vagus nerve, slows intestinal peristalsis and prolongs the retention time of metabolic substrates. These changes further disrupt the metabolic rhythm of SCFA-producing microbiota (Bertin et al., 2025). Pathological deposition of α-synuclein within the enteric nervous system of PD patients disturbs the autonomic nervous regulation of the intestine through descending pathways. This process induces early intestinal dysmotility and decreased abundance of butyrate-producing bacteria, which serves as a core driving factor for microbiota dysbiosis in the prodromal stage of PD (Pang et al., 2026). Excessive activation of the sympathetic nervous system in patients with major depressive disorder (MDD) induces intestinal barrier damage and intestinal motility disorder, which represents a critical negative driving factor for the declined abundance of butyrate-producing bacteria in MDD patients (Ioannou et al., 2025). Patients with post-stroke cognitive impairment (PSCI) suffer from central ischemic injury and neural circuit destruction caused by stroke. The descending pathway disrupts autonomic nervous balance, impairs the structural integrity of the intestinal barrier and disturbs peristalsis rhythm, and ultimately results in the reduction of butyrate-producing microbiota abundance (Ren et al., 2026).

The neuroendocrine pathway also participates in the remote regulation of the intestinal immune microenvironment and further affects the homeostasis of SCFA-producing microbiota. Persistent hyperactivity of the hypothalamic-pituitary-adrenal axis triggered by central pathological abnormalities elevates circulating cortisol concentrations abnormally. This condition changes the secretory profile of intestinal immune cells and remodels the local intestinal immune microenvironment consequently. Under physiological conditions, descending central signals promote dendritic cells, Treg cells and macrophages to secrete anti-inflammatory factors such as IL-10 and IL-4, and establish an immune microenvironment suitable for the colonization and growth of SCFA-producing symbiotic bacteria (Zhao et al., 2025b). The sustained pro-inflammatory state induced by chronic stress or central pathological lesions preferentially eliminates inflammation-sensitive butyrate-producing bacteria and breaks the structural balance of intestinal microbiota. This regulatory characteristic is most prominent in patients with major depressive disorder. Continuous high cortisol levels caused by HPA axis hyperactivity serve as a reverse driving factor for the reduction of butyrate-producing bacteria in MDD patients. It also accounts for the unsatisfactory efficacy of single butyrate supplementation in some clinical cases (Li et al., 2025a). The CNS regulates substrate supply for SCFA-producing microbiota by modulating host feeding behavior. Brain-dominated dietary selection directly determines the category and total quantity of metabolic substrates available for intestinal microbiota. High-fiber diets markedly improve the abundance and metabolic activity of SCFA-producing bacteria (Opperman et al., 2026). In contrast, high-fat and high-sugar diets induce obvious microbiota dysbiosis. Such dysbiosis is manifested as an altered ratio of Firmicutes to Bacteroidetes, a sharp decline in total butyrate production, and a significant reduction in the abundance of butyrate-producing bacteria (Nagano et al., 2025). Patients with neurological diseases often suffer from abnormal appetite and monotonous dietary structure. These problems lead to inadequate substrate provision for SCFA-producing microbiota, further aggravate microbiota dysbiosis, and eventually form a self-circulating pathological vicious cycle.

## 3 Pathological mechanisms of neuroinjury mediated by dysbiosis of SCFA-producing microbiota via the microbiota-gut-brain axis

**Fig. 4:**
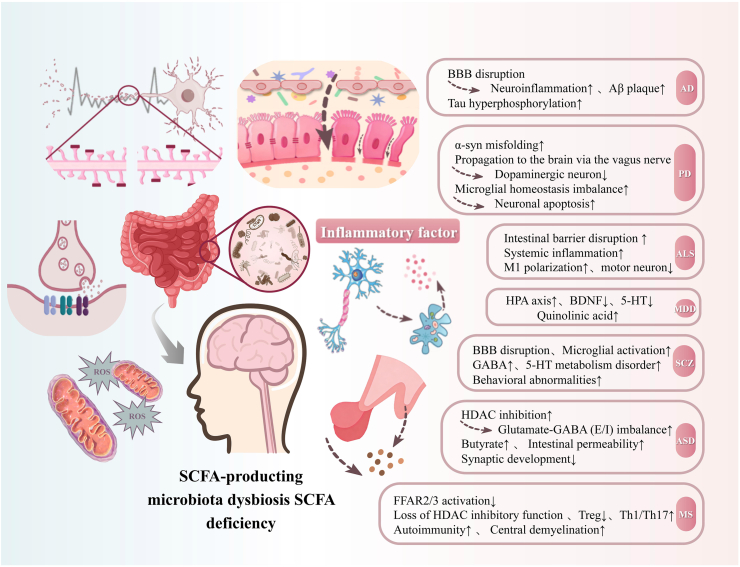
Pathological mechanism of neural injury mediated by dysbiosis of SCFA-producing microbiota This figure illustrates the pathological mechanisms of neuroinjury mediated by dysbiosis of SCFA-producing microbiota and SCFA deficiency via the microbiota-gut-brain axis. Impaired gut and blood-brain barrier integrity, along with elevated inflammatory factors and reactive oxygen species (ROS), drives disease-specific pathological cascades.

SCFA-producing microbiota imbalance is a key factor contributing to neurological injury mediated by the microbiota-gut-brain axis. Its pathological cascade revolves around four major intervention targets, most of which have been cross-validated through microbial colonization, depletion, and SCFA supplementation experiments. These targets represent the primary action sites for the neuroprotective effects of engineered food-derived polysaccharides. All four targets share insufficient SCFA production as the common initiating event, forming a progressive pathological chain that spans multiple signaling pathways and injury stages with well-established causal relationships (Fig. 4). Based on the aforementioned common pathological pathways of neurological injury mediated by SCFA-producing microbiota dysbiosis, this chapter systematically elucidates the shared microbial characteristics and disease-specific pathological mechanisms of SCFA-producing microbiota dysbiosis in neurological injury-related disorders. It clarifies the differences in microbiota-gut-brain axis regulation and target differentiation across various diseases, identifies current gaps in the field and future research directions, and provides a comprehensive theoretical basis for the disease-specific precise intervention of engineered food-derived polysaccharides.

### 3.1 Characteristic alterations of SCFA-producing microbiota in nerve injury-associated diseases

Similar microbiota characteristics and metabolic abnormalities are observed in four major neurological disorders including AD, PD, MDD, and PSCI. Both the abundance and metabolic activity of SCFA-producing functional bacteria are downregulated. The abundances of acetate-producing bacteria such as *Bifidobacterium* and *Blautia*, as well as butyrate-producing bacteria including members of the family Ruminococcaceae and *Ruminococcus*, are significantly reduced. The abundances of pro-inflammatory and potentially pathogenic bacteria are abnormally elevated, creating ecological niche competition with SCFA-producing bacteria and further exacerbating metabolic disorders. Concomitant with these microbiota changes, total SCFA concentrations in feces and peripheral blood are significantly decreased, with butyrate and propionate showing the most pronounced reductions. SCFA levels are significantly negatively correlated with the severity of neurological injury. These shared alterations are represent disease-associated phenotypes rather than universal causal drivers. The key difference lies in the highly specific patterns of microbiota dysregulation, SCFA metabolic profiles, and pathological driving pathways across the four disorders. Neurological injury is not simply caused by SCFA deficiency, which provides the rationale for developing precise, disease-tailored intervention strategies.

In stroke model mice receiving fecal microbiota transplantation from patients with post-stroke cognitive impairment, the abundance of Enterobacteriaceae increases markedly, accompanied by severe neurological damage including blood-brain barrier disruption and excessive microglial activation (Liu et al., 2022a). The abundance of harmful genera such as *Bacillus* and *Staphylococcus* also rises synchronously in cerebral ischemia models (Bertin et al., 2025). Excessive proliferation of these pathogenic bacteria further competitively excludes SCFA-producing microbiota and forms a self-sustaining vicious cycle. This pathological process can be specifically targeted and blocked by intervention with engineered polysaccharides. Nevertheless, accumulating evidence indicates that certain traditional pathogenic bacteria may exert metabolic redundancy and immunomodulatory functions under specific microenvironmental conditions. Their pro-inflammatory effects should be comprehensively evaluated according to host immune status and local metabolic characteristics (Chib et al., 2024; Functional Pathways of the Gut). Current studies fail to clarify whether the enrichment of pathogenic bacteria acts as the cause or consequence of intestinal microbiota dysbiosis. Most relevant investigations only remain at the level of correlation analysis and lack verification of the independent pathogenic role of these bacteria through monostrain colonization experiments.

Structural disruption of microbial communities leads to impaired SCFA synthesis, which serves as a critical link in the occurrence and progression of neurological injury. Reduced production of SCFAs in feces and peripheral circulation has been widely observed in various neurological injury animal models and human cohorts, with butyrate and propionate exhibiting the most prominent declines (Liu et al., 2022a; Dong and Cui, 2022; Ajith and Sreejith, 2026). Insufficient SCFA synthesis induces partial loss of multiple physiological functions of these metabolites. Such functions include anti-inflammation, barrier protection and modulation of neural plasticity, which further trigger the subsequent cascade of neurological injury (Ni et al., 2025). Experiments with exogenous supplementation of sodium butyrate and sodium propionate confirm that the restoration of SCFA levels can remarkably reverse cognitive impairment and neuroinflammation in PSCI and AD models. These findings indirectly verify that SCFA deficiency constitutes an essential contributor to neurological injury. Research conducted by Chen and colleagues demonstrates that exogenous SCFA supplementation aggravates α-synuclein (α-syn) aggregation and neuroinflammation via the GPR43-NLRP3 pathway in 1-methyl-4-phenyl-1,2,3,6-tetrahydropyridine (MPTP)-induced Parkinson's disease mouse models. This phenomenon suggests that SCFAs may exert dual regulatory roles in different neurodegenerative disorders. The functional orientation of SCFAs highly depends on receptor expression profiles and the activation state of downstream signaling networks (An exploratory analysis on the). Considerable heterogeneity exists among the conclusions of published studies. Several cohort investigations on Alzheimer's disease and major depressive disorder do not detect significant alterations in fecal butyrate concentrations. Such heterogeneity is closely associated with differences in baseline dietary patterns, disease progression stages, detection methodologies and the composition of host intestinal microbiota, and no unified consensus has been reached to date.

A significant positive correlation between the degree of intestinal microbiota dysbiosis and the severity of neurological injury has been recognized as a common feature in most animal models and partial human cohorts of neurological injury-related diseases. In a repetitive mild traumatic brain injury (rMTBI) model, fecal microbiota transplantation from model rats into healthy recipients induces typical intestinal microbiota dysbiosis in normal rats. The recipient animals also display anxiety-like behaviors and reduced BDNF expression (M et al., 2025). In mouse models of postpartum depression, the extent of microbiota dysbiosis is directly correlated with hippocampal neuroinflammation and the severity of depression-like behaviors (Gut microbiome). In addition, the application of prebiotics to restore the homeostasis of SCFA-producing microbiota can elevate SCFA concentrations, alleviate neuroinflammation and improve cognitive and behavioral phenotypes simultaneously. These outcomes provide theoretical support for the application of engineered polysaccharides. The regulatory effects of SCFAs on microglia exhibit strong context dependence. Under conditions of chronic stress or aging, butyrate may unexpectedly activate pro-inflammatory microglial phenotypes through HDAC inhibition rather than classical anti-inflammatory pathways (Chaudhry et al., 2023). This finding implies that interventions based on engineered polysaccharides should take host age, stress history and immune baseline status into comprehensive consideration.

### 3.2 Specific pathological mechanisms of SCFA-producing microbiota dysbiosis in distinct neurological disorders associated with nerve injury

Dysbiosis in the composition and function of short-chain fatty acid (SCFA)-producing microbiota presents prominent disease-specific characteristics in neurological disorders related to nerve injury. Such microbial disorders are not passive concomitant phenomena during disease progression. This section describes the specific pathological mechanisms of four major disorders, namely AD, PD, MDD, and PSCI. Intervention targets unique to each disease and distinct from common pathways are analyzed to provide a theoretical basis for disease-differentiated precision design of engineered food-derived polysaccharides.

#### 3.2.1 Stage-specific dysbiosis of SCFA-producing microbiota in Alzheimer's disease and associated pathological mechanisms

Alterations in gut SCFA-producing microbiota in patients with AD show high stage specificity, which represents a key microbial feature distinguishing AD from other neurological disorders. At the preclinical mild cognitive impairment (MCI) stage of AD, the abundance of dominant SCFA-producing microbiota, including *Bacteroides*, *Prevotella*, and *Lachnospira*, is significantly reduced. Lower abundance of butyrate-producing bacteria is correlated with higher Aβ plaque load in the brain and tau protein levels in cerebrospinal fluid (Bertin et al., 2025), which may serve as a specific microbial biomarker for early screening of AD.

Dysbiosis of SCFA-producing microbiota drives disease progression mainly through two AD-specific pathological pathways. Imbalanced SCFA profiles regulate the transcription and enzymatic activity of beta-secretase 1 (BACE1) in the brain. BACE1 acts as the rate-limiting enzyme for Aβ generation and participates in the pathological process of AD (Zhang et al., 2025a). Deficiency of butyrate and propionate impairs the phagocytic clearance of Aβ plaques by microglia. This effect is independent of systemic broad-spectrum anti-inflammatory responses and represents a characteristic central immune disorder during AD progression. Existing animal experiments show that exogenous supplementation of SCFAs effectively restores microglial phagocytic function, reduces Aβ plaque deposition in the brain, and alleviates cognitive impairment (Gold et al., 2024). Food-derived polysaccharides such as *Poria cocos* polysaccharide can also elevate SCFA levels by selectively enriching SCFA-producing microbiota, significantly downregulate BACE1 expression, and reverse cognitive deficits in AD mouse models (Song et al., 2025a).

Distinct SCFA subtypes exert heterogeneous effects during AD pathogenesis, which are mainly determined by disease stage and pathological status and account for inconsistent conclusions across studies. Butyrate is the SCFA subtype with the most robust neuroprotective evidence in AD and stably regulates Aβ clearance throughout the entire disease course. Propionate shows dual regulatory effects dependent on concentration and microenvironment. Supraphysiological concentrations of propionate may exacerbate cerebral amyloid angiopathy, whereas physiological concentrations exert regulatory effects related to Aβ pathology. The effects of acetate strictly depend on disease stage and intestinal barrier integrity. At the preclinical stage of AD with an intact intestinal barrier, physiological concentrations of acetate regulate hippocampal neuronal activity and improve cognitive function via the vagus nerve pathway. In advanced AD with impaired intestinal barrier integrity, acetate readily enters the circulation, induces peripheral inflammation, and aggravates neurodegeneration, leading to divergent conclusions regarding acetate function in different studies. The anti-inflammatory and antioxidant effects of isobutyrate are limited to peripheral intestinal inflammation models and fail to generate effective central regulation. Valerate, formate, and other SCFAs only inhibit inflammatory cytokine secretion by glial cells in vitro, and their in vivo activity cannot be stably exerted due to crosstalk between signaling pathways. Collectively, the biological effects of SCFAs are co-regulated by concentration, target cell receptors, and disease course, and this complexity is particularly evident during chronic AD progression. Targeted and refined regulation of SCFAs holds greater clinical translation potential than simple exogenous supplementation.

#### 3.2.2 Specific pathological mechanisms of SCFA-producing microbiota dysbiosis in PD

PD is the second most common neurodegenerative disorder. Multiple studies demonstrate that butyrate-producing bacteria, including *Faecalibacterium prausnitzii*, *Roseburia* spp., and *Ruminococcaceae*, are significantly depleted in patients with PD. The reduction in their abundance is negatively correlated with the severity of motor and cognitive impairments in patients (Rust et al., 2025). This microbial phenotype precedes the classic motor symptoms of PD and provides important microbial support for the gut-origin hypothesis of PD.

Transmission of α-synuclein (α-syn) pathology from the gut to the central nervous system via the vagus nerve is a core feature distinguishing PD from other neurodegenerative diseases. Specific metabolic disturbances of SCFA components serve as a critical link connecting microbiota dysbiosis to PD pathogenesis. As a key protective metabolite, butyrate is significantly reduced in fecal samples of patients at the prodromal stage of PD. Animal experiments confirm that butyrate supplementation or remodeling of butyrate-producing microbiota activates the AMP-activated protein kinase (AMPK) pathway, upregulates the expression of intestinal tight junction proteins, repairs the damaged intestinal barrier, and blocks the translocation of harmful mediators such as lipopolysaccharide (LPS) into the bloodstream. This process inhibits the aberrant folding and aggregation of α-syn in the enteric nervous system from the source (Guo et al., 2023). In the central nervous system, butyrate inhibits histone deacetylase activity and the activation of the NOD-like receptor thermal protein domain associated protein 3 (NLRP3) inflammasome, alleviates microglial activation in the substantia nigra, improves neuronal mitochondrial function, and reduces the apoptosis of dopaminergic neurons (Xu et al., 2025). Butyrate ultimately restricts the retrograde gut-brain spread of α-syn through the vagus nerve pathway and exerts neuroprotective effects.

Acetate shows a biphasic, stage-dependent fluctuation in PD. Fecal and serum acetate levels are markedly decreased in early-stage patients. In advanced stages, severe impairment of the intestinal barrier causes abnormal translocation of acetate into the bloodstream, elevating serum acetate levels and accelerating α-syn pathology aggregation. Fecal propionate levels are generally decreased in patients with PD, and the magnitude of reduction is closely related to the severity of cognitive impairment. However, some cohort studies have not detected significant changes in propionate levels, leading to heterogeneous conclusions (Ravi et al., 2025). Animal experiments reveal that imidazole propionate, a derivative of propionate, abnormally accumulates in the gut and peripheral blood of patients with PD. This metabolite enters the brain through systemic circulation, activates the mammalian target of rapamycin complex 1 (mTORC1) pathway, induces dopaminergic neuronal death, and amplifies neuroinflammation, representing a specific toxic metabolite in PD (Park et al., 2025). Human cohort studies have only linked its accumulation to PD severity without establishing a causal relationship. In addition, branched-chain SCFAs such as isobutyrate and isovalerate are synchronously reduced in patients with PD, and abnormalities can be detected at the prodromal stage. Reduced levels of these metabolites further disrupt intestinal microecological homeostasis and promote the initiation of intestinal α-syn pathology (Huang et al., 2023).

Relevant findings indicate that overall reduction in SCFA levels facilitates the translocation of pro-inflammatory substances such as LPS and bacterial amyloid. These substances trigger aberrant aggregation of α-syn in the enteric nervous system. Misfolded α-syn is retrogradely transmitted to the substantia nigra region of the midbrain via the vagus nerve, ultimately leading to selective loss of dopaminergic neurons. This process aligns with the gut-origin hypothesis of PD and represents a core intervention target for engineered polysaccharides. Epidemiological evidence shows that vagotomy reduces the risk of PD in humans, providing important clinical support for this gut-brain transmission pathway (Tysnes et al., 2015).

#### 3.2.3 Specific pathological mechanisms of SCFA-producing microbiota dysbiosis in MDD

MDD is a globally prevalent neuropsychiatric disorder frequently accompanied by marked disturbances in gut microbiota structure. The primary characteristic is the significant reduction in the abundance of dominant butyrate-producing bacteria of the order Clostridiales, including *Faecalibacterium* and *Roseburia*. Decreased abundance of these butyrate-producing microbiota directly downregulates the expression of butyrate synthesis genes, including the butyrate synthesis gene (*but*) and butyrate kinase gene (*buk*). This reduction leads to decreased butyrate levels in feces and peripheral blood, and butyrate concentrations are negatively correlated with the severity of clinical depressive symptoms. The pathogenesis of MDD centers on neuroendocrine disorders and tryptophan metabolic shifts mediated by imbalanced SCFA-producing microbiota. Dysfunction of hypothalamic–pituitary–adrenal (HPA) axis negative feedback and tryptophan metabolic shift mediated by indoleamine 2,3-dioxygenase (IDO) are features that distinguish MDD from other neurological disorders. Insufficient SCFA secretion drives neuronal injury through these two pathways and accelerates MDD progression. Different SCFA subtypes exhibit distinct functional profiles in the pathological context of MDD. Protective effects, action boundaries, and potential adverse effects are major determinants of variable response rates to clinical interventions. Butyrate is the SCFA subtype with the most sufficient protective evidence in MDD. Animal experiments show that butyrate intervention at appropriate doses effectively alleviates depressive-like behaviors (Yamawaki et al., 2018). Propionate is the second most important protective metabolite after butyrate and acts simultaneously on neuroendocrine and neurotransmitter metabolic pathways. Propionate inhibits excessive IDO activation to reduce the production of toxic quinolinic acid, maintains central serotonin (5-HT) metabolic homeostasis, repairs the impaired negative feedback function of the HPA axis, and suppresses abnormal cortisol release (Behrens et al., 2024). However, propionate only exerts stable regulatory effects within the physiological concentration range and tends to lose protective activity in pathological microenvironments. The effects of acetate are strictly dependent on host status. In MDD subtypes with intact intestinal barrier and low inflammation levels, physiological concentrations of acetate regulate hypothalamic neuronal activity via the vagus nerve pathway, stabilize HPA axis function, and alleviate depressive-like behaviors. In MDD subtypes with damaged intestinal barrier and high inflammation levels, acetate cannot exert stable protective effects and only serves as a reference marker for intestinal microecological disorders. The anti-inflammatory effects of branched-chain SCFAs such as isobutyrate and isovalerate are restricted to the intestine and cannot penetrate the intact blood-brain barrier to participate in central homeostasis regulation. Notably, SCFAs do not exert only positive regulatory effects. During chronic MDD progression, SCFAs display dual characteristics of neuroprotection and neurotoxicity, and their effects are co-regulated by local concentration, receptor expression, and pathological microenvironment. In certain depressive subtypes, SCFAs abnormally regulate microglial function by activating free fatty acid receptor 2/3 (FFAR2/3), exacerbating kynurenine metabolic disturbance and worsening disease severity (Erny et al., 2021). This explains the limited efficacy and significant individual differences of simple exogenous SCFA supplementation, indicating clear limitations of single-supplementation strategies. Precision intervention regimens should be developed based on individual heterogeneity in MDD. Most of these mechanisms are derived from animal model experiments. Only a small number of small-sample clinical trials have verified the antidepressant effects of SCFA-producing microbiota interventions, and high-level evidence from large-sample, multicenter randomized controlled trials (RCTs) is lacking. Furthermore, existing studies have not clarified the core pathways through which SCFA-producing microbiota regulate MDD pathogenesis and cannot distinguish between direct central effects and indirect peripheral effects, which accounts for discrepancies between in vitro and in vivo experimental results.

#### 3.2.4 Specific pathological mechanisms of SCFA-producing microbiota dysbiosis in PSCI

Unlike chronic progressive neurological disorders, PSCI occurs during the acute phase of stroke. The primary manifestation is a sharp decline in the abundance of butyrate-producing bacteria such as *Bacteroides* and *Ruminococcus*, accompanied by abnormal proliferation of pro-inflammatory bacteria including *Enterobacteriaceae*. Following microbial imbalance, fecal and plasma concentrations of butyrate and propionate decrease rapidly. The magnitude of this reduction is negatively correlated with Montreal Cognitive Assessment (MoCA) scores three months after stroke, suggesting that SCFA deficiency may serve as a biomarker for early prediction of PSCI.

Dysbiosis of SCFA-producing microbiota affects PSCI progression through a cascade of primary stroke-induced injury and secondary nerve damage. Stroke itself causes acute disruption of the intestinal barrier. Decreased SCFA levels further reduce the expression of intestinal epithelial tight junction proteins and aggravate leaky gut. Large amounts of endotoxins such as lipopolysaccharide (LPS) enter the systemic circulation, activate systemic inflammatory responses, penetrate the damaged blood-brain barrier into the central nervous system, and specifically activate hippocampal microglia. This process amplifies central neuroinflammation and represents a pathological mechanism distinguishing PSCI from other forms of dementia. XXX et al. confirmed through fecal microbiota transplantation experiments that transplantation of fecal microbiota from PSCI patients into stroke mice recapitulates phenotypes including intestinal barrier damage, elevated serum LPS levels, aggravated hippocampal neuronal apoptosis, and impaired performance in the Morris water maze test. Butyrate supplementation reverses these injuries (Ren et al., 2025). As an endogenous histone deacetylase (HDAC) inhibitor, insufficient butyrate suppresses the transcription of brain-derived neurotrophic factor (BDNF) and hinders the reconstruction of synaptic plasticity (Rahmani et al., 2025). Propionate and acetate also participate in the regulation of neural repair in PSCI, mainly by modulating vagus nerve signaling to affect neural repair efficiency (Xi et al., 2026). Recent studies have found that in addition to SCFAs, microbiota-host co-metabolites such as ursodeoxycholic acid (UDCA) exert protective effects in PSCI. Reduced plasma UDCA levels are significantly associated with cognitive decline. Animal experiments show that exogenous UDCA supplementation alleviates microglial activation and improves memory function (Zhang et al., 2024a), indicating that multiple metabolic pathways may coordinately contribute to PSCI pathogenesis.

Current studies have not systematically adjusted for confounding factors including post-stroke diet, medication, and bed rest status. It remains unclear whether microbiota dysbiosis is a concomitant result of stroke or a driving factor for cognitive impairment in PSCI. In addition, most studies focus on chronic-phase interventions and lack target validation and interventional research during the golden window for acute-phase neural repair, which ultimately hinders the clinical translation of relevant findings.

#### 3.2.5 Specific differences in MGB axis regulation and intervention window characteristics across the four neurological disorders

Dysregulation of MGB axis represents a common pathological basis for AD, PD, MDD, and PSCI. The divergent regulatory patterns across these four disorders provide a basis for disease-differentiated precision interventions using engineered food-derived polysaccharides. In terms of characteristic differences, AD is defined by selective depletion of *Bacteroides* (phylum Bacteroidetes) and Lachnospiraceae (phylum Firmicutes) at the MCI stage, accompanied by moderate reductions in acetate and propionate synthesis. The intervention pathway targets the cascade of Aβ pathology mediated by aberrant microglial activation, with a focus on defective microglial Aβ phagocytosis. PD is marked by depletion of butyrate-producing microbiota such as *Faecalibacterium* and *Roseburia*, often accompanied by a substantial decline in butyrate synthesis. This depletion triggers retrograde gut-brain transmission and central inflammation induced by misfolded α-syn in the enteric nervous system. PD also involves systemic inflammation activation and aberrant tryptophan metabolism mediated by persistent HPA axis hyperactivity, leading to clinical phenotypes of persistent low mood and anhedonia. MDD is characterized by specific reduction in the abundance of butyrate-producing bacteria such as *Faecalibacterium*. Microbial dysbiosis in PSCI shows striking acute-phase specificity, manifested by a rapid decline in butyrate-producing bacteria and acute proliferation of pro-inflammatory bacteria after stroke. Acute and substantial reduction in SCFAs causes intestinal barrier damage, leading to peripheral inflammation infiltration into the brain and central inflammatory storm, with the clinical phenotype of acute cognitive impairment after stroke. To clearly and intuitively compare the specific differences in microbiota dysbiosis characteristics, metabolic alterations, pathological pathways and intervention targets among the four neurological disorders, their core characteristics are summarized in Table 1.

**Table 1:** Distinct regulatory features of the microbiota-gut-brain axis across four neurological disorders.

Disease	Core SCFA-producing microbiota dysbiosis characteristics	Key pathological pathways	Specific roles of major SCFA subtypes	Core Intervention targets	Major Research limitations
AD	High stage specificity; significant reduction of *Bacteroides*, *Prevotella*, *Lachnospira* at the MCI stage; butyrate-producing bacteria abundance negatively correlated with brain Aβ plaque load and CSF tau levels	1. Regulation of BACE1 transcription and enzymatic activity 2. Impaired microglial phagocytosis of Aβ plaques (independent of systemic inflammation)	Butyrate: Stably regulates Aβ clearance throughout the disease course Propionate: Concentration-dependent; supraphysiological concentrations exacerbate cerebral amyloid angiopathy Acetate: Intestinal barrier-dependent; protective in early stage, pro-inflammatory in advanced stage Isobutyrate、valerate、formate: Only effective peripherally or in vitro	BACE1; Microglial Aβ phagocytic receptors	Unclear concentration-effect relationship of SCFAs; Non-standardized intervention windows across disease stages
PD	Significant depletion of *Faecalibacterium prausnitzii*, *Roseburia* spp., *Ruminococcaceae*; reduction precedes motor symptoms and negatively correlates with motor/cognitive impairment severity	1. Retrograde gut-brain transmission of α-synuclein pathology 2. Intestinal barrier damage and LPS translocation 3. NLRP3 inflammasome activation	Butyrate: Repairs intestinal barrier, inhibits α-syn aggregation, protects dopaminergic neurons Acetate: Biphasic fluctuation; decreased in early stage, elevated in late stage to promote pathology Propionate: Generally decreased; its derivative imidazole propionate has specific neurotoxicity Branched-chain SCFAs: Reduced as early as the prodromal stage	Enteric α-synuclein aggregation; Vagus nerve retrograde transmission pathway	Causal relationship of imidazole propionate not established; unclear dynamic changes of SCFAs across disease stages
MDD	Significant reduction of Clostridiales butyrate-producing bacteria (*Faecalibacterium*, *Roseburia*); downregulated expression of butyrate synthesis genes (*but*, *buk*); butyrate levels negatively correlated with depressive symptom severity	1. Dysfunction of HPA axis negative feedback 2. IDO-mediated tryptophan metabolic shift	Butyrate: Most robust antidepressant evidence Propionate: Inhibits IDO activation, maintains 5-HT homeostasis, stabilizes HPA axis Acetate: Intestinal barrier-dependent; only effective in low-inflammation subtypes Branched-chain SCFAs: Only exert peripheral anti-inflammatory effects, cannot cross the intact blood-brain barrier	IDO enzyme; HPA axis negative feedback regulation	Lack of large-sample multicenter RCT evidence; inability to distinguish direct central effects from indirect peripheral effects
PSCI	Acute sharp decline of *Bacteroides*, *Ruminococcus* in the stroke phase; abnormal proliferation of Enterobacteriaceae; SCFA levels negatively correlated with 3-month MoCA scores	1. Stroke-induced acute intestinal barrier disruption 2. LPS-mediated specific activation of hippocampal microglia 3. BDNF transcription inhibition and impaired synaptic plasticity	Butyrate: HDAC inhibitor, promotes BDNF expression and synaptic repair Propionate、acetate: Regulate vagus nerve signaling to participate in neural repair UDCA (microbiota-host co-metabolite): Also exerts neuroprotective effects	Acute intestinal barrier repair; Hippocampal microglial overactivation	Unadjusted confounding factors (diet, medication, bed rest); lack of target validation for acute-phase interventions

The inter-individual differences in microbial structure and metabolic phenotypes across these diseases reflect distinct driving pathways of nerve injury mediated by the MGB axis in neurological disorders. These differences also prioritize optimal time windows and targets for microbiota intervention in each disease. The optimal intervention window for AD is the preclinical MCI stage, with targets focused on defective microglial Aβ phagocytosis and abnormal BACE1 activity. Intervention for PD should be advanced to the prodromal stage, with emphasis on inhibiting intestinal misfolding of α-syn and blocking gut-brain transmission. Intervention for MDD requires stratification based on inflammatory subtypes, with core targeting of excessive IDO activation and HPA axis dysfunction. PSCI intervention must target the 72-h golden acute phase after stroke, focusing on repairing gut-brain dual barriers and blocking inflammatory cascades. Based on these specific differences, this study establishes a targeted precision intervention system. This work represents an interdisciplinary innovation between food science and neuroscience and provides theoretical and technical support for precision interventions in neurological disorders associated with nerve injury.

## 4 Targeted regulatory characteristics of engineered food-derived polysaccharides as precision prebiotics

Natural prebiotic activity of food-derived polysaccharides is limited by poor targeting, low bioavailability, and large individual variability. Engineered modification technologies can break these bottlenecks by precisely regulating polysaccharides’ fine molecular structures, endowing them with high selectivity for target SCFA-producing microbiota, and realizing the upgrade from “broad-spectrum prebiotics” to “precision prebiotics”. This chapter systematically elucidates the structure-activity relationship, core modification strategies, targeted regulatory mechanisms on SCFA-producing microbiota, and multi-dimensional technical system for efficacy evaluation of engineered food-derived polysaccharides. We systematically collated representative studies on the structural modification, microbiota targeting characteristics and prebiotic activity of food-derived polysaccharides, with detailed information on their engineered modification strategies, core structural changes, targeted SCFA-producing microbiota, and corresponding prebiotic efficacy comprehensively summarized in Table 2.

**Table 2:** Polysaccharide modification & SCFA-Producing microbiota regulation.

Serial No.	Original Source of Polysaccharides	Engineered Modification Strategy	Core Structural Changes After Modification	Core SCFA-Producing Microbiota Targeted for Regulation	Advantages of Targeted Regulation	SCFA Type	Core Prebiotic Activity and Technical Advantages	Reference No.
1	Huangshui Polysaccharide	In vitro fecal fermentation combined with pure strain model	No explicit chemical modification, with emphasis on metabolic pathway analysis	*Bacteroides*, *Phocaeicola*	Enhanced propionate production and regulation of microbial homeostasis	Propionate, Acetate	High fermentability, selective enrichment of acid-producing bacteria, suitable for precision nutritional intervention	Wu et al. (2026b)
2	*Armeniaca sibirica* (Apricot Kernel)	Hot water extraction + structural characterization	Containing a backbone of →3,6)-α-D-Gal-(1→with a triple-helical structure	*Bifidobacterium*, *Lactobacillus*	Reduced F/B ratio and improved microbial diversity	Acetate, Butyrate	Significantly higher SCFA yield than inulin, stable structure, suitable as a functional food raw material	(Effects of almond (Armeniaca Sibirica L)
3	*Bifidobacterium* EPS DA-LAIM	Fermentative extraction + purification	Glucomannan containing 2-, 6-and 2,6-mannose residues	*Lactic acid bacteria*, *Bifidobacterium*	Immune stimulation + prebiotic synergy	Lactic acid, Acetate	Resistance to gastrointestinal environment, strong adhesion ability, antioxidant property, with dual functions of prebiotics and immunoregulation	(Structural analysis and prebiotic activity)
4	Plant by-products (fruits/cereals/tubers)	Physical/chemical pretreatment+in vitro fermentation evaluation	Reduced molecular weight and exposed branched chain structure	*Roseburia*, *Akkermansia*, *Bifidobacterium*	Increased total SCFA yield and promoted phenolic biotransformation	Acetate, Propionate, Butyrate	High demand for standardization of methodological framework, with potential for developing next-generation prebiotics	(Conceptual and methodological approaches applied)
5	*Lycium barbarum* (Goji Berry)	Comparison of different extraction methods (hot water/enzymatic hydrolysis, etc.)	Medium molecular weight, rich in galactose and rhamnose	*Butyricicoccus*, *Ruminococcus*	Sustained-release fermentation for continuous butyrate production	Butyrate (predominant)	Clear structure-activity relationship was observed, where high UA content exhibits antioxidant properties and sustained-release fermentation is dominated by butyrate	(Comparative Study on In Vitro)
6	Citrus Pectin	Low molecular weight natural structure	Linear glucan backbone with low MW	*Bacteroides thetaiotaomicron*, *Ba. fragilis*	Rapid utilization and accelerated propionate accumulation	Propionate (predominant)	Fast fermentation rate, suitable for rapid regulation of intestinal pH and energy metabolism	Wu et al. (2025a)
8	*Ganoderma* spp. (Ganoderma)	Green extraction (ultrasound/enzymatic method)	Predominantly β-glucan, partially with triple-helical structure	*Lactobacillus*, *Akkermansia*	Dual pathways of immunoregulation and neuroprotection	Acetate, Butyrate	Bidirectional regulation through TLR signaling and the gut-brain axis, with the homology of medicine and food	Wang et al. (2022a)
9	*Isaria cicadae* (Cicada Flower)	Ultrasound-assisted extraction	Increased contents of UA, Ara and Gal	*Lactobacillus*, *Akkermansia*, *Clostridium*	Enhanced intestinal barrier and immune response	Total SCFA increased	Antioxidant activity is positively correlated with prebiotic potential, and U-ICM is the optimal process	Guo et al., 2025b
10	Mushroom β-glucan	A variety of advanced extraction technologies (microwave/enzymatic/subcritical)	Activity determined by linkage types (β-1,3/1,6) and solubility	*Bifidobacterium*, *Lactobacillus*	Anti-inflammatory, antiviral and blood glucose regulation	Diverse SCFAs	α-glucan and chitin also have prebiotic potential, and structural diversity supports customized development	(Differences in structure)
11	Raspberry Pectin	Comparison of enzymatic/acid/alkali/ultrasound extraction	Activity affected by RG-I ratio and Rha/GalA ratio	*Bacteroides*, *Phocaeicola*, *Bifidobacterium*	Significant increase in propionate production	Propionate (especially EN-RP/AL-RP)	Structural parameters (DE, neutral sugar content) are strongly correlated with prebiotic activity	Sun et al. (2026)
12	Tamarind Seed	Enzymatic preparation of low molecular weight hydrolysate	Mw reduced from 5.36 × 10^5^ to 4.05 × 10^4^ with skeleton retained	*Lactobacillus* (ETSP2), *Bacteroides* (ETSP1)	MW-dependent targeting of different bacterial genera	Acetate, Valerate (ETSP1)	Low MW promotes *Lactobacillus* more; medium MW promotes *Bacteroides* and total SCFA yield	Zhao et al. (2026)
13	*Saccharina japonica* (Kelp)	Review of multiple extraction methods	Activity affected by sulfate group/uronic acid content	*Bacteroides*, *Lachnospiraceae*	Immunoregulation + anti-tumor + prebiotic effect	Acetate, Propionate, Butyrate	Combination of traditional theory of medicine and food with modern mechanisms, suitable for functional foods and pharmaceutical excipients	Karra et al. (2020)
14	*Coix lachryma-jobi* (Coix Seed)	Steam explosion extraction	Narrow MW distribution (Mw/Mn = 1.47) with increased β-glucan	SCFA-producer genera	Strong fat or cholesterol-binding capacity and pancreatic lipase inhibition	Highest total SCFA (AHP-SE)	Physical modification improves purity and function, and steam explosion is an efficient green process	Ke et al. (2025)
15	Konjac Glucomannan (KGM)	Acetylation modification	Introduction of acetyl groups with reduced viscosity and gel strength	*Prevotella_9*, *Escherichia-Shigella*	Lower F/B ratio and targeted inhibition of pathogenic bacteria	Slight decrease in total SCFA but optimized microbiota	First to reveal the specific response of *Prevotella_9*, and molecular modification achieves precise regulation of microbiota	(Polysaccharide from Artocarpus heterophyllus Lam)
16	*Artocarpus heterophyllus* (Jackfruit)	Hot water extraction	Acidic polysaccharide with uncharacterized structure	*Lactobacillus*, *Lachnospiraceae*	Regulation of co-metabolites (bile acids, indoles)	SCFAs (not specified in detail)	Remodeling of host-microbiota metabolic network with in-depth immunoregulatory mechanism	(Marine-Algal-Derived Postbiotics Modulating the Gut)
17	Seaweed-derived postbiotics (brown algae/red algae)	Direct application of natural polysaccharides	Rich in fucoidan, alginic acid, carrageenan, etc.	*Akkermansia*, *Lactobacillus*, *Bacteroides*	Reduction of endotoxin-producing bacteria and anti-obesity-related inflammation	Total SCFA increased	Acting on the “gut-adipose axis”, with both SCFA production and promotion of adipose browning	Zhang et al., 2025b
18	*Hibiscus mutabilis* Flower	Isolation and purification of acidic polysaccharide S-AMFP	Initially characterized as acidic heteropolysaccharide	Unspecified, but IgA/IgG/IgM↑, CD4^+^/CD8^+^↑	Restoration of CTX-induced immunosuppression	Total SCFA increased	Integration of microbiota-metabolome-immune indicators, with a multi-dimensional immune reconstruction mechanism	(Bupleuri Radix Polysaccharides Alleviate MASLD)
19	*Bupleuri Radix* (Bupleurum Root)	Hot water extraction	Rich in substances promoting *Muribaculaceae*	*Muribaculaceae* (e.g., *P. intestinale*)	Activation of hepatic AMPK and promotion of cholesterol metabolism	Acetate, Propionate	First to clarify the SCFA hepatic metabolic pathway mediated by *Muribaculaceae* in the “gut-liver axis"	Chen et al. (2026b)
20	*Ganoderma lucidum* (Reishi Mushroom)	Traditional extraction	Predominantly β-glucan	*Lactobacillus*, F/B ratio	Inhibition of microglial activation and increase in BDNF	SCFAs (promoting anti-inflammation)	Improvement of age-related cognitive impairment through the gut-brain axis, with mechanisms involving neuroinflammation and synaptic proteins	Qiu et al. (2025)
21	*Poria cocos* (Indian Bread)	In vitro fermentation with child fecal microbiota	Unmodified natural neutral polysaccharide	*Bifidobacterium*, *Limosilactobacillus*	Promotion of acetate production (especially in normal weight children)	Acetate, Lactic acid, Indole lactic acid	Specific response of child microbiota, and indole lactic acid is a newly discovered metabolic marker	Xia et al. (2025)
22	Jinhua Zangcha (Golden Flower Zang Tea)	Ultrasonic treatment (200W)	MW decreased by 34%, UA increased to 274.28 mg/g	*Bacteroides*, *Lachnospira*	Significant increase in butyrate production	Butyrate (7.63 mg/g)	Power-adjustable to balance structural stability and activity, and targeted increase in specific SCFA	Chen et al. (2024)
23	*Rosa roxburghii* (Roxburgh Rose) Pomace	Ultrasound-assisted extraction	Significantly reduced MW with unchanged backbone structure	Diverse beneficial microbiota	Stimulation of beneficial metabolites such as curcumin	Total SCFA increased	Power control to achieve “structural fine-tuning - functional optimization”, and short-time ultrasound (RRTP-US-S) has the best SCFA-promoting effect	Dong et al. (2025)
24	Ginseng Neutral Polysaccharide	Complex enzymatic hydrolysis	Reduced MW, altered surface morphology and increased amorphous region	*Lactobacillus*, *Prevotella*, *Ruminococcus*	Significant inhibition of pathogenic bacteria such as *Enterococcus*	Total SCFA increased	Triple activities of neuroprotection, antioxidant property and prebiotic effect; enzymatic method activates “neglected” neutral polysaccharides	Zhao et al. (2021)
25	Apple Pectin/Homogalacturonan (HG)	Enzymatic degradation to prepare low MW fractions	Low MW AP (60.3 kDa) and HG (861 Da)	*Bifidobacterium*, *Megasphaera*, *Allisonella*	Highest total SCFA yield	Total SCFA (230.40 mmol/L)	Confirmed that low MW is a prerequisite for efficient fermentation, and established a “structure-MW-microbiota-health” relationship model	(Effects of Simulated In Vitro)
26	*Anemarrhena asphodeloides* (Anemarrhena Rhizome)	Fermentation after simulated digestion	Stable structure after digestion with no monosaccharide release	*Prevotella*, *Faecalibacterium*, *Megasphaera*	Inhibition of pathogenic bacteria and maintenance of α-glucosidase inhibitory activity	Total SCFA increased	Digestive stability ensures colonic delivery and targeted regulation of *Faecalibacterium* (a butyrate producer)	Yang et al. (2024b)
27	Moringa Leaf	UV/H_2_O_2_ degradation (3 h)	Reduced MW, decolorization and increased solubility	Dominant microbiota (not specified in detail)	Synchronous enhancement of antioxidant capacity	Total SCFA increased	Oxidative degradation realizes simultaneous purification and activation, providing a modification paradigm for dark plant polysaccharides	(Structural characterization of raw and)
28	*Polygonatum cyrtonema* (Polygonatum)	Steaming processing with wine	Increased oligosaccharide content, reduced MW and increased reducing sugar	*Bifidobacterium*, *Bacteroides*, *Roseburia*	Restoration of immune organ index, superior to crude product	Fecal SCFA increased	Scientific explanation of traditional processing technology shows that structural simplification leads to enhanced prebiotic activity	Zhuang et al. (2024)
29	Wheat Bran	Dual-enzymatic hydrolysis (alkaline protease + *Bacillus* hydrolase)	Production of <21.1 kDa proteins and 130 kDa polysaccharide fragments	Early fermentation microbiota (6h) + butyrate-producing bacteria (24h)	Rapid early SCFA production + late butyrate predominance	Butyrate (24h peak)	First to reveal the “temporal fermentation” characteristic of enzymatic hydrolysates, and MW fragmentation drives stage specificity	Guo et al. (2026b)
30	*Brassica rapa* L. (Chinese Cabbage)	Ultrasound-H_2_O_2_ degradation+in vitro continuous fermentation	Mw converted to monodisperse 1983 Da, decreased Fuc and increased Xyl	*Lachnoclostridium*, *Faecalibacterium*	Reduced Firmicutes/Bacteroidota ratio	Total SCFA increased (59.30 mM)	Continuous fermentation simulates the real colonic environment, confirming that structural fragmentation leads to microbiota reconstruction, which in turn results in an explosive increase in SCFAs	Guo et al. (2026c)

### 4.1 Molecular design principles of precision prebiotics targeting SCFA-producing microbiota

In terms of design, the first approach involves mimicking the spatial conformation and charge distribution of the target strain's natural polysaccharide ligands to align with the recognition characteristics of sensors and transporters within the Polysaccharide Utilization Loci (PULs) system. An example is the specific recognition of sulfated arabinogalactan by Bacteroides spp. (Wu et al., 2026a). To meet the functional requirements for SCFA production, specific design must also align with the metabolic pathway characteristics of the bacterial strains. Cockburn et al. employed a combination of in vitro fecal fermentation and pure culture models to systematically analyze the targeted regulatory mechanism of Huangshui polysaccharides on specific gut bacterial strains, confirming that functional-oriented enrichment can be achieved by inducing the butyrate synthesis pathway, rather than through generalized proliferation (Xia et al., 2021). Bedu-Ferrari et al. also emphasized that next-generation prebiotics must demonstrate their selective utilization capacity at the species or even strain level to meet precision nutrition requirements (Bedu-Ferrari et al., 2022).

Second, the design matches the geometric features of the substrate-binding pockets and the arrangement of catalytic residues in the target strain's CAZymes. For example, the substrate preference of GH13 family amylases for α-1,4-glycosidic bonds ensures that only SCFA-producing strains possessing the corresponding degradation systems, such as *Faecalibacterium prausnitzii* and *Parabacteroides distasonis* which can efficiently initiate polysaccharide degradation (Xia et al., 2021; Overbeeke et al., 2022; Rong et al., 2025a).

Ensuring that polysaccharides reach the colon intact is a prerequisite for achieving microbial targeting. Therefore, polysaccharide design involves regulating molecular weight, glycosidic bond configuration, and spatial conformation to reduce the likelihood of hydrolysis by gastric acid, trypsin, and amylase. At the same time, the water solubility and dispersibility of polysaccharides must be optimized to ensure sufficient contact with SCFA-producing microbiota in the colonic lumen, thereby enhancing substrate utilization efficiency. If polysaccharides degrade prematurely in the upper gastrointestinal tract and are utilized by non-target bacterial strains, not only is their targeting capability lost, but they may also disrupt the homeostasis of the gut microbiota, thereby affecting SCFA synthesis efficiency (Zhang et al., 2024b; Combining Gut Microbiota Modulation and). For example, For example, Zhang et al. developed a mitochondria/colon dual-targeting nanoparticle based on inulin modified with alpha-lipoic acid and triphenylphosphine, loaded with cannabidiol (CBD). In a mouse model of sodium dextran sulfate (DSS)-induced colitis, this system not only achieved colonic-targeted accumulation and glutathione-triggered drug release but also significantly enhanced gut microbiota diversity and levels of SCFAs such as acetic acid, propionic acid, and butyric acid, thereby repairing the mucosal barrier and alleviating the inflammatory response (Zhang et al., 2024c). Similarly, the pectin/arabinan microcapsules developed by Wang et al. also enhance the survival rate of probiotics (*C. butyricum*) during gastrointestinal transit through a bilayer structure, thereby achieving controlled release in the colon (Wang et al., 2025b).

Following the precise enrichment of short-chain fatty acid (SCFA)-producing strains and colon-targeted delivery of polysaccharides, adaptive design of neurometabolic pathways associated with SCFA biosynthesis is required to achieve targeted biosynthesis of neuroprotective SCFAs. This approach avoids the ineffective production of non-functional SCFAs, and serves as a core step for precision prebiotics to exert neuroprotective effects. Butyrate acts as an endogenous vital histone deacetylase (HDAC) inhibitor in the human body. Under pathological conditions with impaired intestinal barrier and blood-brain barrier (BBB), butyrate can cross the blood-brain barrier, restore gene expression of cerebral neurons, and reverse the silencing of cognition-related genes, which makes it a pivotal neuroprotective metabolite for brain health. The present design enables polysaccharide structures to specifically match the metabolic characteristics of butyrate-producing strains. Modification of the branched chains and side groups of polysaccharides can guide microbial strains to elevate the partitioning ratio of carbon sources toward butyrate synthesis and reduce excessive generation of acetate, propionate and other intermediate metabolites. Research conducted by **XXX et al** (Rong et al., 2025b). verified that structurally customized polysaccharides can selectively upregulate the expression of butyrate kinase (buk) and phosphotransbutyrylase (ptb). Such regulation specifically activates the butyrate biosynthesis pathway without interfering with other metabolic processes of microbial strains. Unlike the random fermentation mode induced by conventional prebiotics, the proposed strategy firstly enriches functional microbial strains and subsequently realizes directional production of neuroactive effector molecules.

Most patients with neurological disorders present **compromised intestinal barrier integrity** and **unsustainable gut microbiome stability**. Application safety is essential for the successful translation of precision prebiotics into practical applications and must encompass **systematic safety evaluation** covering three core dimensions **including molecular risk regulation** of polysaccharide modification, **intestinal metabolic safety**, and the physiological stability of the gut microbiome. Ensuring molecular safety during the modification process requires that chemical modifications avoid the residual presence of toxic reagents and the generation of harmful byproducts; biological or physical modifications are generally more controllable (Zhang et al., 2024d). Regarding metabolic safety, it is necessary to prevent the accumulation of harmful end products during polysaccharide metabolism; for example, sulfate reduction in sulfated polysaccharides and ammonia production in nitrogen-containing polysaccharides both pose potential toxicity risks (Rusnak et al., 2021; Ballan and Saad, 2021). Regarding microbiome homeostasis safety, long-term intake must not alter the structural characteristics of the core gut microbiota or induce excessive proliferation of opportunistic pathogens. Additionally, it is necessary to assess whether modified groups might influence the expression of antibiotic resistance or virulence genes in gut strains through horizontal gene transfer (Ziemons et al., 2024; Fu et al., 2025). Together, these three aspects constitute a comprehensive molecular safety control system for precision prebiotics and serve as critical constraints for the selection of engineering modification technologies.

### 4.2 Structure–activity relationships and molecular mechanisms by which food-derived polysaccharides regulate SCFA-producing microbiota

Engineered modification of food-derived polysaccharides adopts physical, chemical, biological and combined modification technologies to precisely tailor molecular structure and physicochemical properties, thereby optimizing microbiota targeting ability, fermentation characteristics and bioavailability, and ultimately realizing targeted enrichment of SCFA-producing microbiota and precise regulation of SCFA generation. Different modification strategies possess unique technical advantages and application scenarios.

#### 4.2.1 Molecular weight and glycosidic bond configuration determine the degradation efficiency and recognition range of SCFA-producing microbiota

The utilization of polysaccharides by intestinal SCFA-producing strains originates from the specific recognition and hydrolysis of glycosidic bonds by extracellular CAZymes, which acts as the first molecular screening barrier to define the range of polysaccharide-utilizable strains. Molecular weight and glycosidic bond configuration are crucial structural characteristics that markedly affect the capacity of SCFA-producing microbiota to utilize polysaccharides; by regulating the transport and degradation efficiency of the PULs system, these factors limit the targeted utilization range of intestinal microbiota toward specific polysaccharides and serve as key determinants for target strains to initiate polysaccharide degradation and metabolism. The molecular weight of polysaccharides is highly matched with the substrate size of PULs transporters in target strains, serving as a prerequisite for strain-level precise targeting. For example, in the PUL48 system of *Bacteroides thetaiotaomicron*, the extracellular dextranase BT3087 (a glycoside hydrolase belonging to GH family 66) [6†L16-L18] cleaves high-molecular-weight dextran into smaller oligosaccharides. The activity of Sus-like systems, including PUL48, has been shown to depend on polysaccharide size, and as the molecular weight of dextran increases, bacterial growth rate decreases and lag time increases [9†L17-L25]. Consequently, an increase in glucan molecular weight significantly inhibits the proliferation of this strain (Feasey et al., 2026). From the perspective of disease intervention compatibility, low-molecular-weight polysaccharides can be directly recognized and transported by the PULs system of SCFA-producing bacterial communities, and exhibit the properties of rapid fermentation and immediate efficacy. In contrast, high-molecular-weight polysaccharides must first be degraded into oligosaccharide fragments by extracellular enzymes secreted by the bacterial community before they can be metabolized, and possess the characteristics of long-term sustained release and continuous nutrient supply (Li et al., 2022).

The substrate recognition preference of CAZymes is highly dependent on the three-dimensional topological features of catalytic domains. The synergistic action of geometric topology of active pockets, spatial arrangement of catalytic residues and molecular steric hindrance jointly mediates the selective screening of enzymes for glycosidic bond configurations and branched structures of polysaccharides, which serves as the structural basis for judging the substrate action spectrum of enzyme systems. Numerous studies have shown that the substrate adaptation characteristics of CAZymes can be modified through fine remodeling of the microstructure at the active center. Slight adjustment of single amino acid residues and local structural units can alter catalytic preference and substrate adaptation capability (Nasseri et al., 2024; Yang et al., 2026; Zada et al., 2025). In fungal cellulases of the GH5_5 family, the second-shell residue T100 is involved in regulating the overall geometric configuration of the active pocket. Site-directed mutation at this locus can change the catalytic properties of enzymes, increasing the catalytic activities of cellulase and mannanase by 2.5-fold and 2.0-fold respectively. This confirms that the micro-network of local residues is a key structure regulating substrate preference (Zheng et al., 2026). Structural heterogeneity of homologous enzyme systems also causes differentiation in substrate screening capacity, and significant structural differences in binding clefts exist among enzyme isoforms of the *Penicillium* GH12 family. PsXegD and PsXegE have spatially open substrate binding clefts that can adapt to highly branched xyloglucan substrates. By comparison, PsEglA owns a narrow substrate binding cleft, which can only bind and catalyze the hydrolysis of linear β-1,4-glucan backbones. Such inherent structural differences determine the tolerance threshold for polysaccharide branched structures and substrate selectivity of different enzyme isoforms (Sun et al., 2024a). In addition, although all members of the GH1 family have a conserved TIM-barrel folding skeleton and high overall structural homology, subtle residue variations in the flexible loop region of active sites can induce dynamic remodeling of the microconformation at the active center. This further switches the enzyme's capacity to recognize and catalyze substrates with different glycosidic bonds including β-glucosides and β-galactosides, which fully demonstrates the refined regulatory effect of structural conformational flexibility on the substrate specificity of CAZymes (Kaenying et al., 2023). In general, the substrate recognition function of CAZymes is not independently determined by the primary amino acid sequence, but governed by the synergistic regulation of three-dimensional conformations at multiple hierarchical levels of catalytic domains.

From the perspective of hierarchical microbiota screening, the glycosidic bond configuration of linear backbones defines the range of polysaccharide-utilizing microbiota. Specific glycosidic bonds can only be recognized and hydrolyzed by strains that encode corresponding CAZymes in their genomes, enabling preliminary screening of microbiota at the phylum level for polysaccharides. This process conforms to the targeting demand of phylum-level microbiota regulation in neurological injury diseases. The branched substitution characteristics on the main chain further refine the screening accuracy. Substitution sites, degree of substitution and glycosidic bond types of side chains modulate the binding efficiency between CAZymes and the main chain through steric hindrance effects. Only strains that simultaneously encode matched backbone hydrolases and debranching enzymes can complete the full degradation of polysaccharides. This narrows the screening resolution of target microbiota from the phylum level to the genus and species levels, and provides a prerequisite for precise targeting at the strain level. The O-2/O-3 substitution sites and degree of substitution of arabinose on the β-1,4 backbone of arabinogalactan strongly influence the type of degrading bacterial strain. High-substitution arabinogalactan can be recognized by the specific PUL system of Bacteroides, while low-substitution linear galactan is better suited for the degradative enzyme systems of the Ruminococcaceae. The structural differences resulting from the α-1,6 linear bonds and α-1,3 branch bonds in isomaltodextrin lead to highly branched isomaltodextrin selectively enriching *Bifidobacterium* and *Faecalibacterium*, whereas linear α-1,4-dextrin, due to its lack of specificity, is readily utilized by various intestinal microbiota and cannot achieve targeted regulation of SCFA-producing microbiota (Comparison of Different Soluble Dietary).

#### 4.2.2 Monosaccharide composition and side-chain modifications mediate strain-level selective enrichment of SCFA-producing microbiota

Oligosaccharide fragments produced by the preliminary hydrolysis of extracellular CAZymes need to enter cells through strain-specific polysaccharide transport systems to complete subsequent metabolic utilization. This acts as the second molecular screening barrier determining the efficiency of polysaccharide utilization by strains, and also serves as a vital regulatory node to realize precise targeting at the strain level.

The uptake of polysaccharides by intestinal SCFA-producing strains depends on the SBPs-SusCD transport system encoded by PULs. Substrate-binding proteins (SBPs) are responsible for the specific recognition and capture of oligosaccharide fragments, while the SusCD transmembrane complex undertakes intracellular substrate transport. Chain length and size selectivity govern substrate specificity, and variations in the PULs transport system among different strains can influence the disease-targeting adaptability of polysaccharides. The PUL1689-1703 system in *Bifidobacterium species* encodes the sulfatase BpS1_8 and the SusCD transporter; BpS1_8 specifically hydrolyzes the sulfate ester bonds in arabinogalactan, and this desulfation step is a necessary prerequisite for subsequent sugar chain degradation and transport (Robb et al., 2022). In contrast, the PUL system of *Bifidobacterium longum* utilizes the hydrophobic interactions of the mannose-binding protein BL0033 to recognize the mannose side chains of galactomannan (mannose:glucose = 2:1), thereby enabling the specific uptake and fermentation of this polysaccharide (Liu et al., 2011).

The monosaccharide composition and backbone structure of pectin oligosaccharides exhibit typical structure-dependent regulation of selectivity for SCFA-producing bacterial communities. The homogalacturonic acid (HG) backbone, composed of α-1,4-linked galacturonic acid (GalA), is more readily utilized by *Bacteroides* species for propionate synthesis. The rhamno-galacturonic acid glycan I (RG-I), featuring a rhamnose-GalA disaccharide backbone linked to arabinogalactan side chains, selectively enriches butyrate-producing bacteria such as the Bacteroides and Ruminococcus families (In-Vitro Prebiotic Analysis of Microbiota). Furthermore, the degree of esterification of GalA in pectin not only determines microbial targeting but is also directly related to prebiotic activity. Low-esterified pectin can enhance intestinal barrier protection and anti-inflammatory activity by promoting the proliferation of SCFA-producing bacteria; the molecular mechanism of this process is associated with how the degree of GalA esterification regulates the recognition efficiency of the PULs system (Dietary fiber monosaccharide content alters).

#### 4.2.3 Functional group modifications remodel the targeting property and biological activity of polysaccharides and control associated metabolic risks

Targeted modification of functional groups can reshape the molecular interaction characteristics with the PULs system of SCFA-producing bacterial communities by altering the charge density, spatial conformation, and hydrophobicity of polysaccharides, thereby optimizing targeting and prebiotic activity. However, the modification process requires strict control of potential intestinal metabolic risks, in line with the safety design principles of precision prebiotics (Rational bioengineering of polysaccharide in). Carboxymethylation introduces carboxymethyl groups at the hydroxyl sites of polysaccharides, enhancing water solubility by regulating negative charge density while simultaneously strengthening electrostatic binding with positively charged receptors on the surface of Bifidobacterium species, thereby achieving selective enrichment (Shi et al., 2026). Acetylation modification, on the other hand, regulates the hydrophobicity of polysaccharides by introducing acetyl groups, enhancing interactions with the cell membranes of Clostridium species and specifically promoting butyrate synthesis (Lin et al., 2025a). Sulfation modification introduces negatively charged sulfate groups, altering the spatial conformation of polysaccharides, thereby enabling specific binding to transporters in the PUL system of Bacteroides species and improving propionate synthesis efficiency (Lin et al., 2025b).

Sulfation is a widely used modification method but may also carry considerable metabolic risks. Sulfate groups in natural sulfated polysaccharides, such as fucoidan and alginate, release sulfate ions during intestinal fermentation. As a sulfur source for sulfate-reducing bacteria, these sulfate ions drive the proliferation of such bacterial populations and their metabolism to produce hydrogen sulfide (Luo et al., 2024). Excessive hydrogen sulfide disrupts the expression of tight junction proteins in the intestinal epithelium, induces intestinal oxidative stress and inflammatory responses, and may even inhibit the proliferation of SCFA-producing bacteria (Birg et al., 2023). Therefore, the molecular design of sulfated modifications must strictly regulate the degree of sulfation and the modification sites to ensure targeting of SCFA-producing bacteria while reducing the metabolic utilization efficiency of sulfate-reducing bacteria, thereby achieving a balance between activity and safety (Effects of Pine Pollen Polysaccharides).

#### 4.2.4 Fine structures of polysaccharides shape the metabolism of SCFA-producing microbiota

The fine structure of polysaccharides not only plays a key role in determining the degradation and utilization efficiency of individual SCFA-producing bacterial strains on polysaccharides but also achieves directional regulation of SCFA synthesis by constructing hierarchical metabolic networks of the gut microbiota. The structural characteristics of polysaccharides can screen specific microbial consortia capable of degrading themselves, thereby driving the formation of metabolic collaboration relationships among distinct bacterial strains. From the perspective of metabolic regulation, the inherent metabolic pathways of target strains are major contributors to the final metabolic shunting direction of monosaccharide components. Polysaccharide substrates rich in uronic acid tend to facilitate acetate generation, whereas hemicellulosic polysaccharides enriched with xylose and arabinose are more susceptible to trigger butyrate synthesis. *Bacteroides* usually adopts succinic acid as a key intermediate to produce propionate through the decarboxylation pathway. Butyrate-producing microorganisms including Clostridiales and Ruminococcaceae play a dominant role in the butyrate biosynthesis pathway and can preferentially convert pentoses and other monosaccharides into butyrate.

Such discrepancies in strain metabolic pathways and synergistic features among microbial communities are reflected in the intestinal degradation and metabolic processes of polysaccharides. Xanthan gum typically needs to be first hydrolyzed by enzymes secreted by enzymes secreted by Streptococcus spp. into mannan oligosaccharides, which are then recognized and utilized by the polysaccharide utilization locus system of *Bacteroides* to produce acetate. The generated acetate serves as a metabolic substrate for *Faecalibacterium prausnitzii* and is further converted into butyrate (Differences in fine arabinoxylan structures). The degradation of cereal arabinan depends on multi-strain synergistic effects, requiring the joint action of xylanase from *Bacteroides fragilis* and arabinofuranosidase from *Bifidobacterium adolescentis*. The arabinose released from degradation is metabolized by bifidobacteria to generate lactic acid, which is subsequently converted into butyric acid by *Clostridium butyricum* to complete a sequential metabolic process (Bouiche et al., 2023).

This polysaccharide-structure-mediated microbial interaction network enables different SCFA-producing bacterial strains to form synergistic relationships in terms of nutrition and function, further enhancing the polysaccharide's capacity to directionally regulate SCFA synthesis. The fine structure of polysaccharides can strongly influence the synthesis subtypes and efficiency of downstream SCFAs by regulating the types of upstream degrading strains and the classes of intermediate metabolites (Differences in fine arabinoxylan structures; Jiao et al., 2026).

### 4.3 Core engineered modification strategies for food-derived polysaccharides

**Fig. 5:**
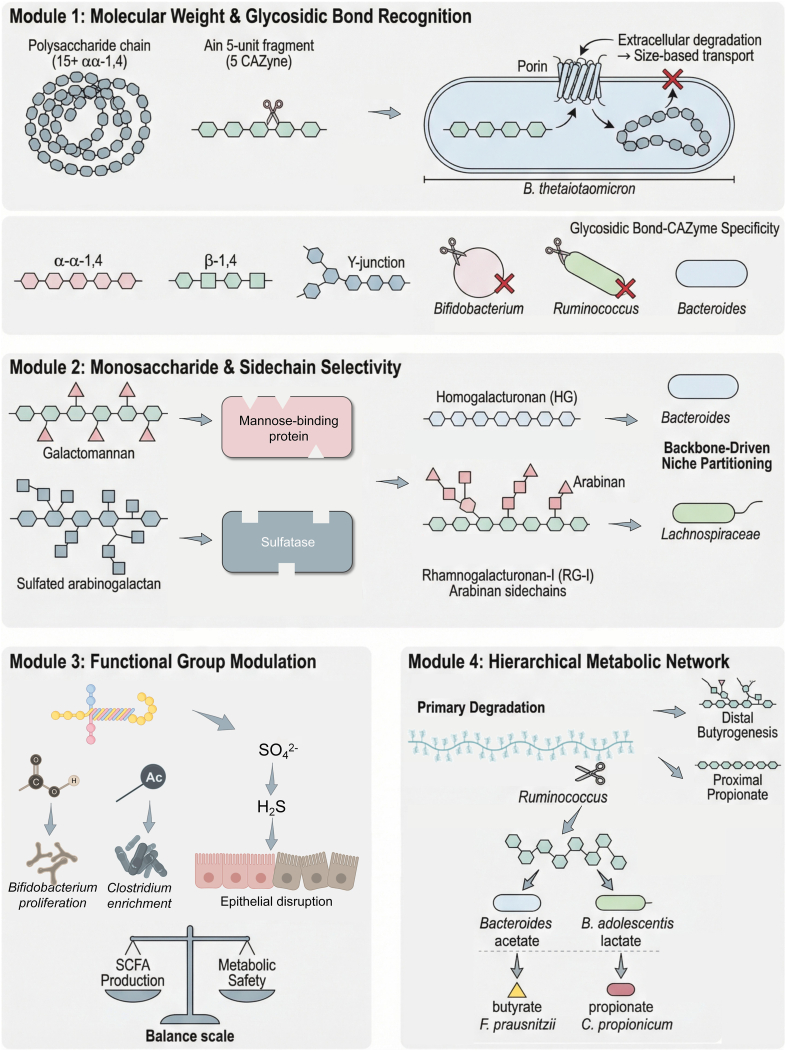
Schematic of Structure-Activity Relationship and Mechanism of Engineered Food-Derived Polysaccharides Regulating SCFA-Producing Microbiota Polysaccharide molecular weight (DP) and glycosidic bond configuration regulate the degradation and targeting of SCFA-producing microbiota via CAZymes and the PULs system, while monosaccharide composition and side chain modifications support strain-specific selection driven altered pectin architectures matched folded focused granted hing infl juxtap lean locked ocular poised rooted slim tapered unified viable buffered calibrated casc constrained congru concealed clustered emergent etched fair bip cross distal graded hom intact meta scarce translating underlying translational encumber baseline degrad attenu accrued afford.

Engineered modification of food-derived polysaccharides serves as a core technical approach for implementing the design principles of targeted prebiotics. Physical, chemical, biological, and composite modification techniques are used to directionally modulate the molecular structures and physicochemical properties of polysaccharides. These methods enable customized optimization of microbial targeting, fermentation performance, and in vivo delivery efficiency, facilitating the directional enrichment of SCFA-producing microbiota and controllable regulation of SCFA biosynthesis. Different modification strategies possess divergent functional mechanisms, technical strengths, and applicable scenarios, and can be flexibly selected and combined according to targeted regulatory requirements. The following figure illustrates the structure-activity relationship rules of engineered food-derived polysaccharides in the targeted regulation of SCFA-producing microbiota by illustrating the four core dimensions of polysaccharide molecular modification (Fig. 5).

#### 4.3.1 Physical modification technologies

Physical modification utilizes mechanical, thermal, or radiation energy to regulate the spatial aggregation state, molecular chain conformation and degree of polymerization distribution of polysaccharides. Throughout the process, the chemical composition of the polysaccharide's functional groups and the types of chemical bonds in the main chain remain unchanged. This method offers the advantages of no chemical reagent residues and high food-grade safety, and acts as a basic technical means for structural customization of precise prebiotics. By adopting diverse physical modification technologies to directionally regulate the target structural characteristics of polysaccharides, it can accurately match the substrate preferences of CAZymes and PULs systems in target strains, and adapt to the intervention demands of different neurological diseases. Ultrasonication, high-pressure homogenization, ultra-high-pressure treatment, pulsed electric fields, and electron beam irradiation are currently the most widely used physical modification methods. Distinct differences exist in the structural regulation capability and disease adaptation scenarios of these various technologies (Jiang et al., 2025a).

Ultrasonic modification primarily utilizes the localized high temperature, high pressure, and shear forces generated by the cavitation effect to achieve directed cleavage of polysaccharide chains, while simultaneously disrupting the hydrogen bond networks between polysaccharide molecules and breaking down dense crystalline and aggregated structures. Studies on the modification of edible mushroom polysaccharides (EMPs) have shown that ultrasonic treatment at 200-400 W for 30-60 min can reduce the weight-average molecular weight of shiitake mushroom polysaccharides from 2.86 × 10^6^ Da to 6.35 × 10^5^ Da, water solubility increases by more than 40%, and the originally compact β-glucan chains are unfolded, exposing more glycosidic bond recognition sites. Edible fungal polysaccharides modified under these conditions can be efficiently recognized by the SusCD transporter and glycosidase in the Bacteroides PULs system, resulting in a 2- to 3-fold increase in the abundance of core SCFA-producing bacteria such as those in the Bacteroidetes phylum and the Bacteroides family, and a 58% increase in total SCFA production in the fermentation system, with the proportion of butyric acid rising from 12% to 21% (Li et al., 2025b). Ultrasonic treatment is also commonly used as a pretreatment step to disrupt the compact structure of polysaccharides, thereby creating optimal reaction conditions for subsequent enzymatic hydrolysis or chemical modification.

Ultra-high pressure (UHP) treatment applies isostatic pressure of 100–600 MPa to disrupt non-covalent bonds within and between polysaccharide molecules—including hydrogen bonds, hydrophobic interactions, and van der Waals forces—thereby altering the chain conformation and crystallinity of polysaccharides without breaking the glycosidic bonds. Studies on oat β-glucan have shown that 15 min of UHP treatment at 300 MPa reduces the crystallinity of β-glucan from 28.3% to 11.7%, significantly increases the radius of gyration of the molecular chains, and substantially enhances its dispersibility in the aqueous environment of the gut. The modified oat β-glucan can be more efficiently degraded and utilized by butyrate-producing bacteria of the families Ruminococcaceae and Spirochaetaceae. After 72 h of in vitro fermentation, butyrate production increased by 47% compared to the unmodified group, while also promoting the conversion of acetate to butyrate in a cross-feeding network (Liu et al., 2026a).

Pulsed electric field (PEF) technology utilizes the electroporation effect and polarization induced by high-voltage pulses to alter the charge distribution and chain conformation of polysaccharide molecules, while simultaneously inducing the rearrangement of intermolecular hydrogen bonds, thereby optimizing the physicochemical properties of polysaccharides under mild conditions. Studies on the modification of konjac glucan indicate that treatment with a pulsed electric field of 20-40 kV/cm for 200-400 μs causes moderate unfolding of the glucan molecular chains, reducing the apparent viscosity while preserving the integrity of the main chain structure. The modified konjac glucan significantly increased the relative abundance of *Bifidobacterium* and *Faecalibacterium* during in vitro fermentation, with total acetic and butyric acid production increasing by 36% compared to the unmodified group, **without disrupting the overall structure of the gut microbiota** (Thikham et al., 2024).

Electron beam irradiation (EBI) can modulate the molecular structure and crystallization properties of polysaccharides through the action of free radicals generated by ionizing radiation. Studies on resistant starch RS5 indicate that electron beam irradiation at doses of 5-10 kGy can effectively break the hydrogen bonds between starch molecules, while simultaneously inducing the formation of V-shaped inclusion complexes between starch molecules and fatty acid ligands, significantly slowing their digestion rate in the small intestine and enabling targeted delivery to the colon. The modified resistant starch serves as a stable fermentation substrate for SCFA-producing bacterial communities in the colon. After 72 h of in vitro fermentation, the in-situ butyrate production in the intestine increased by 62% compared to the unmodified group, while also sustaining the continuous proliferation of butyrate-producing bacteria (Liu et al., 2023).

#### 4.3.2 Chemical modification technologies

Chemical modification precisely regulates the charge distribution, hydrophilicity, spatial conformation, and flexibility of polysaccharides by selectively introducing or replacing functional groups on the polysaccharide molecular chain. The structure of modified polysaccharides can achieve a high degree of compatibility with carbohydrate-binding modules and the substrate preferences of CAZymes, while also optimizing the stability of polysaccharide delivery during in vivo circulation. This ultimately enables the targeted enrichment of SCFA-producing bacterial communities and completes the directional regulation of SCFA synthetic processes. Sulfation, carboxymethylation, acetylation, phosphorylation, and chemical cross-linking are currently the mainstream chemical modification approaches. Through distinct molecular mechanisms, these modifications enable the customized optimization of the prebiotic properties of polysaccharides.

Sulfation modification can directionally introduce negatively charged sulfate groups at hydroxyl sites on polysaccharides, altering their charge density, spatial conformation, and molecular flexibility, while simultaneously endowing polysaccharides with the capacity to specifically bind transporters in the PULs system of Bacteroides and target BACE1 transcriptional regulation and microglial Aβ phagocytosis pathways. This modification adopts C-6 site-specific derivatization with the degree of substitution strictly controlled at ≤0.5. Its target strains include *Bacteroides* and Lachnospira, with prominent advantages in precisely matching the substrate recognition preference of the PULs system, enhancing butyrate synthesis efficiency, regulating BACE1 transcription through HDAC inhibition, and improving microglial Aβ phagocytosis capability. After modifying the active molecular hydroxyl sites of *Lycium barbarum* residue polysaccharides with sulfate groups, the surface negative charge density increases specifically, forming high-affinity binding with lectin-like proteins on the surface of *Bifidobacterium* via electrostatic interactions. This selectively promotes the growth of such SCFA-producing strains and raises acetic acid production in the fermentation system by more than twofold, realizing one-to-one targeted recognition through structural modulation. Meanwhile, chemical modification can accurately replicate the structural characteristics of natural bioactive polysaccharides. The introduced sulfate groups mimic the structural features of natural heparan sulfate and activate the TLR4/NF-κB signaling pathway, which may indirectly shape a favorable microenvironment for the colonization of Bifidobacterium, Bacteroides, their affiliated Clostridium clusters, Lactobacillus and related genera. Consequently, modified polysaccharides exert dual bioactivities of gut microbiota regulation and immune modulation (Liu et al., 2011; In-Vitro Prebiotic Analysis of Microbiota). Nevertheless, sulfated polysaccharides derived from different sources and with varied molecular weights exhibit differential microbiota selectivity, indicating that sulfation degree and sugar unit composition are critical structural determinants of targeting specificity (Zhou et al., 2022; Gao et al., 2025).

Carboxymethylation modification introduces carboxymethyl groups at the hydroxyl sites of polysaccharides, allowing for the controlled regulation of their negative charge properties while significantly enhancing their water solubility and dispersibility. The degree of substitution is precisely optimized within the range of 0.6–0.8, with target strains covering *Faecalibacterium* and *Roseburia*. This modification features superior performance in improving water solubility and negative charge density, matching the CAZymes substrate preference of *Faecalibacterium*, elevating colonic butyrate concentration, inhibiting IDO activity via butyrate to correct tryptophan metabolic disorders, repairing intestinal barrier function, reducing the risk of endotoxin translocation, and exerting synergistic antioxidant and anti-inflammatory effects. The increased water solubility resulting from carboxymethylation allows the polysaccharide to disperse uniformly in the gastrointestinal environment, preventing microbial imbalance caused by localized high concentrations, while significantly increasing the probability of contact between the polysaccharide and SCFA-producing bacteria, ensuring the stability and uniformity of SCFA production. Studies have shown that carboxymethylated hawthorn polysaccharides exhibit more than three times the solubility in simulated gastrointestinal fluid compared to unmodified polysaccharides. In mice, they significantly increase the abundance of SCFA-producing bacteria within the Bacteroidetes and Firmicutes phyla, thereby promoting an increase in total SCFA levels in the gut. Simultaneously, the modified polysaccharides can improve intestinal barrier integrity by upregulating the expression of tight junction proteins, thereby reducing endotoxin translocation. Consequently, in a HepG2 cell model, they significantly enhance the activity of superoxide dismutase and glutathione peroxidase, exerting synergistic antioxidant and anti-inflammatory effects (Subhash et al., 2025). Studies on the carboxymethylation modification of *Poria cocos* polysaccharides indicate that modified polysaccharides with a substitution degree of 0.8 can interact specifically with cation-binding proteins on the surface of *Bacteroides* species via surface negative charges, significantly promoting the proliferation of this key butyrate-producing bacterium and increasing butyrate levels in mouse feces by 52%, further confirming the precise regulatory role of carboxymethylation modification in targeting the microbiota (Ma et al., 2025a).

Acetylation modification, by introducing acetyl groups into the polysaccharide molecules, modulates the hydrophobicity and steric hindrance of the polysaccharides, thereby enhancing their interaction with the bacterial cell membrane. Simultaneously, it regulates the expression of genes related to SCFA synthesis within the bacterial strain through the substitution sites and number of acetyl groups. Studies on *Cyperus rotundus* polysaccharides indicate that acetylated polysaccharides, while reducing the degradation rate during the early fermentation stage, significantly accelerate butyrate accumulation in the late fermentation stage, while selectively enriching *Bacteroides* species. In contrast, the unmodified native polysaccharide tends to promote the growth of *Bifidobacterium* species, directly confirming that acetylation modification can reshape the microbial targeting preferences of polysaccharides by altering their physicochemical properties (Chen et al., 2022a).

Phosphorylation modification introduces phosphate groups into polysaccharide molecules, endowing them with stronger negative charge properties and antioxidant activity, while also optimizing the interaction between polysaccharides and the gut microbiota. Studies on the phosphorylation modification of yam polysaccharides show that phosphorylated yam polysaccharides with a substitution degree of 0.32 exhibit a DPPH radical scavenging rate more than 65% higher than that of unmodified polysaccharides. Additionally, through electrostatic interactions, they enhance binding capacity with surface proteins of Bifidobacterium and Lactobacillus, increasing the abundance of these two types of acetate-producing bacteria by 1.8-fold in in vitro fermentation, and total SCFA production by 42% (Chen et al., 2022b). Studies on the phosphorylation modification of corn cob polysaccharides further revealed that the introduced phosphate groups significantly improve the water solubility of the polysaccharides, while also making the molecular chains more extended, thereby facilitating recognition and degradation by butyrate-producing bacteria of the family Spirochaetaceae. In the fermentation system, the proportion of butyrate increased from 14% to 26%, achieving targeted optimization of SCFA composition.

Chemical cross-linking modification, on the other hand, uses cross-linking agents to construct a three-dimensional network structure for the polysaccharides, endowing them with pH responsiveness and resistance to degradation by gastrointestinal fluids. This enables targeted controlled release of the polysaccharides in the colon, preventing premature degradation in the upper gastrointestinal tract and precisely providing a continuous and stable fermentation substrate for SCFA-producing bacterial communities in the colon, thereby enhancing the targeting and efficiency of in situ SCFA production.

#### 4.3.3 Biological modification technologies

Biological modification relies on the specific catalytic action of enzymes or microorganisms to achieve selective cleavage, side-chain modification, and molecular structural rearrangement of polysaccharide chains under mild conditions. It exhibits outstanding advantages including high reaction specificity, few side reactions, and excellent food-grade safety. This technique enables the precise preparation of polysaccharide fragments that are highly compatible with the PULs of SCFA-producing microbiota, and serves as a technical approach for achieving strain-level targeted regulation and optimized metabolic synthesis of SCFAs. Biological modification mainly includes two pathways including enzymatic modification and microbial fermentation modification. Both approaches achieve precise and directional regulation of polysaccharide molecular structures through distinct catalytic mechanisms.

Enzymatic modification utilizes various CAZymes to conduct directional modification of polysaccharide molecules. Glycosidases, polysaccharide lyases, and glycosyltransferases are the three most widely used enzyme preparations. These three types of enzymes differ completely in substrate specificity and mechanism of action, and enable differentiated regulation of polysaccharide structures. Glycosidases have the widest application range. They can recognize specific glycosidic bond configurations and linkage sites, precisely hydrolyze the main chains and side chains of polysaccharides, and directionally generate oligosaccharide fragments with the target degree of polymerization. No redundant side reactions occur during the process, and product homogeneity is far higher than that of physical modification methods. β-galactosidases of the GH2/GH42 family derived from lactic acid bacteria anchor galactan substrates via carbohydrate-binding modules, screen the stereoconfiguration of sugar rings through a fixed barrel-shaped catalytic pocket, and only adapt to β-configured galactose residues. Meanwhile, active sub-sites can specifically recognize β-1,3 and β-1,4 glycosidic bonds, achieve precise site-specific hydrolysis of substrates, and eliminate non-specific binding to realize controllable enzymatic hydrolysis of galactans. The products can effectively activate the ABC transport system of *Bifidobacterium adolescentis*, specifically promote strain proliferation, and enhance acetate production in the fermentation system (Kittibunchakul et al., 2020). GH10 family xylanases derived from gut commensal bacteria can specifically hydrolyze β-1,4 glycosidic bonds in the arabinoxylan backbone, generating xylooligosaccharide fragments with a degree of polymerization of 3-7. These fragments can be efficiently utilized by butyrate-producing bacteria of the genera Ruminococcus and Bacteroides, thereby specifically increasing intestinal butyrate levels (Mendonça et al., 2023).

Polysaccharide lyases mainly act on acidic polysaccharides containing uronic acids, such as pectin and sodium alginate. They cleave glycosidic bonds via β-elimination reactions while generating unsaturated double bonds at the non-reducing end, and produce oligosaccharide fragments with unsaturated structures without introducing exogenous modifying groups. Studies on citrus pectin show that pectinase derived from *Bacteroides thetaiotaomicron* can specifically cleave α-1,4-glycosidic bonds in the galacturonic acid regions of pectin to generate unsaturated pectin oligosaccharides with a degree of polymerization of 4–8. Compared with natural pectin, oligosaccharide fragments with terminal unsaturated double bonds in modified polysaccharides have stronger spatial extensibility and richer substrate recognition sites. These fragments can precisely match the SusCD transporters and glycoside hydrolases in the PULs of Bacteroides and Bifidobacterium, be specifically utilized by the two genera, and significantly promote propionate and butyrate production (Fu et al., 2021). Glycosyltransferases can introduce specific monosaccharide residues onto polysaccharide molecules through reversible glycosylation reactions, restructure the side-chain architecture of polysaccharides to match the substrate preferences of target strains. By introducing fucose residues onto the glucan main chain via fucosyltransferases, modified polysaccharides can be recognized by the unique PULs of *Propionibacterium*, achieving targeted enrichment of this **butyrate-producing functional** bacterium (Moon et al., 2025).

#### 4.3.4 Composite modification technologies

Composite modification is a technique that synergistically integrates two or more single modification methods to compensate for the inherent limitations of single modification approaches and achieve multi-dimensional and precise customization of polysaccharide fine structures. It can balance modification efficiency, targeting accuracy and delivery stability, and maximize the targeted regulatory effects on SCFA-producing microbiota.

Physicochemical composite modification is the most mature composite modification strategy at present. It can not only realize controllable regulation of polysaccharide molecular weight and directional modification of functional groups, but also balance the water solubility, in vivo stability and microbial targeting performance of polysaccharides. Taking ultrasonic combined with chemical cross-linking modification as an example, ultrasonic pretreatment can unravel the entangled and curled molecular chains of sodium alginate, expose more active sites, and improve the efficiency of subsequent phenolic cross-linking reactions by more than 40%. The dynamic covalent network structure formed by modification exhibits a release rate of less than 15% in simulated gastric and intestinal fluids, and can achieve stepwise complete release in the colonic environment, precisely targeting colonic microbial communities and continuously providing substrates for microbial fermentation (Differences in fine arabinoxylan structures). Similarly, when ultra-high-pressure technology is combined with sulfation to modify *Poria cocos* polysaccharides, ultra-high-pressure pretreatment can promote full unfolding of polysaccharide molecular chains, significantly improve the accessibility of hydroxyl groups, and result in a more uniform degree of sulfation modification. Modified products not only have improved water solubility, but also can specifically promote the proliferation of *Bacteroides* strains while retaining their own immunomodulatory activityy (Ma et al., 2025b).

Bio-chemical composite modification integrates the high specificity of enzymatic hydrolysis and the functional expandability of chemical modification, and has great application potential in the customized transformation of polysaccharides. In a relevant study, cellulase was used to gently hydrolyze corn bran arabinan to prepare uniform oligosaccharide backbones with a degree of polymerization of 4–6. After selenylation modification, the resulting product not only retains the selective growth-promoting effect on *Bacteroides*, but also specifically enhances butyrate production. Meanwhile, the introduced selenium element can activate glutathione peroxidase, enhance the inhibitory ability on gastric cancer cells via the mitochondrial apoptosis pathway, and achieve synergistic enhancement of intestinal microbiota regulation and anti-tumor activity (Fang et al., 2024). Furthermore, after identifying the SusCD complex (the core transport system of target strains) based on metagenomic data, special substrates adapted to target strains were reverse-engineered following the strategy of “preparing specific glucan backbones via endoglycosidases + dual sulfation–acetylation modification”. Experiments confirmed that this modified polysaccharide can only be efficiently uptaken and utilized by target strains, significantly increasing the yield of target SCFAs. Competitive strains such as *Clostridium* species cannot utilize the substrate due to the lack of corresponding transport systems, truly realizing precise regulation at the strain level (Lv et al., 2022; Karcher et al., 2021).

Physical pretreatment can also effectively reduce the cost of biological modification and shorten the reaction cycle, while maintaining the high selectivity of enzymatic hydrolysis. In the ultrasonic-enzymatic hydrolysis combined process, ultrasound disrupts the crystalline regions of polysaccharides, exposes more glycosidic bonds for enzyme binding, and breaks long-chain molecules at the same time, shortening the reaction time and improving product homogeneity. Studies on Tibetan tea polysaccharides show that 200 W ultrasonic pretreatment can reduce its molecular weight by 34%, and uniform oligosaccharides are obtained after xylanase hydrolysis. In vitro fermentation experiments show that this modified product can significantly promote the proliferation of beneficial bacteria such as *Bacteroides* and *Fusobacterium*, and substantially increase the total production of SCFAs (Guo et al., 2026a).

In summary, the pathology-guided precise design of the neuroprotective effects of engineered food-derived polysaccharides requires the establishment of a predictable correspondence between the molecular pathological targets of specific nerve injury diseases and polysaccharide structure parameters. Distinct differences exist among different nerve injury diseases in the severity of intestinal barrier damage, patterns of peripheral immune activation, and central pathological processes. These disease-specific characteristics can in turn affect the selection of polysaccharide molecular weight distribution, degree of branching, charge density, and functional group modification sites. This enables modified polysaccharides to selectively enrich specific SCFA-producing microbiota, and precisely act on central targets matching disease pathology through interactions between microbial metabolites and host signaling pathways. Methods for verifying causal relationships, such as fecal microbiota transplantation, specific strain colonization, and effector molecule signaling pathway blockade, provide preliminary evidence supporting this process from microbial activation to neurobehavioral improvement. Only by taking the target demands of neuropathological mechanisms as the starting point of polysaccharide structure design can potential neuroprotective pathways from microbiota regulation to neuroprotection be realized. This also forms the basis for the systematic discussion of the four core pathways: immune inflammation, endocrine metabolism, direct neural communication, and intestinal barrier function.

### 4.4 Core mechanisms underlying the targeted regulation of SCFA-producing microbiota by engineered food-derived polysaccharides

**Fig. 6:**
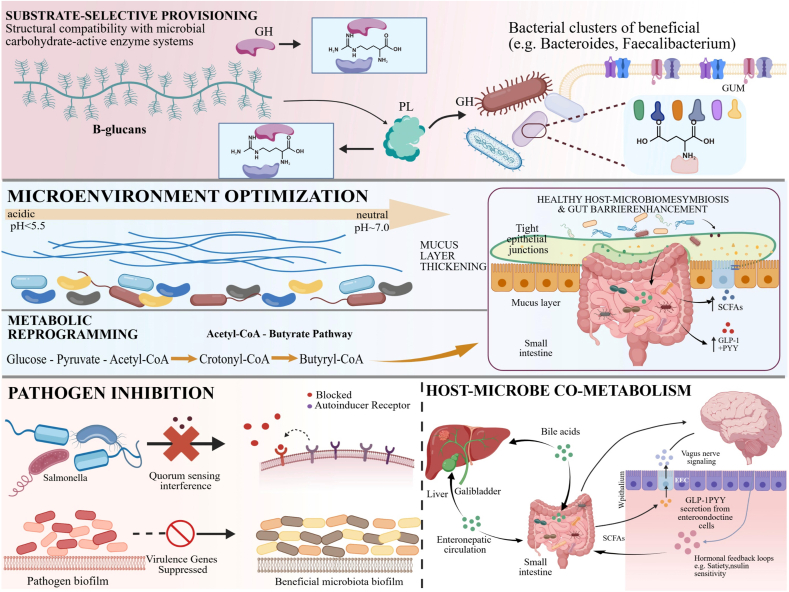
Mechanism of action of engineered polysaccharides in targeted regulation of SCFA-producing microbiota This figure depicts the core mechanisms of engineered food-derived polysaccharides in regulating SCFA-producing microbiota, including substrate-selective provisioning via CAZymes (GH/PL) for specific bacterial utilization, microenvironment optimization by SCFAs to lower intestinal pH, thicken mucus and enhance gut barrier integrity, metabolic reprogramming to generate butyrate and stimulate GLP-1/PYY secretion, pathogen inhibition through quorum sensing interference, and host-microbe co-metabolism linking gut, liver and brain via signaling pathways.

Engineered food-derived polysaccharides include inulin, β-glucan, pectin, and arabinose oligosaccharides. Their targeted regulation of SCFA-producing bacterial communities does not rely on the macro-level selective supply of substrates, but rather on precise recognition, transport, and metabolic regulation at the molecular level of polysaccharide-bacteria interactions. This is the core characteristic that distinguishes these polysaccharides from traditional broad-spectrum prebiotics. The structural characteristics of these indigestible dietary fibers, including glycosidic bond types, branching degrees, and modifying groups, significantly affect their ability to be preferentially utilized by specific gut microbiota. This process is initiated by the substrate-specific recognition of CAZymes (CAZac: an activity descriptor for). Glycoside hydrolases (GHs) and polysaccharide lyases (PLs) are the core enzyme classes mediating the initial degradation of polysaccharides. Both enzyme classes exhibit strict substrate and glycosidic bond recognition specificity. Through targeted engineering approaches, such as debranching to reduce steric hindrance and chemical modification to introduce ferulic acid, the spatial compatibility between the polysaccharide backbone and the CAZymes catalytic pocket can be significantly enhanced, thereby improving the strain specificity and enzymatic efficiency of substrate degradation (Kmezik et al., 2020, 2021). Fig. 6 summarizes the four core mechanisms underlying the targeted regulation of SCFA-producing microbiota by engineered food-derived polysaccharides, covering substrate-selective provisioning, microenvironment optimization, metabolic reprogramming, pathogen inhibition, and host-microbe co-metabolism.

During the intracellular uptake of hydrolysis products, the competitive accumulation mechanism mediated by solute-binding proteins (SBPs) further enhances the microbial community targeting of engineered polysaccharides. Transmembrane transport of oligosaccharide fragments by gut bacteria is highly dependent on periplasmic SBPs within the ABC transport system. The three-dimensional conformation of SBPs and the amino acid arrangement of the substrate-binding pocket determine the selective recognition of specific oligosaccharide motifs; only oligosaccharides that perfectly match the spatial conformation and charge distribution can achieve high-affinity binding (Theilmann et al., 2019). Through engineering approaches such as enzymatic hydrolysis and chemical modification, the glycosidic bond configuration and chain length distribution of polysaccharide degradation products can be precisely regulated. This ensures that the degradation products perfectly match the binding sites of SBPs in target SCFA-producing bacterial strains, thereby achieving the directed enrichment of functional bacteria in intestinal carbon source competition while simultaneously excluding non-target strains and pathogenic bacteria lacking the corresponding transport systems.

These molecular recognition and transport processes collectively drive the directed remodeling of gut microbiota composition and metabolic networks. Fructooligosaccharides (FOS) produced by the hydrolysis of inulin by GH32 family enzymes can be efficiently taken up by Bifidobacterium and metabolized into acetate and lactate (Xue et al., 2025a; Li et al., 2025c), whereas xylooligosaccharides derived from xylans preferentially stimulate the proliferation of butyrate-producing bacteria such as ***Ruminococcaceae*** and *Bacteroides* (Function-based classification of carbohydrate). These probiotic effects are constrained by the characteristics of the host's baseline microbiota. **For** example, arabinose-based oligosaccharides exert stable functions only in individuals with high Prevotella abundance, a phenomenon closely linked to the synergistic degradation by the PULs system specific to Bacteroidetes that rely on such polysaccharides (Hou et al., 2021). Engineered polysaccharides can further amplify the synthetic effects of SCFAs by directionally activating strain cross-feeding networks. Primary degraders such as Bacteroides and Bifidobacterium convert complex polysaccharides into intermediate metabolites like lactic acid and succinic acid. These intermediates are subsequently captured by secondary SCFA-producing bacteria and converted into final products such as butyrate and propionate. By using engineered polysaccharides, such as highly branched inulin, to directionally regulate the production of intermediate metabolites, precise control over the yield and composition of intestinal SCFAs can be achieved (Rios-Covian et al., 2015; Moens et al., 2016; Prabhu et al., 2012).

Concurrently, engineered polysaccharides can create a stable colonization environment for SCFA-producing bacteria through multiple mechanisms. SCFAs produced by polysaccharide fermentation lower local intestinal pH, thereby inhibiting the proliferation of pathogenic bacteria. Among these, butyrate, the preferred energy source for colonic epithelial cells, upregulates the expression of tight junction proteins to maintain intestinal barrier integrity. Furthermore, engineered polysaccharides can reduce the displacement risk of SCFA-producing microbiota by pathogenic bacteria through mechanisms such as nutritional competition and occupation of epithelial adhesion sites. Ultimately, they facilitate systemic health benefits from microbiota-host interactions in animal models (Xue et al., 2025b; Multi-omics analysis provides insights into; Structural and functional characterisation of a stable).

### 4.5 Precision modification toolkit of food-derived polysaccharides targeting SCFA-producing microbiota: modular design, structure-activity relationship and neurological disorder-oriented functional verification

Based on the molecular design principles of precise prebiotics, the dual molecular screening barrier theory of polysaccharide-microbiota interactions, and the systematic engineered modification technology system summarized above, this study proposes a referable precision modification toolkit of food-derived polysaccharides for neurological disorders. This toolkit integrates the basic biological mechanisms of gut microbiota-polysaccharide interactions into an operable, predictable and standardized research and development reference system. It provides potential solutions to three major challenges in clinical translation, including insufficient targeting of target strains, uncontrollable SCFA metabolic effects, and large individual response differences caused by heterogeneous baseline microbiota of patients. Compared with the existing general models mostly focusing on hierarchical regulation of fiber structure, this toolkit further integrates site-specific structural modification, safety control and functional verification adapted to neurological disorders, and is more suitable for potential application scenarios of precise microbiota intervention in CNS diseases.

This toolkit is based on dual molecular screening barriers and divided into four functional modules that can be independently invoked or used in combination. The molecular skeleton editing module realizes preliminary targeted screening of utilizable microbiota at phylum and genus levels. The functional group site-specific modification module achieves precise targeted regulation at species and strain levels. The delivery performance optimization module adapts to the gastrointestinal pathological characteristics of patients with neurological disorders, such as abnormal gastrointestinal motility and disordered digestive enzyme secretion, to ensure the colonic targeted delivery efficiency of polysaccharides. The safety risk control module establishes a complete safety evaluation standard for long-term intervention in neurological disorders, filling a key gap in the existing system.

To verify the practical application value and operability of the toolkit, Alzheimer's disease (AD) intervention was taken as an example. Traditional prebiotics have limitations in AD intervention, such as insufficient targeting, uncontrollable butyrate synthesis, and large individual differences. This study takes the characteristic decrease of *Faecalibacterium prausnitzii* abundance and insufficient intestinal butyrate in AD patients as precise targets, and completes the whole-process directional design of modified *Poria cocos* β-glucan through the four modules of the toolkit. Multi-system verification results show that the modified polysaccharide can achieve directional enrichment of *Faecalibacterium prausnitzii* at the single-strain level without non-specific microbiota disturbance caused by traditional prebiotics. In the in vitro fermentation model of feces from AD patients, it can alleviate the limitation of heterogeneous baseline microbiota of hosts and stably increase the proportion of butyrate in total SCFA synthesis (Lai et al., 2025a). In AD model mice, it precisely targets colonic butyrate-producing microbiota and simultaneously achieves dose-dependent improvement of cognitive function, verifying that this toolkit breaks through the bottlenecks of traditional prebiotic research and development (Song et al., 2025b).

Meanwhile, the toolkit is equipped with a standardized verification system exclusive to neurological disorders. On the basis of conventional detection of microbiota structure, SCFA levels and intestinal barrier function, it supplements the detection of gut-brain axis effects, evaluation of population individual heterogeneity, and safety evaluation of interactions with neurotherapeutic drugs, so as to meet the needs of clinical intervention for neurological disorders.

## 5 Core neuroprotective pathways of engineered food-derived polysaccharides via the microbiota-gut-brain axis

**Fig. 7:**
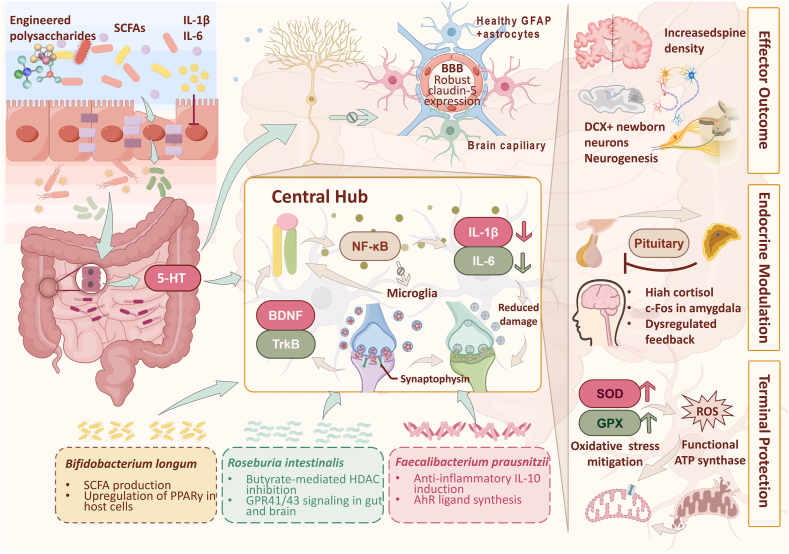
Neuroprotective effects of engineered polysaccharides exerted via the MGB axis This figure illustrates the neuroprotective effects of engineered food-derived polysaccharides via the microbiota-gut-brain axis (MGB axis). Engineered polysaccharides target SCFA-producing microbiota to boost SCFA production, which modulates inflammatory signaling, neuroplasticity, gut-brain barrier integrity, endocrine function, and oxidative stress, ultimately promoting neurogenesis and neuroprotection.

Precision prebiotics are essentially engineered food-derived polysaccharides with tailor-made molecular structures. They overcome key clinical limitations of conventional prebiotics, including poor targeting specificity, weak strain selectivity, and high inter-individual response heterogeneity. Through predefined structural modifications, these compounds precisely match the substrate preferences of target SCFA-producing microbiota. They achieve strain-level directional enrichment and controllable synthesis of SCFA subtypes. These modifications further alleviate disease-specific microbial dysbiosis and metabolic disorders in neurological conditions. Ultimately, precision prebiotics exert targeted neuroprotective potential through four major pathological pathways of the MGB axis (Fig. 7).

In contrast to the non-specific microbial regulatory effects of conventional prebiotics, the neuroprotective effects of precision prebiotics follow clear structure-target-effect relationships. These compounds adapt to the pathological features and intervention requirements of different neurological disorders. They thus provide personalized precision nutritional intervention strategies for diseases associated with nerve injury. This chapter systematically summarizes representative in vitro and in vivo studies on the neuroprotective effects of precision prebiotics with customized structures via the MGB axis. Table 3 lists the polysaccharide modification methods, targeted strains, regulatory mechanisms, and functional outcomes of these studies. The table clarifies the correspondence between structural customization and solutions to clinical challenges.

**Table 3:** Polysaccharides in neurological disorder regulation.

Serial No.	Source of Polysaccharides	Model System	Intervention Method	Main Mechanisms	Behavioral/Pathological Improvement Effects	References
1	Fucoidan from *Saccharina japonica* (LJF-2)	D-galactose-induced senescent mice	Oral administration for 8 weeks	Regulates tryptophan metabolism to increase the synthesis of 5-hydroxytryptamine. It remodels the intestinal microbiota and strengthens the intestinal barrier. It also activates the CREB signaling pathway in the brain	Improves spatial learning and memory ability and inhibits neuroinflammation as well as oxidative stress	Wei et al. (2021)
2	Astragalus polysaccharide (APS)	Mice with systemic lupus erythematosus model	Intragastric administration for 14 days as therapeutic intervention	Regulates the proportion of intestinal microbiota by increasing the abundance of *Bifidobacterium* and *Lactobacillus* and decreasing the abundance of *Escherichia coli* and *Enterococcus faecalis*. It also reduces the levels of inflammatory factors in serum	Improves the alternation rate in Y-maze test and decreases the neurological deficit score	Sun et al. (2024b)
3	*Ziziphus jujuba* seed polysaccharide (ZJP)	Chronic unpredictable mild stress-induced depressive mice	Intragastric administration for 44 days	Reduces the abundance of butyrate-producing bacteria such as *Alistipes* and *Lachnospiraceae*. It downregulates short-chain fatty acid metabolic genes and decreases the butyrate level in feces	Alleviates depressive-like behaviors and increases the level of 5-hydroxytryptamine in the hippocampus. It also reduces the levels of proinflammatory factors	(Lycium Barbarum Polysaccharides Improves Cognitive)
4	*Lycium barbarum* polysaccharide (LBP)	APP/PS1 transgenic mice with Alzheimer's disease	Oral administration	Regulates the structure of intestinal microbiota and increases the levels of short-chain fatty acids. It repairs the intestinal barrier and inhibits neuroinflammation. It also regulates the expression of APP cleaving enzymes	Improves the performance in water maze test and reduces amyloid-β deposition. It also inhibits the activation of microglia	Jiang et al. (2025b)
5	*Juglans regia* kernel polysaccharide (WKP)	Mice with neurotoxicity induced by ochratoxin A	Co-administration via intragastric route for 7 days	Enriches short-chain fatty acid-producing bacteria such as *Lactobacillus* and restores the levels of acetate and propionate. It repairs the intestinal barrier and regulates the expression of genes related to mitochondria and synapses	Improves exploratory behaviors and alleviates depressive-like immobility. It also reduces the loss of hippocampal neurons	Qiu et al. (2024)
6	*Angelica sinensis* polysaccharide (ASP)	Mice with middle cerebral artery occlusion and reperfusion injury	Intragastric administration for 7 days prior to model establishment	Regulates the α-diversity of intestinal microbiota and inhibits the expression of IL-1β and IL-6. It increases the level of IL-10 and alleviates oxidative stress by elevating the activities of superoxide dismutase and the content of glutathione while reducing the level of malondialdehyde	Decreases the neurological deficit score and reduces the volume of cerebral infarction	(Radix Hedysari Polysaccharides Modulate the)
7	*Polygonatum odoratum* polysaccharide (PSP)	SAMP8 senescence-accelerated mice	Oral administration for 12 weeks	Regulates serum metabolites and enhances neuronal autophagy. It repairs the intestinal barrier and improves the ultrastructure of mitochondria	Improves cognitive function and reduces the damage of hippocampal neurons	Zhang et al. (2022a)
8	*Polygonatum odoratum* polysaccharide extract (PSPE)	*Caenorhabditis elegans* with neurotoxicity induced by fumonisin B1	Pretreatment with concentrations ranging from 20 to 200 μg/mL	Reduces the level of reactive oxygen species and enhances the activity of antioxidant enzymes. It improves mitochondrial function by increasing the production of ATP and mitochondrial membrane potential and regulates mitochondrial dynamics	Alleviates motor deficits and reduces neuronal damage	Xu et al. (2023)
9	*Dendrobium officinale* polysaccharide (DOP)	D-galactose-induced senescent mice	Oral intervention	Remodels the intestinal microbiota by decreasing the Firmicutes/Bacteroidetes ratio and increasing the abundance of *Lactobacillus*. It also alleviates oxidative damage of glial cells	Inhibits the activation of glial cells and elevates the activity of antioxidant enzymes	Ren et al. (2024)
10	*Inonotus obliquus* polysaccharide (ORP-1)	Naturally senescent mice	Oral intervention	Increases the abundance of beneficial bacteria such as *Dubosiella* and bacteria of the order *Clostridiales*. It repairs tight junctions and the expression of mucin 2 and inhibits the excessive activation of microglia	Improves memory and synaptic plasticity and alleviates neuroinflammation	Liu et al. (2022b)
11	Pleurotus umbrella polysaccharide (PSP2-1)	D-galactose-induced senescent mice	Intraperitoneal injection	Inhibits the MAPK signaling pathway including JNK p38 and Erk and regulates the expression of Bcl-2 and Bax. It reduces the activation of caspase-3 and decreases the accumulation of reactive oxygen species	Improves learning and memory ability and alleviates oxidative stress-induced damage	Chen et al. (2021)
12	Polysaccharide from pepper and Chinese prickly ash peel (PPZM)	*PC12* cell injury induced by hydrogen peroxide	In vitro pretreatment	Inhibits the release of lactate dehydrogenase and malondialdehyde and elevates the activity of superoxide dismutase. It regulates the apoptotic pathway by decreasing the expression of Bax and Caspase-3 and increasing the expression of Bcl-2	Improves cell survival rate and inhibits abnormal apoptosis	Liu et al., 2025c
13	*Gastrodia elata* polysaccharide (GEP-1/GEP-2)	*HT-22* cell injury induced by copper exposure	In vitro intervention	Inhibits oxidative stress and inflammation. The high-molecular-weight GEP-2 exerts a better effect which is attributed to the differences in its branching degree and conformation	Alleviates cell injury and GEP-2 shows a superior effect compared with GEP-1	(Extraction)
14	*Gastrodia elata* polysaccharide	Review involving multiple models	Not applicable	Exerts antioxidant anti-inflammatory immunoregulatory and neuroprotective effects. A clear structure-activity relationship has been identified	Possesses potential application in the prevention and treatment of neurodegenerative diseases	Yun et al. (2022)
15	*Lycium ruthenicum* polysaccharide (LRPS)	Review involving in vitro and in vivo studies	Not applicable	Exerts antioxidant anti-radiation neuroprotective and prebiotic activities. The biological activity is affected by its structural characteristics	Has the potential to be developed into functional foods and pharmaceuticals	(Advances in the Preparation; Liu et al., 2022c)
16	Selenylated rose hip polysaccharide (Se-RLFPs)	*SH-SY5Y* cell injury induced by hydrogen peroxide	In vitro treatment	Activates the Nrf2/HO-1 signaling pathway and elevates the total antioxidant capacity and the activity of superoxide dismutase. It also decreases the level of malondialdehyde	Protects neurons from oxidative damage	Tan et al. (2025)
17	Selenylated *Physalis peruviana* polysaccharide (Se-PPFP)	In vitro cell model	Testing after chemical modification	Selenium modification enhances its bioavailability and improves its thermal stability. It also exerts stronger antioxidant and neuroprotective effects	Exerts a more significant protective effect compared with unmodified PPFP	(Dai et al.a)
18	*Pleurotus ostreatus* oligosaccharide derivative (Compound C)	*PC12* cell injury induced by hydrogen peroxide	In vitro pretreatment	Regulates the Bcl-2/BAX and PI3K/Akt signaling pathways and shows no cytotoxicity	Significantly improves cell survival rate and exerts the optimal therapeutic effect	(Dai et al.b)
19	Low-molecular-weight galactomannan from *Sesbania cannabina*	Zebrafish (*Danio rerio*) neuroinjury induced by bisphenol AF	In vivo intervention	Scavenges free radicals including DPPH and superoxide anion and reduces the level of reactive oxygen species in vivo. It also protects basic growth and motor functions	The recovery rate of motor function reaches 193.7 percent and the growth recovery rate reaches 62.5 percent	Wu et al. (2025b)
20	*Auricularia auricula* polysaccharide (TP)	High-fat diet-induced anxious mice	Intragastric administration at a dose of 400 mg/kg for 12 weeks	Repairs the intestinal barrier and inhibits the activation of microglia. It regulates the levels of 5-hydroxytryptamine and norepinephrine in the hippocampus and remodels the intestinal microbiota by decreasing the abundance of Proteobacteria. Fecal microbiota transplantation verifies the causal relationship	Improves anxiety-like behaviors and the protective effect can be transferred via fecal microbiota transplantation	(Li et al.)
21	Polysaccharide from *Citrus aurantium* and *Citrus reticulata* peel (PCRCPI)	High-fat diet-induced depressive mice	Intragastric intervention	Enriches the abundance of *Lactobacillus* and elevates the level of 2-arachidonoylglycerol. It also activates the endocannabinoid signaling pathway in the hippocampus	Improves behavioral performance and elevates the levels of neurotransmitters. Fecal microbiota transplantation verifies the microbiota-mediated mechanism	Zhang et al. (2026)
22	*Hovenia dulcis* petiole polysaccharide (HDP-2w)	Alcohol-induced neuroinjury mice	Intragastric administration for 14 days	Upregulates the expression of Claudin-1 and ZO-1 in the intestine and hippocampus and enriches the abundance of *Lactobacillus*. It also regulates cerebral glutamate metabolism by increasing the levels of glutathione and glycine	Improves spatial memory and decreases blood alcohol concentration as well as DNA damage	(Fan et al.)
23	Barley polysaccharide	Naturally senescent mice with high-fat diet and chronic stress	Long-term dietary supplementation	Regulates the metabolism of bile acids and lysine and enriches the abundance of *Muribaculaceae* and *Lachnospiraceae*. It increases the level of butyrate and repairs the intestinal barrier	Improves long-term memory and decreases the level of corticosterone. It also alleviates liver damage	(Fu et al.)
24	*Anoectochilus roxburghii* polysaccharide (ARPs)	High-fat diet-induced cognitive impairment mice	Dietary supplementation for 14 weeks	Repairs intestinal tight junction proteins and regulates the intestinal microbiota by decreasing the abundance of *Parabacteroides*. It also reduces the levels of serum inflammatory factors blood glucose and cholesterol	Improves spatial learning and memory ability and decreases the phosphorylation of Tau protein	(Zhang et al.)
25	*Physalis alkekengi* polysaccharide (SCP-1)	AD-like mice induced by D-galactose and aluminum trichloride	Oral intervention	Promotes the growth of butyrate-producing bacteria such as *Intestinaimonas* and inhibits the growth of proinflammatory bacteria such as *Escherichia* and *Shigella*. It also inhibits the TLR4/NF-κB signaling pathway	Improves learning and memory ability and reduces the production of amyloid-β. It also regulates the levels of neurotransmitters	(Kumari et al.)
26	Lactobacillus exopolysaccharide (EPSRam12)	D-galactose-induced brain injury mice	Intragastric intervention	Increases the activities of antioxidant enzymes including superoxide dismutase catalase and glutathione peroxidase and decreases the levels of malondialdehyde nitric oxide and tumor necrosis factor-alpha. It enriches short-chain fatty acid-producing bacteria and repairs the intestinal mucosal barrier	Alleviates oxidative stress and inflammation in the brain and improves the diversity of intestinal microbiota	(Radix Hedysari Polysaccharides Modulate the)
27	*Hemerocallis citrina* root polysaccharide (RHP)	*HT22* cell injury induced by amyloid-β and SAMP8 mice	Cell pretreatment and oral administration in mice	Reduces the levels of reactive oxygen species and mitochondrial superoxide and maintains the mitochondrial membrane potential. It regulates the serum metabolome and enhances neuronal autophagy	Exerts antioxidant effects on cells and improves cognitive function in mice. It also protects the mitochondrial structure	Choe (2025c)
28	Dietary fiber/short-chain fatty acids	Review involving multiple neurodegenerative disease models	Not applicable	Regulates immune metabolic and barrier functions via the microbiota-gut-brain axis and short-chain fatty acids act as key messengers in this process	Prevents the progression of diseases such as Alzheimer's disease and Parkinson's disease	(Song et al.)
29	Review of natural polysaccharides	Multiple models of neurological diseases	Not applicable	Inhibits neuroinflammation oxidative stress and protein misfolding. It maintains the integrity of the intestinal and blood-brain barriers and regulates the intestinal microbiota as well as neurotransmitters	Possesses the potential for multi-target intervention and further clinical verification is required	(Yu et al.)
30	Review of plant polysaccharides	Multiple models of central nervous system diseases	Not applicable	Inhibits neuroinflammation apoptosis and autophagy disorder and regulates the homeostasis of intestinal microbiota. The main challenges include structural heterogeneity low bioavailability and poor central nervous system penetration	Proposes strategies of nano-delivery and chemical modification for polysaccharide development	Zhang et al. (2022b)
31	Polysaccharides in Alzheimer's disease and Parkinson's disease	Review focusing on the microbiota-gut-brain axis	Not applicable	Regulates microbial metabolism to affect neuroinflammation and protein aggregation. Some related studies have entered the clinical trial stage	Provides a new therapeutic framework for neurodegenerative diseases	Wang et al. (2022b)
32	Polysaccharides regulating intestinal microbiota in cognitive impairment and depression	Review involving preliminary animal and human studies	Not applicable	Maintains the balance of intestinal microbiota to improve the intestinal barrier which in turn affects brain function via the microbiota-gut-brain axis. The mechanism in depression is investigated in particular	Proposes a new microbiota-gut-brain axis-based intervention strategy for depression	(Wang et al.)
33	Intestinal microbiota-colon cell interaction	High-fat diet-induced cognitive impairment mice	Supplementation with *Eucommia ulmoides* bark polysaccharide	Reconstructs the microbiota-host interaction and restores the levels of short-chain fatty acids. It also alleviates neuroinflammation	Reverses diet-induced cognitive impairment	(Advances in the Preparation)
34	Short-chain fatty acids (SCFAs)	Multiple models of central nervous system diseases	Not applicable	Affects brain function via neuroendocrine immune and metabolic signaling pathways and participates in the pathogenesis of neurodegenerative cerebrovascular epileptic and mood disorders	Acts as core mediators of the microbiota-gut-brain axis and the underlying mechanisms need further in-depth research	Guo et al. (2022a)
35	Sodium butyrate (NaB)	MPTP-induced Parkinson's disease mice	Intragastric administration for 21 days	Repairs intestinal microbiota dysbiosis and inhibits the intestinal and cerebral TLR4/MyD88/NF-κB signaling pathways. It reduces the levels of proinflammatory factors and protects dopaminergic neurons	Improves motor function and increases the levels of neurotransmitters in the striatum	Guo et al. (2022b)
36	Lactobacillus LA	MPTP-induced Parkinson's disease mice	Intragastric intervention	Repairs the intestinal microbiota and enhances the GLP-1/FFAR2/FFAR3 signaling pathway. It improves the intestinal barrier and upregulates the expression of GLP-1 receptors in the brain	Improves motor function and increases the number of tyrosine hydroxylase-positive neurons. It also inhibits neuroinflammation	(Guo et al.)
37	Fecal microbiota transplantation (FMT)	Rhodamine-induced Parkinson's disease mice	Transplantation of healthy intestinal microbiota	Reverses intestinal microbiota dysbiosis by decreasing the abundance of *Akkermansia* and *Desulfovibrio*. It repairs the intestinal barrier and inhibits systemic and cerebral inflammation as well as protects the blood-brain barrier	Improves gastrointestinal and motor functions and reduces the damage of substantia nigra neurons	(Fecal Microbiota Transplantation Protects Rotenone)
38	Polysaccharide-mediated multi-organ axis network	Models of systemic diseases	Not applicable	Regulates multiple axes including the microbiota-gut-brain microbiota-gut-lung and microbiota-gut-liver axes. Chemical modifications such as selenylation and sulfation enhance the specificity of polysaccharides	Proposes a new generation of polysaccharide therapeutic strategies combining multi-omics analysis and precise chemical modification	Zhu et al. (2020b)
39	Amphibian aquatic microorganisms	Tadpoles of the North American leopard frog (*Lithobates pipiens*)	Rearing in natural water versus sterile water	Aquatic microorganisms shape the intestinal microbiota which in turn affects behavioral responses brain weight and brain morphology	Supports the existence of the microbiota-gut-brain axis in non-mammalian species and possesses ecological protection significance	Emerson and Woodley (2024)
40	Curcumin (reference for control)	DSS-induced anxious mice	Intragastric administration with verification via fecal microbiota transplantation	Regulates specific intestinal microbiota such as *Muribaculaceae* to increase the level of phosphatidylcholine in the prefrontal lobe which in turn alleviates anxiety	Fecal microbiota transplantation confirms the microbiota-mediated anxiolytic effect and the mechanism involves lipid metabolism	Zhang et al. (2022c)

### 5.1 Targeted blockade of immune-inflammatory pathological pathways and neuroprotective mechanisms of engineered food-derived polysaccharides

Conventional prebiotics suffer from uncontrolled structures, leading to weak strain targeting, fluctuating butyrate production, and marked heterogeneity in anti-inflammatory effects among individuals. Precision prebiotics employ site-specific structural modifications to match the substrate preferences of anti-inflammatory SCFA-producing microbiota such as *Bacteroides* and *Lactobacillus*. These modifications enable strain-level directional enrichment and stably elevate intestinal butyrate to the effective anti-inflammatory range of 0.5–2 mmol/L. This approach resolves the unstable anti-inflammatory effects and large inter-individual response variations of conventional prebiotics.

SCFAs, particularly butyrate generated by microbial fermentation, act as key effector molecules. They serve as histone deacetylase (HDAC) inhibitors to regulate the expression of inflammation-related genes. They also mediate inflammatory signaling through **G protein-coupled receptors** and precisely inhibit the release of pro-inflammatory factors. These actions alleviate systemic and neuroinflammation at the initial stage of inflammatory responses and provide novel regulatory strategies for targeted intervention of brain-related diseases (Choe, 2025). The inflammatory homeostasis regulated by engineered polysaccharides bidirectionally modulates the integrity of the intestinal barrier and blood-brain barrier, neuroendocrine homeostasis, and neuronal epigenetic modification status. This forms the molecular basis for multi-pathway synergistic neuroprotection.

Targeted engineering modification of polysaccharides affects the selective enrichment of SCFA-producing microbiota. It further determines the disease-specific targets and potency of anti-inflammatory effects. Fucoidan-cerium nanocomposites are oral engineered polysaccharide nanozymes. They target inflamed colon tissues through electrostatic interactions and exert dual functions of anti-inflammation and microbial metabolic regulation. In mouse models of ulcerative colitis complicated with mental disorders, this nanocomposite directionally enriches SCFA-producing microbiota including *Lactobacillus* and *Faecalibacterium prausnitzii*. It elevates intestinal butyrate levels, repairs the intestinal barrier, and suppresses excessive activation of microglia and astrocytes in the hippocampus. The compound also reduces the abundance of pathogenic bacteria such as *Escherichia coli* and *Shigella* to reshape intestinal microbial homeostasis. These effects ultimately alleviate depressive-like behaviors in mice and support engineered polysaccharide design for intervention of inflammation-related neuropsychiatric disorders (Wei et al., 2026). Sulfation modification introduces negatively charged sulfate groups into polysaccharide chains. This process changes the charge density and spatial conformation of polysaccharides. The modified polysaccharide structures bind with high affinity to carbohydrate-binding modules on the surface of *Bacteroides*. This enhances the selective enrichment efficiency of these SCFA-producing microbiota and provides microbial support for subsequent neuroinflammation regulation.

Ultrasonically modified polygonatum polysaccharides selectively enrich *Lactobacillus* and *Faecalibacterium prausnitzii*. Degradation of high-molecular-weight components, optimization of branching degree, and improved water solubility contribute to the high selectivity of these modified polysaccharides. They significantly increase intestinal butyrate content. In animal models, butyrate maintains intestinal barrier integrity by activating intestinal free fatty acid receptor 2/3 (FFAR2/3). Butyrate also crosses the blood-brain barrier to enter the CNS. It inhibits excessive activation of hippocampal microglia through the HDAC/NF-κB pathway and reduces interleukin-1β release. These changes ultimately improve cognitive deficits and depressive-like behaviors in model mice (Polygonatum sibiricum polysaccharide prevents depression; Liu et al., 2026b; Mishra et al., 2021). Laminarin polysaccharides with site-specific sulfation modification show optimized charge density and spatial conformation. These modifications enhance the enrichment efficiency of SCFA-producing *Bacteroides* strains. They adjust intestinal SCFAs including butyrate to near physiological effective concentrations (Modulation effect of sulfated polysaccharide; Han et al., 2021; Beyond the Gut; Xing et al., 2023). The modified polysaccharides further suppress neuroinflammation in the substantia nigra of PD model mice. They protect dopaminergic neurons and alleviate motor dysfunction in mice through peripheral FFAR3-mediated gut-brain signaling and central HDAC signaling axes. Sulfation modification regulates polysaccharide charge density and spatial conformation by directional introduction of negatively charged sulfate groups. This promotes efficient binding to carbohydrate-binding modules on *Bacteroides* surfaces and enables precise enrichment of SCFA-producing microbiota. The modification establishes a robust microecological foundation for neuroinflammation regulation.

Recent studies have questioned the universal applicability of this mechanism across different neurodegenerative disorders. For example, patients with PD complicated by cognitive impairment show reduced SCFA-producing microbiota and increased pathogenic bacteria. However, cognitive impairment itself does not significantly alter intestinal microbial structure. This finding indicates that the pathological progression of PD exerts stronger effects on microbiota than cognitive impairment. It also suggests that the efficacy of SCFA intervention is limited by disease stage and phenotypic heterogeneity (Secondary analysis reveals gut microbiota). In addition, knockout of key complement system components significantly alters intestinal microbial composition in AD model mice. No such changes are observed in wild-type mice. This result demonstrates that host immune and genetic backgrounds reshape microbial structure. These backgrounds further interfere with the anti-inflammatory pathways mediated by exogenous polysaccharides through the microbiota-SCFA axis (Petrisko et al., 2023).

Current studies have confirmed associations among polysaccharide intervention, changes in microbial abundance, and improved inflammatory phenotypes. Most of these correlational studies remain at the phenotypic validation level. The molecular mechanisms of structural interactions, substrate competition, and synergistic effects among polysaccharide components remain unclear. Strain-level studies further reveal that exopolysaccharides secreted by different strains of the same bacterial species exert opposite immune outcomes. These compounds either activate or inhibit the TLR4/NF-κB pathway. This means that engineered polysaccharides may aggravate rather than alleviate neuroinflammation if they enrich pro-inflammatory strains (Laplanche et al., 2025). Other studies show that some probiotic-derived polysaccharides directly bind competitively to the CD14 receptor. These polysaccharides block the lipopolysaccharide-TLR4 signaling pathway and exert anti-inflammatory effects independent of SCFAs. These findings highlight the necessity of dissecting multi-level mechanisms of inflammation regulation by polysaccharides and microbiota (Competitive Inhibition of LPS Binding).

### 5.2 Targeted repair of gut-brain dual barrier damage and pathological signal blocking mechanisms of engineered food-derived polysaccharides

Engineered food-derived polysaccharides can target and enrich SCFA-producing microbiota through directional structural modification to elevate in situ butyrate levels in the colon. Such modification alleviates damage to the impaired epithelial barrier and reduces peripheral and central inflammatory responses triggered by endotoxin entry into the bloodstream. Butyrate serves as the primary energy substrate for colonic epithelial cells. It upregulates the expression of tight junction proteins including occludin, claudin-1, claudin-3 and ZO-1 by activating the hypoxia-inducible factor 1α (HIF-1α) pathway. This process alleviates the leaky gut phenotype and generates upstream and downstream synergistic protective effects with immune-inflammatory pathways. Recent studies have further confirmed that chemically modified polysaccharides repair local barriers and exert neuroprotective effects through the microbiota-gut-brain axis. For instance, Tian et al. (2024) reported that polysaccharide-based delivery systems such as butylated starch enable targeted delivery and controlled release of butyrate in the colon. These systems effectively repair the impaired epithelial barrier and block peripheral and central inflammatory responses caused by endotoxin translocation. The neuroprotective effects are dependent on the restoration of intestinal barrier function (Competitive Inhibition of LPS Binding). This mechanism has been preliminarily verified in animal models. Partially hydrolyzed guar gum (PHGG) is prepared by selective hydrolysis of glycosidic bonds in the main chain of natural guar gum with endo-β-D-mannanase. The hydrolysis process reduces molecular weight and solution viscosity and improves water solubility and gastrointestinal stability. The structural and fermentation properties of polysaccharides are fully preserved during this process. PHGG can be specifically fermented by colonic microbiota to significantly increase the levels of SCFAs including butyrate. It upregulates the expression of tight junction proteins such as occludin and ZO-1 to restore the integrity of the intestinal mucosal barrier. PHGG also inhibits the activation of the NLRP3 inflammasome and blocks the cascade transmission of peripheral inflammatory signals to the central nervous system. Animal experiments have confirmed that PHGG significantly alleviates neurological deficits and reduces cortical neuronal loss in mice with traumatic brain injury. These results demonstrate the neuroprotective potential of PHGG mediated by the microbiota-gut-brain axis (Huang et al., 2025). The intestinal epithelial barrier acts as a common upstream node for immune inflammation, endocrine metabolism and direct neural communication pathways. Barrier repair suppresses peripheral inflammatory release, promotes glucagon-like peptide-1 (GLP-1) secretion and maintains normal vagus nerve transmission. This creates a positive synergistic cycle with the three major pathways. Current studies have not clarified the precise structure-activity relationships among engineered polysaccharide structures, microbial targeting and barrier repair effects. The dominant role of barrier repair has not been verified by rescue experiments. These issues represent key bottlenecks for future research and development.

### 5.3 Targeted correction and homeostatic restoration mechanisms of engineered food-derived polysaccharides on neuroendocrine disorder pathways

Conventional prebiotics cannot specifically enrich SCFA-producing microbiota that activate intestinal L cells due to uncontrolled branch chain structures. This leads to non-specific regulatory effects on GLP-1 and peptide YY (PYY) secretion and large inter-individual variations. Effective improvement of neuroendocrine homeostasis is therefore difficult to achieve**.** Precision prebiotics can precisely match the substrate preferences of butyrate-producing and propionate-producing bacteria in L-cell-enriched regions through directional customization of branch chain topology and monosaccharide composition. These prebiotics stably enhance intestinal butyrate and propionate synthesis and specifically activate GPR41/FFAR3 receptors on the surface of intestinal L cells. This promotes the controllable synthesis and secretion of GLP-1 and PYY and resolves the non-specific neuroendocrine regulation of conventional prebiotics (Huang et al., 2025).

Directional structural modification and engineered reconstruction of polysaccharides represent a core promising strategy to enhance their microbiota-targeting specificity, metabolic regulatory potency, and neuroprotective bioactivities. Sulfated and selenized modifications of kelp fucoidan LJF-2 reshape gut microbiota, reinforce intestinal barrier integrity, and shift tryptophan metabolism toward serotonin synthesis in cognitive impairment models. They exert neuroprotection via activating cerebral cAMP response element-binding protein (CREB) pathway in a gut microbiota-dependent manner validated by fecal microbiota transplantation (Wang et al., 2026). Metabolomics-guided modified corn silk polysaccharides (CSPs) modulate host-microbiota co-metabolic networks. They indirectly regulate cerebral energy homeostasis via SCFAs and bile acid-mediated gut-brain signaling to improve insulin sensitivity and alleviate neuroinflammation (Shen et al., 2024; Zhu et al., 2020a). Dextran-modified poly(lactic-co-glycolic acid) nanoparticles, an engineered polysaccharide delivery system, regulate intestinal immune microenvironment and blood-brain barrier permeability via SCFAs profile modulation to remotely govern cerebral energy metabolism and neuroimmune homeostasis through epigenetic mechanisms (Chen et al., 2020; Microbial Regulation of Host Physiology). Type V starch nano-helical structures reconstructed by enzymatic hydrolysis and high-pressure homogenization *Akkermansia muciniphila*. They repair the mucosal barrier, stimulate glucagon-like peptide-1 (GLP-1) secretion via farnesoid X receptor (FXR) and G protein-coupled bile acid receptor 1 (TGR5) axis, and inhibit hypothalamic inflammation to restore energy homeostasis through vagal afferent signaling (Chen et al., 2016). In summary, various engineered polysaccharides and their derivatives exert multi-dimensional metabolic regulation and neuroprotective effects by targeting the microbiota-gut-brain axis.

The in vivo functional effects of engineered polysaccharides primarily depend on their structural stability during digestion in the upper gastrointestinal tract. Lycium polysaccharides, whose stability has been verified through in vitro simulated digestion systems, can reach the colon intact and be metabolized by *Lactobacillus reuteri*. By increasing propionate levels in the intestine, they effectively enhance the GLP-1 secretion capacity of intestinal L cells, providing a stable functional foundation for metabolic regulation (Mannose-modified Prunus persica kernel protein). Excessively high structural stability is not always beneficial. Black fungus β-glucan can resist degradation in the upper gastrointestinal tract, excessive steric hindrance actually limits its contact efficiency with surface enzymes of the *Akkermansia muciniphila* microbiota, leading to delayed fermentation initiation. In contrast, moderately degraded products are more readily utilized by early-colonizing bacteria, triggering a cascade of fermentation reactions and ultimately resulting in higher total SCFA accumulation in the distal colon (Zeaxanthin Dipalmitate; Pratap et al., 2022). Therefore, engineered strategies with controllable and moderate instability are often superior to designs with absolute structural stability.

### 5.4 Targeted remodeling and signal restoration mechanisms of engineered food-derived polysaccharides on direct gut-brain neural communication pathways

Anxiety, depression, cognitive impairment, and other gut-related - Neuropsychiatric disorders associated with gut-brain axis dysfunction are consistently accompanied by abnormalities in bidirectional signaling between the gut and the central nervous system. The core therapeutic value of engineered food-derived polysaccharides lies in their ability to enhance SCFA levels by specifically modulating SCFA-producing microbiota, thereby repairing the impaired gut-brain signaling pathways under pathological conditions, restoring normal vagus nerve signaling function, and simultaneously maintaining the integrity of the blood-brain barrier. The effects of SCFAs on the central nervous system are not completely beneficial. Imbalanced SCFA proportions or excessive concentrations may exacerbate neuroinflammation and cognitive decline in neurodegenerative diseases or viral infections. This occurs through microglial activation and epigenetic disorder induction (Majumdar et al., 2023). In addition, the ability of SCFAs to cross the blood-brain barrier and their homeostatic concentrations in brain parenchyma remain controversial. It is unclear whether SCFAs independently mediate significant neural regulatory effects or act only as indirect mediators of systemic inflammatory responses (O'Riordan et al., 2022a).

The vagus nerve serves as the most direct signaling pathway linking the gut and the central nervous system. It acts as a carrier for SCFAs to exert central regulatory effects. Animal experimental results demonstrate that *Eucommia ulmoides* polysaccharide EFP-40 with a highly branched pectin structure significantly increases acetate and butyrate concentrations through in vitro fermentation, selectively promote the proliferation of *Bacteroides* and *Faecalibacterium prausnitzii*, and ultimately regulate central neural function by enhancing vagus nerve signal transmission (Zhang et al., 2024e). Wild morel polysaccharides purified by hot water extraction can increase the abundance of intestinal *Bacteroides* and elevate the total content of SCFAs. Its regulatory effect is not limited to basic vagus nerve activation. It may facilitate the projection of vagal afferent fibers to the hippocampus and amygdala. These two brain regions govern emotion and cognition, and the polysaccharide may thereby participate in modulating the pathological mechanisms of related diseases (Vagus Nerve as Modulator of). This pathway is easily impaired in chronic stress or intestinal inflammation models. Intestinal tryptophan metabolism shifts to the kynurenine pathway and inhibits serotonin synthesis. The promoting effect of SCFAs on serotonin secretion cannot effectively compensate for the overall neurotransmitter imbalance under such conditions (O'Riordan et al., 2022b).

Water-soluble extract MBP-2 derived from mung bean hull polysaccharides increases colon length and elevates intestinal SCFA concentrations in a dose-dependent manner, while enriching two major SCFA-producing microbial phyla including Firmicutes and Bacteroidetes. This extract presents a unique characteristic of activating the vagal nerve pathway and promoting intestinal serotonin release simultaneously. As an essential precursor of monoamine neurotransmitters, serotonin cannot penetrate the blood-brain barrier. It may exert biological effects by activating vagal nerve terminals in the intestinal mucosa, which further amplifies the central regulatory function of the vagus nerve and forms a synergistic regulatory interaction among intestinal microbiota, SCFAs, neurotransmitters and the vagus nerve (Xie et al., 2022). Partial hydrolysis modification is a key strategy to optimize the gut-brain regulatory effects of tamarind seed polysaccharides. Low-molecular-weight derivatives ETSP1 (176.68 kDa) and ETSP2 (34.34 kDa) are obtained by partial hydrolysis with endoglucanase. The total SCFA yield of these derivatives in vitro is slightly lower than that of natural polysaccharides. The derivatives significantly reduce the Firmicutes/Bacteroidetes ratio and achieve synergistically enhanced neural regulatory functions (Li et al., 2023). ETSP1 specifically enriches *Bacteroides vulgatus*, which is closely related to intestinal barrier integrity and synthesis of neuroactive metabolites. ETSP2 specifically promotes the proliferation of *Bacteroides xylanisolvens*. These results highlight that structural modification can achieve precise targeted regulation at the strain level. Animal experimental observations show that changes in gut microbiota are correlated with the improvement of neurological function. It is speculated that such engineered polysaccharides exert the beneficial functions of probiotics including *Bifidobacterium* and *Faecalibacterium prausnitzii* via the vagus nerve pathway, while inhibiting pathogenic bacteria such as *Escherichia coli* and *Shigella*, and ultimately mediate the regulation of brain function through the vagus nerve pathway. Acid-hydrolyzed laver polysaccharide PHP-D increases the abundance of *Lactobacillus* and SCFA levels. This polysaccharide repairs the intestinal mucosal barrier and indirectly regulates central neural function (Yu et al., 2023). Enzymatically hydrolyzed konjac glucomannans KGM-M-1/M-2 targeted action in the distal colon due to reduced molecular weight, promote the production of acetic, propionic, and butyric acids; their spatial distribution characteristics may further influence neural signaling pathways (Yin et al., 2020).

Most existing studies have ignored the regulatory effects of host genotype on SCFA responses. Studies have shown that Parkinson's disease patients carrying specific free fatty acid receptor 3 variants exhibit significantly weaker neuroprotective responses to fecal microbiota transplantation or SCFA supplementation than wild-type individuals. This indicates that universal intervention strategies for engineered polysaccharides urgently need to transform into genotype-adapted designs (Ni et al., 2025). Research limited to single in vitro model verification cannot explore the dose-response relationships of polysaccharides with different branching degrees and molecular weights on vagus nerve regulation. Such research cannot provide quantitative evidence for subsequent precise structural design.

In addition to direct signal transmission via the vagus nerve, SCFAs also provide structural protection for central homeostasis by stabilizing the blood-brain barrier. Engineered white and black fungus polysaccharides resistant to simulated gastrointestinal digestion, allowing them to reach the colon intact to exert their effects (Cai et al., 2023; Ma et al., 2026). These polysaccharides specifically promote acetate production and upregulate tight junction protein expression to stabilize the blood-brain barrier. This reduces the leakage of peripheral pro-inflammatory substances into the central nervous system at the source and builds a defensive barrier for normal physiological functions of central nerves. Excessively enhanced blood-brain barrier homeostasis is not always beneficial. Moderately increased barrier permeability facilitates immune cell infiltration and pathogen clearance during acute infection or trauma. Continuous barrier reinforcement mediated by SCFAs may hinder necessary neuroimmune communication (Fock and Parnova, 2023). Excessively enhanced blood-brain barrier homeostasis is not always beneficial. Multiple studies have directly demonstrated that under acute infection or traumatic conditions, moderate elevation of barrier permeability facilitates immune cell infiltration and pathogen clearance. Continuous barrier reinforcement mediated by SCFAs may hinder necessary neuroimmune communication. Furthermore, current studies have not clarified whether the protective effects of SCFAs on the blood-brain barrier act directly on endothelial cells or are indirectly achieved through systemic anti-inflammatory effects. If mediated indirectly, the targeted regulatory efficiency of engineered polysaccharides may be overestimated (Qiqi et al., 2024).

### 5.5 Interactions among neuroprotective pathways of engineered food-derived polysaccharides via the MGB axis

This section systematically elaborates four functional pathways responsible for the neuroprotective effects of engineered food-derived polysaccharides through the microbiota-gut-brain axis, including immune and inflammatory regulation, intestinal barrier restoration, neuroendocrine modulation, and direct gut-brain neural communication. These four pathways establish complex interactive relationships through shared molecular sites and feedback loops. Nrf2 serves as a major transcription factor governing antioxidant responses. The activation of Nrf2 can upregulate the expression of antioxidant enzymes such as glutathione S-transferase and heme oxygenase-1 to eliminate reactive oxygen species, while suppressing the transcription of pro-inflammatory factors driven by NF-κB. Such biological performance links oxidative stress responses with immune and inflammatory processes effectively.

Butyrate produced by short-chain fatty acid-synthesizing microbiota can epigenetically activate Nrf2 through the inhibition of histone deacetylase. Engineered polysaccharides enrich butyrate-producing microbes and thereby exert dual effects of antioxidant defense and anti-inflammation. This interactive mechanism explains the experimental phenomenon that many modified polysaccharides reduce microglial activation and alleviate neuronal lipid peroxidation damage simultaneously. GLP-1 secreted by intestinal L cells modulates peripheral glucose homeostasis and binds to corresponding receptors at vagal afferent terminals to trigger the gut-brain neural axis. Vagal efferent fibers regulate the secretory activity of intestinal L cells via cholinergic anti-inflammatory reflexes and form a bidirectional signal amplification loop. Engineered polysaccharides stabilize short-chain fatty acid-mediated GLP-1 secretion and generate synergistic effects with the vagal nerve activation pathway. The combined neuroprotective performance in the central nervous system can exceed the efficacy produced by any single pathway alone to a certain extent.

The restoration of the intestinal epithelial barrier acts as a prerequisite for the normal operation of all downstream pathways. Damage to the intestinal epithelial barrier allows endotoxins to enter the blood circulation and trigger low-grade systemic inflammation. The inflammatory state can reduce the sensitivity of GLP-1 receptors, impair vagal nerve signal transmission, and elevate oxidative stress levels in the central nervous system. Engineered food-derived polysaccharides restore intestinal tight junctions through the butyrate-HIF-1α axis and build a permissible microenvironment for the regular operation of immune, endocrine and neural pathways. Rescue experiments based on barrier-deficient animal models demonstrate that the simple restoration of intestinal barrier function can partially reproduce the neuroprotective effects of engineered polysaccharides, which indicates the upstream regulatory role of the intestinal epithelial barrier in the whole regulatory system.

Host genetic background and disease progression stages can change the intensity and direction of crosstalk among different pathways. Parkinson's patients carrying specific variants of free fatty acid receptor 3 show weakened neuroprotective responses mediated by SCFAs. The decreased vagal nerve sensitivity and GLP-1 resistance occurred in the advanced stage of neurodegenerative diseases may impair the function of partial regulatory pathways. The overall neuroprotective capacity of engineered food-derived polysaccharides originates from the synergistic crosstalk of multiple pathways. Systematic interpretation of the interactive relationships among these pathways will facilitate the development of next-generation precision prebiotics with multi-network regulatory properties.

## 6 Safety evaluation and clinical translation bottlenecks of engineered food-derived polysaccharides

**Fig. 8:**
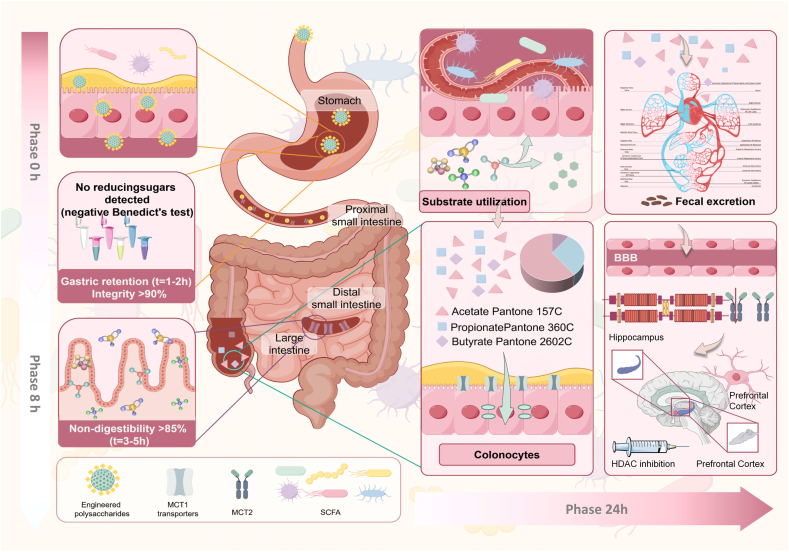
Schematic diagram of the in vivo fate and bioavailability of engineered polysaccharides This figure illustrates the in vivo kinetic profile and safety characteristics of engineered food-derived polysaccharides across 0 h, 8 h, and 24 h phases, including gastric retention, non-digestibility in the small intestine, substrate utilization to generate SCFAs, colonic absorption via MCT1/2 transporters, traversal of the blood-brain barrier (BBB) to exert HDAC inhibition in the brain, and fecal excretion, alongside safety indicators such as no reducing sugars detected.

Food safety and clinical translation feasibility of modified food-derived polysaccharides represent essential prerequisites for their application in precision nutritional interventions against neurological impairments (Fig. 8). Most current studies focus on structural modification and bioactive characterization of polysaccharides. Significant gaps remain in systematic safety assessment, regulatory compliance, and clinical translation pathways.

### 6.1 Current status of safety evaluation for engineered food-derived polysaccharides

Although engineered modification can significantly alter the physicochemical properties and biological activity of natural polysaccharides, it may simultaneously reshape their toxicological characteristics. Therefore, establishing a standardized and systematic safety evaluation system is not only a core prerequisite for the industrial application and clinical translation of engineered food-derived polysaccharides, but also the fundamental scientific basis for post-market regulation (Nam et al., 2024). Current mainstream international safety evaluation systems for engineered polysaccharides are primarily constructed around three core dimensions, acute toxicity, subchronic toxicity, and genotoxicity. However, existing research data exhibit significant shortcomings in terms of coverage depth, breadth, and the analysis of potential risk mechanisms, making it difficult to support systematic risk assessments of these substances under conditions of long-term dietary exposure or medicinal use. Regarding acute and subchronic toxicity, existing studies confirm that the oral median lethal dose (LD_50_) for most engineered polysaccharides exceeds 10 g/kg body weight, classifying them as practically non-toxic. Furthermore, 90-day subchronic feeding studies indicate that within the dosage range of 1-5 g/kg body weight per day did not result in histopathological damage or abnormal biochemical parameters in rodents. For example, the no-observed-adverse-effect level (NOAEL) of Tremella polysaccharide in male and female animals was as high as 7.21 g/kg and 8.18 g/kg, respectively. Although the NOAEL for acetylated ligustrum polysaccharide (FLL-PA12.5) is lower (250 mg/kg), its LD_50_ remains>2000 mg/kg. These results preliminarily support the short-term dietary safety of engineered polysaccharides as food ingredients (Ragul et al., 2025; Xu et al., 2026). However, it must be noted that over 90% of the existing toxicological data originate from short-term rodent studies, which can only reflect the potential risks of acute exposure. There is a severe lack of research on key safety endpoints such as chronic toxicity, carcinogenicity, and reproductive and developmental toxicity over≥12 months, which completely fails to meet the risk assessment requirements of global regulatory authorities for long-term dietary exposure or products intended for long-term intervention in chronic diseases (Mandil et al., 2016). Regarding genotoxicity assessment, current studies primarily rely on basic tests such as the bacterial reverse mutation assay (Ames test) and the mammalian erythrocyte micronucleus test. They do not follow the ICH S2 (R1) guideline to complete a full suite of genotoxicity battery tests, and generally overlook the potential mutagenic risk of engineered polysaccharide intestinal metabolites (Snodin, 2020). Furthermore, existing studies only perform qualitative screening for impurities in the final product and have not established quantitative limit standards for impurities correlated with dietary exposure levels, falling far short of the safety standards required for food additives or pharmaceutical excipients. In contrast, research findings regarding technical-grade products of compounds such as the pesticide carbendazim—which exhibit Ames-positive results due to impurities while the pure substance shows no mutagenicity—have fully highlighted the central role of impurity control in genotoxicity assessment (Snodin, 2020; El Ghoul et al., 2025). Furthermore, gastrointestinal reactions such as bloating and diarrhea caused by high-dose intake of engineered polysaccharides are often simplistically attributed to gas production from intestinal fermentation. There is a lack of mechanistic analysis regarding long-term damage to intestinal motility and the mucosal barrier, and no dose-response relationship to establish the Maximum Tolerated Dose (MTD) and Acceptable Daily Intake (ADI), failing to provide a scientific basis for setting safety thresholds for long-term human consumption (Li et al., 2024). Different chemical modification strategies can induce structure-dependent safety risks in engineered polysaccharides. Such risks are rarely systematically evaluated within existing assessment frameworks and are a core reason for the deviation of polysaccharide in vivo effects from expectations. Among these, sulfation—a mainstream modification method— introduces sulfate groups that release sulfate ions during colonic fermentation. This can drive the overproliferation of sulfate-reducing bacteria (SRB) and the metabolic production of hydrogen sulfide (H_2_S). Excessive H_2_S not only disrupts tight junctions in the intestinal epithelium and induces chronic intestinal inflammation but also directly inhibits the proliferation of SCFA-producing microbiota, ultimately completely offsetting the probiotic effects of engineered polysaccharides (Liu et al., 2025b). Furthermore, the structure of sulfated polysaccharides is highly similar to that of heparin; high-dose intake can prolong activated partial thromboplastin time (APTT) and interfere with the clinical efficacy of anticoagulant drugs, posing a clear risk of bleeding. Of even greater concern is that it may unintentionally regulate key cellular signaling pathways such as Wnt and FGF, thereby affecting normal cell proliferation and differentiation (Nunes and Coimbra, 2019). Furthermore, residues of heavy metals, organic solvents, and unreacted reagents associated with the chemical modification process exhibit clear dose-dependent toxicity. Although functionalized nano-cellulose materials can now be precisely tested for trace amounts of Hg^2+^ and Cr^3+^, current research has not established specific impurity detection lists or limit standards for engineered polysaccharides of different modification types. Consequently, it is impossible to guarantee that the final product meets the purity requirements of pharmacopoeias or food additive regulations. Enzymatic modification requires additional attention to the immunogenicity and sensitizing potential of exogenous enzyme proteins. Existing evidence indicates that fungal-derived glycosidases can induce allergic reactions in the intestinal mucosa, yet such critical evaluation steps are generally absent in current research (Sukhavattanakul et al., 2023; Friday and Edgar, 2026). Furthermore, as engineered polysaccharides serve as selective carbon sources for the gut microbiota, long-term high-dose intake may disrupt the nutritional balance of the gut microbiota. Studies have confirmed that high doses of carboxymethylated pectin can lead to abnormal proliferation of the Bacteroidetes phylum and an imbalance in the ratio of Firmicutes/Bacteroidetes ratio, and such long-term disruptions to the gut microbiome cannot be captured by short-term toxicological tests. The current evaluation system focuses solely on changes in the abundance of target SCFA-producing bacteria, failing to systematically assess the impact of engineered polysaccharide intake on overall gut microbiota diversity and the risk of opportunistic pathogen proliferation, revealing significant shortcomings in the monitoring system (Xie et al., 2021). More critically, the intestinal metabolic profiles of modified polysaccharides differ significantly from those of their natural precursors. Yet, existing research has almost exclusively focused on their regulatory effects on SCFA production, completely overlooking the potential toxicity of unintended metabolites. For instance, the metabolism of nitrogen-containing polysaccharides can generate ammonia, phenolic neurotoxic substances, inducing intestinal oxidative stress and DNA damage; meanwhile, excessive intake of branched-chain SCFAs is closely associated with insulin resistance and exacerbated neuroinflammation (Njoku et al., 2023). Simultaneously, the horizontal transfer of antimicrobial resistance genes (ARGs) has emerged as a new safety blind spot associated with long-term consumption of engineered polysaccharides. Prolonged intake of engineered polysaccharides may alter the selective pressure on the gut microbiota, driving the enrichment of opportunistic pathogens carrying ARGs and increasing the risk of horizontal transmission of resistance genes. This risk not only threatens individual gut homeostasis but also poses a public health concern, necessitating its urgent inclusion in the long-term safety evaluation framework for engineered polysaccharides. In summary, the current safety evaluation system for engineered polysaccharides has significant deficiencies in terms of coverage of chronic toxicity endpoints, the completeness of genotoxicity assessments, the identification of structure-specific risks, the establishment of impurity limit standards, the evaluation of long-term effects on the gut microbiome, and the analysis of the toxicity of unintended metabolites. To facilitate the transition of these materials from basic laboratory research to clinical translation and commercial application, it is essential to establish a new, systematic safety evaluation paradigm that integrates multidimensional endpoints, covers the entire lifecycle, and accommodates different types of modifications.

### 6.2 Dietary intake feasibility and human equivalent dose conversion of engineered food-derived polysaccharides

Dietary fiber intake guidelines for residents in major countries and regions provide a reference for establishing recommended daily intake levels of modified polysaccharides. Dietary Reference Intakes for Chinese Residents (2023 Edition) recommends an adequate intake of dietary fiber of 25 g per day for adults aged 18 to 49 years. The U.S. Food and Drug Administration and the Institute of Medicine of the United States recommend daily intake of 25 to 38 g for adults. The European Food Safety Authority suggests a minimum daily dietary fiber intake of no less than 25 g for adults in Europe.

In existing preclinical animal studies, the effective intervention doses of modified polysaccharides are mostly converted to a range of 5 to 20 g per day in human equivalent doses. This range is highly consistent with the mainstream dietary fiber intake recommendations worldwide. The values do not exceed the reasonable daily dietary fiber intake for residents and show favorable feasibility for dietary application (Table 4).

**Table 4:** Preclinical safety evaluation indicators and dietary feasibility assessment framework for engineered polysaccharides.

Evaluation Dimensions	Core Evaluation Contents and Parameters	Reference Standards and Basis
Key Parameters of Acute and Subchronic Toxicity	Acute toxicity: median lethal dose (LD_50_), maximum tolerated dose (MTD); subchronic toxicity: no-observed-adverse-effect level (NOAEL), lowest-observed-adverse-effect level (LOAEL), body weight changes, hematological/serum biochemical parameters, organ coefficients, and histopathological indicators derived from 90-day oral feeding studies	OECD Guidelines for the Testing of Chemicals; GB 15193 *Procedures for Toxicological Evaluation of Food Safety*; EFSA Guidelines on Risk Assessment of Food Additives
Genotoxicity Evaluation Endpoints	Bacterial reverse mutation test (Ames test), mammalian erythrocyte micronucleus test, mammalian cell chromosome aberration test, and in vitro mammalian cell gene mutation test	ICH S2(R1) Guideline on Genotoxicity Testing and Data Interpretation for Pharmaceuticals; *Procedures and Methods for Toxicological Evaluation of Food Safety* (China)
Corrected Human Equivalent Dose (HED) and Dietary Intake Recommendations	Corrected HED = value calculated by traditional formula×correction factor for microbiota fermentation efficiency × correction factor for colonic delivery efficiency×correction factor for dietary background; recommended daily intake for healthy adults: 5–20 g/d; intake design should be integrated with total daily dietary fiber intake of residents	FDA Guidance for Industry: Estimating the Maximum Safe Starting Dose in Initial Clinical Trials; EFSA Dietary Reference Values for Dietary Fiber; *Dietary Reference Intakes (DRIs) for Chinese Residents*
Long-Term Safety Evaluation Indicators	12-month chronic toxicity test, carcinogenicity test, reproductive and developmental toxicity test, assessment of impact on intestinal microecological homeostasis, and evaluation of unintended effects of metabolites	EFSA Guidance on Risk Assessment of Novel Foods; FDA Guidance for Industry: Safety Assessment of Food Additives

However, feasible dosing does not equate to reproducible efficacy. Similarly, a dose within the recommended range does not guarantee physiological tolerance. Gastrointestinal tolerance thresholds, metabolic response efficiency, and microbiota adaptability to modified polysaccharides should be assessed independently for each individual. Individuals differ substantially in gastrointestinal tolerance thresholds and metabolic response efficiency to modified polysaccharides. The dose range of 5 to 20 g per day only reflects the average population effect. This range cannot account for individual variations in tolerance caused by enterotype differentiation, disease subtypes, and concomitant medication. On the other hand, a complete system and relevant standards extending from in vitro fermentation to animal models and finally to human applications have not been established. Precise dose setting based on the response threshold of target strains remains difficult to achieve. This discrepancy leads to insufficient response rates in human clinical trials when using effective doses identified in animal experiments.

### 6.3 Core bottlenecks and existing challenges in clinical translation

The translation of modified food-derived polysaccharides from basic laboratory research to industrial application and clinical practice still faces substantial challenges. A primary limiting factor is the systematic lack of clinical evidence. Most existing studies are limited to cell and animal experiments (Yan et al., 2026). The few completed human trials are characterized by small sample sizes and an absence of high-quality randomized controlled trial designs. These trials primarily rely on surrogate endpoints and lack supporting data from hard clinical outcomes (Bedarf et al., 2025). Meanwhile, current studies have not systematically verified the functional stability of modified polysaccharides in the human intestinal tract. The differences in fermentation efficiency across intestinal segments have not been distinguished. Long-term follow-up data on the rebound risk of gut microbiota after treatment cessation are also lacking. These gaps currently limit the provision of robust evidence-based support for long-term clinical use in populations with neurological disorders. Individual heterogeneity in host gut microbiota strongly influences the reproducibility of intervention effects. This heterogeneity is shaped not only by baseline microbiota characteristics but also by host genetic background and long-term dietary habits. Enterotype-specific responses represent the dominant factor underlying individual variations in efficacy. The fermentation capacity of *Bacteroides*-dominated enterotypes and *Prevotella*-dominated enterotypes toward the same modified polysaccharide differs significantly (Bartsch et al., 2025).

SCFA production from highly branched pectin and sulfated polysaccharides is 20% to 40% higher in individuals with *Bacteroides*-dominated enterotypes than in those with *Prevotella*-dominated enterotypes. Linear polysaccharides such as arabinogalactan generate propionate with significantly higher efficiency in *Prevotella*-dominated enterotypes. Immature industrial production and quality control systems may restrict commercialization. Standardization of raw materials, structural uniformity, and batch-to-batch consistency form the foundation of an industrial system for modified polysaccharides. Most existing preparation techniques remain at the laboratory scale (Ferreira Filho R dos et al., 2025). Combined modification processes that integrate enzymatic directional degradation and chemical modification face notable technical barriers to scaled production. Reaction conditions precisely controlled in small-scale trials are difficult to maintain with uniform stability after scale-up. The high cost of specialized enzyme preparations further limits large-scale application.

### 6.4 Solutions and future development roadmap for precision prebiotics

Precision prebiotics offer systematic strategies to address host heterogeneity, uncontrolled safety risks, and unclear regulatory pathways through precise structural modification, targeted microbiota regulation, and establishment of a full-chain evaluation system (Ali et al., 2025). These compounds may serve as a candidate platform for precision nutritional intervention in neurological disorders. In terms of the efficacy evaluation system, precision prebiotics has the potential to enhance individual responsiveness by linking structural targeting to strain specificity. This framework addresses the limitation of conventional population-based data that masks individual response rates. In response to enterotype-specific variations in response, precision prebiotics use directional modification of polysaccharide structures to match the carbohydrate-active enzyme (CAZyme) substrate preferences of functional bacteria in hosts with different enterotypes (Bartsch et al., 2025). These modifications enable precise enrichment of target strains at the species level, aiming for greater specificity compared to non-specific modulation. Efficacy evaluation incorporates three preset indicators: response thresholds of target strains, enterotype-stratified subgroups, and hard endpoints for neurological phenotypes (Takahashi et al., 2025). Baseline enterotype, microbiota characteristics, metabolic phenotype, and efficacy endpoints are evaluated collectively. This approach replaces the traditional population-averaged evaluation model and resolves efficacy fluctuations caused by host heterogeneity at the trial design stage. Clinical studies of Synergy1 inulin and specific fructooligosaccharides have confirmed that stratified evaluation based on target strain response thresholds can increase intervention response rates from 40% to over 75%. This provides a reference for efficacy evaluation of modified food-derived polysaccharides. Combined with a preclinical dose conversion system, precise translation of effective animal doses to human equivalent doses further ensures the scientific validity and reproducibility of human intervention doses (Li et al., 2026). This strategy helps resolve dose heterogeneity across in vitro, animal, and human systems. The framework shows potential to shift from generalized intervention to precision responsiveness by addressing contradictions including individual microbiota heterogeneity, enterotype-driven efficacy fluctuations, weak targeting of native polysaccharides, and population-based masking of individual differences.

Safety evaluation and regulatory compliance for modified food-derived polysaccharides. This framework fills gaps in existing safety evidence and the shortage of long-term toxicological data. Structure-specific safety risks are managed by precise regulation of polysaccharide modification sites, branching degree, and substitution degree. These modifications retain target strain enrichment activity while avoiding non-specific effects and bleeding risks associated with sulfation, carboxymethylation, and other modifications. Safety variations caused by individual metabolic heterogeneity are addressed through batch traceability, colonic targeted delivery design, and real-time monitoring of intestinal metabolism. The evaluation system includes toxicity of non-target metabolites, risks of antibiotic resistance gene transfer, and long-term disturbance of intestinal microecology. A full life-cycle safety evaluation model suitable for long-term intervention in neurological disorders has been proposed. This model is designed to meet key risk assessment considerations of global regulatory agencies for products used in long-term chronic disease intervention. Regulatory compliance pathways. In terms of regulatory compliance, precision prebiotics help overcome barriers posed by unclear regulatory pathways. Although the European Food Safety Authority (EFSA) and the U.S. Food and Drug Administration (FDA) have not approved health claims for neurotargeted prebiotics, both agencies have acknowledged the relevance of regulatory frameworks linking microbiota modulation, metabolite changes, and host physiological outcomes. Precision prebiotics achieve strain-level precise regulation through structural modification, which aligns with review requirements of regulatory agencies. A progressive approach is recommended for modified food-derived polysaccharides. Compliance filings for conventional foods and dietary supplements are completed first based on the status of dietary fiber as a food ingredient. High-quality, multi-center, large-sample randomized controlled trials are then used to support claims related to reduced risk of specific diseases. This progressive strategy may eventually facilitate the clinical translation of prescription-level live biotherapeutic products. The approach provides a theoretical basis for regulatory filings of neurotargeted modified polysaccharides. Tiered and phased filing pathways can be developed according to regulatory frameworks in three major regions to resolve unclear regulatory routes. For the Chinese market, physical modified polysaccharides are launched as conventional foods based on dietary fiber ingredients. Simultaneously, filings for novel food ingredients are initiated for chemically or combinatorially modified polysaccharides. Clinical data from Chinese populations may be accumulated using gut microbiota regulation and intestinal barrier improvement as functional claims. For the European market, modified polysaccharides are filed as novel foods, with emphasis on intestinal microbiome safety and full metabolic pathway data. Supporting studies for functional claims are conducted in line with EFSA guidance on microbiota-host co-metabolism. For the U.S. market, physical modified polysaccharides may be eligible for GRAS notification. Filings for food additives are advanced in parallel for chemically modified polysaccharides. Dietary supplement markets are expanded through structure-function claims in compliance with relevant regulations. The recognition of microbiota-driven metabolic regulation by EFSA and FDA creates a favorable regulatory environment. Strain-level precision regulation enabled by precision prebiotics meets review standards across three major regulatory systems. This framework offers a feasible strategy for phased, cross-region compliance and clinical translation. It addresses the lack of clear regulatory pathways, ambiguous clinical translation directions, and cross-border compliance barriers for neurotargeted prebiotics.

An AI-enabled personalized intervention roadmap follows a progressive development model. This roadmap aims to upgrade precision prebiotics from general supplements to prescription-level precision nutritional agents. The initial stage focuses on construction of a response prediction model. Researchers collect time-series data of metagenomics, metabolomics, and neurological phenotypes from multi-center clinical cohorts (Quinn-Bohmann et al., 2026). These data are used to train an interpretable artificial intelligence model. The model identifies key strains and metabolic pathways that drive intervention responses and enables precise prediction of high-responder populations. The model requires a prediction accuracy corresponding to an R^2^ value greater than 0.8. Development of the response prediction model provides a solid data foundation for optimized personalized intervention plans. Researchers establish a simulation system for microbiome-host interactions using a digital twin platform (Bautista and López-Cortés, 2026). This system automatically matches the most suitable polysaccharide structure, intervention dose, and treatment duration based on host enterotype and disease stage. The intervention plan is tailored to individual physiological conditions and resolves uneven responses to universal protocols. Long-term implementation of personalized intervention requires full-cycle dynamic regulation. A full-cycle clinical decision support system is established for this purpose. The system integrates real-time data from wearable devices and home testing to enable dynamic monitoring of intervention effects, real-time dose adjustment, and early warning of safety risks (Zhu and Li, 2025). A closed-loop personalized intervention system is formed with seamless links among screening, optimization, and follow-up. AI-enabled analysis addresses intervention heterogeneity caused by host genetics, diet, and baseline microbiota (Barrera-Suarez et al., 2026). This strategy supports truly personalized intervention and may resolve the ineffectiveness of universal protocols. Full-process standardized management systems must be established to support clinical translation and industrialization of precision prebiotics. These systems provide actionable protocols for complete translation from basic research to clinical application. Raw material standardization represents the foundational prerequisite for industrialization. Full-chain raw material control specifications are established from source to finished product. Uniform standards are defined for plant source, origin traceability, harvesting, and pretreatment of polysaccharide raw materials (Liu et al., 2026c). Mandatory limits for raw material purity and pollutant residues are set to provide uniformly stable starting materials for subsequent engineered modification. Uniform minimum standards for structural characterization are established. Parameters including molecular weight distribution, monosaccharide composition, glycosidic bond configuration, and functional group substitution degree are clearly defined. Standardized detection techniques common to the industry are adopted to ensure comparable and repeatable characterization data across batches and laboratories. Stable control of batch-to-batch consistency is achieved through optimized large-scale production processes and full-process quality traceability systems. Acceptable fluctuation ranges for process parameters are defined. The relative standard deviation of structure, activity, and functional potential of final products is controlled within 10%. A three-tier progressive bridging strategy is established across in vitro fermentation, animal models, and human trials. The strategy uses primary screening via in vitro fermentation of human fecal microbiota, in vivo validation using humanized microbiota mouse models, and small-scale human exploratory trials (Lai et al., 2025b). This approach resolves inconsistencies between in vitro experiments and human clinical trial results. A multi-dimensional, quantifiable biomarker panel is constructed. A response prediction model is established using indicators including baseline enterotype and target strain abundance (Kolodziejczyk et al., 2019). A full-chain efficacy evaluation system is developed from microbial metabolism to neurological functional endpoints. Disease indications are prioritized based on clinical urgency, intervention windows, and clarity of pathological mechanisms. Indications with the highest intervention value, such as the preclinical stage of Alzheimer's disease and inflammatory subtypes of major depressive disorder, are prioritized (Fares, 2026). Applications are gradually expanded to other disease settings. This full-process management system deeply integrates efficacy evaluation, safety control, and regulatory compliance pathways. It provides a robust operational foundation for industrialization of precision prebiotics. In summary, the clinical translation of modified food-derived polysaccharides as neurotargeted precision prebiotics faces dilemmas in efficacy evaluation, safety control, regulatory adaptation, and personalized intervention. Progressive efficacy evaluation, full life-cycle safety assessment, region-adapted regulatory filings, and AI-enabled personalized intervention systems resolve issues including host microbiota heterogeneity, inherent defects of native polysaccharides, insufficient safety evidence, ambiguous clinical translation pathways, and individual response fluctuations. These strategies collectively aim to facilitate the complete translation from basic research to clinical implementation.

## 7 Discussion

This review presents a comprehensive mechanistic framework. First, subtype characteristics of various neurological disorders are systematically classified. Polysaccharide molecular structures are then adjusted based on disease-specific features. Subsequent selective enrichment of functional intestinal bacterial strains regulates the types and proportions of short-chain fatty acid (SCFA) production. These processes ultimately act on neural-related pathways to achieve targeted modulation. This research framework addresses two critical limitations in the current field. First, most existing review articles discuss neuropathological mechanisms and polysaccharide modification technologies in isolation. No systematic mechanistic correlations have been established between these two areas (Andoh et al., 2003). Second, current prebiotic research remains largely at the level of correlational analyses among native polysaccharides, gut microbiota characteristics, and neurophysiological effects. No theoretical frameworks guiding the precise design and development of polysaccharides have been formulated. Practical research paradigms for real-world application are also lacking (Chen et al., 2026a). In neurological disorders, the pathological mechanisms of neural injury mediated by dysbiosis of SCFA-producing microbiota do not involve non-specific reductions in microbial abundance. Instead, they manifest as disease-specific cascade disruptions in signaling pathways. Each type of pathological damage contains molecular nodes that can be precisely targeted by engineered food-derived polysaccharides. This discovery fundamentally transcends the limitations of existing studies that focus exclusively on microbiota-phenotype correlations (Gupta and Kumar, 2026).

Specifically, the pathological targets in the preclinical MCI stage of AD are aberrant BACE1 transcription and impaired microglial Aβ phagocytosis. The corresponding intervention strategy involves β-glucan with site-specific sulfation at the C-6 position. This modified polysaccharide matches the substrate recognition preferences of PUL systems in *Bacteroides* and *Lachnospiraceae* genera. It achieves strain-level precise enrichment and sustained stable butyrate production (Cantu-Jungles et al., 2024). The intervention target in the prodromal stage of PD is α-synuclein misfolding in the enteric nervous system and its retrograde gut-brain transmission. Acetylated highly branched pectin with a degree of substitution of 0.3 to 0.5 can be used. This polysaccharide targets the CAZyme substrate characteristics of *Ruminococcaceae* and *Faecalibacterium*. It repairs the intestinal barrier and blocks the initiation and propagation of α-synuclein pathology (Deisenhofer et al., 2024). The pathological feature of the inflammatory subtype of MDD is tryptophan metabolism diversion mediated by excessive IDO pathway activation. Carboxymethylated pectin with a degree of substitution of 0.6 to 0.8 can directionally enrich *Faecalibacterium* and *Roseburia* to elevate colonic butyrate levels. This inhibits the cascade amplification of peripheral inflammation to the central nervous system at the source (Kim et al., 2023). The intervention targets in the acute phase of post-stroke cognitive impairment (PSCI) are neuroinflammatory cascades and acute blood-brain barrier damage. Low-molecular-weight enzymatically hydrolyzed oligosaccharides are suitable for this scenario due to their rapid fermentation properties. These oligosaccharides achieve rapid SCFA synthesis and acute phase inflammation blockade by targeting *Bacteroides* and *Bifidobacterium* genera (Tang et al., 2024). A key limitation of current research is that only phenotypic intervention effects of engineered polysaccharides have been verified. No complete causal chain linking disease pathological targets, polysaccharide fine structures, strain functional activation, and neurological phenotype improvement has been established. This gap represents a major reason for the frequent negative outcomes in clinical studies of native prebiotics.

Despite these research advances, engineered food-derived polysaccharides still face significant challenges in their transition to precision nutritional interventions and clinical translation. The high interindividual variability of the human microbiome represents a major barrier to clinical translation of precision prebiotics. This variability is not limited to single-dimensional differences in microbial abundance. It constitutes a multi-dimensional, hierarchical systemic heterogeneity. Host enterotype differentiation leads to fundamental differences in substrate utilization capacity. Fermentation efficiency of the same polysaccharide can differ by 20% to 40% between individuals with *Bacteroides*-dominated and *Prevotella*-dominated enterotypes (Tang et al., 2024). Most existing studies analyze data based on population-averaged effects. Interventional response heterogeneity caused by enterotype differentiation is largely ignored (Tang et al., 2024). Differences in disease subtypes further result in distinct deficiency profiles of functional microbiota. The inflammatory and non-inflammatory subtypes of MDD exhibit completely different dysbiosis characteristics of SCFA-producing microbiota and intervention targets. Universal polysaccharides cannot simultaneously match the substrate preferences and metabolic features of both subtypes (Ślinko et al., 2026). Confounding factors including host genetic background, long-term dietary patterns, and concomitant medications cause significant individual differences in CAZyme-encoding gene abundance and PUL system function in the gut microbiota of patients with the same disease and enterotype. These differences directly lead to substantial fluctuations in SCFA production efficiency and clinical response to the same polysaccharide.

No validated microbiome or metabolome biomarkers for stable prediction of interventional response have been identified in the field. Accurate stratification of high-responder populations prior to intervention remains unachievable. This deficiency further amplifies the interference of individual heterogeneity on clinical study results. Furthermore, clear distinction between correlation and causality among variables remains challenging. Most studies infer the neuroprotective mechanisms of polysaccharides indirectly based on correlations between fluctuations in microbial abundance, changes in SCFA levels, and improvements in neurological phenotypes. Rigorous causal validation using experimental approaches such as germ-free animals, gene knockout animals, and microbiota-specific depletion models is lacking (Bertin et al., 2025). Research on in vivo transport and metabolic processes and long-term safety of engineered polysaccharides is severely lagging. Comprehensive toxicological data are insufficient to support critical safety assessments. These assessments include risks of in vivo accumulation after long-term oral administration, potential biological toxicity, and long-term impacts on intestinal microecological homeostasis. This gap prevents compliance with regulatory requirements for food ingredient application and clinical translation. In addition, industrial production and full-process quality control systems for engineered polysaccharides are not fully established. Low-cost, scalable modification and purification technologies are scarce. Unified quality evaluation standards and industry specifications are lacking. These deficiencies severely restrict industrialization and market promotion.

**Fig. 9:**
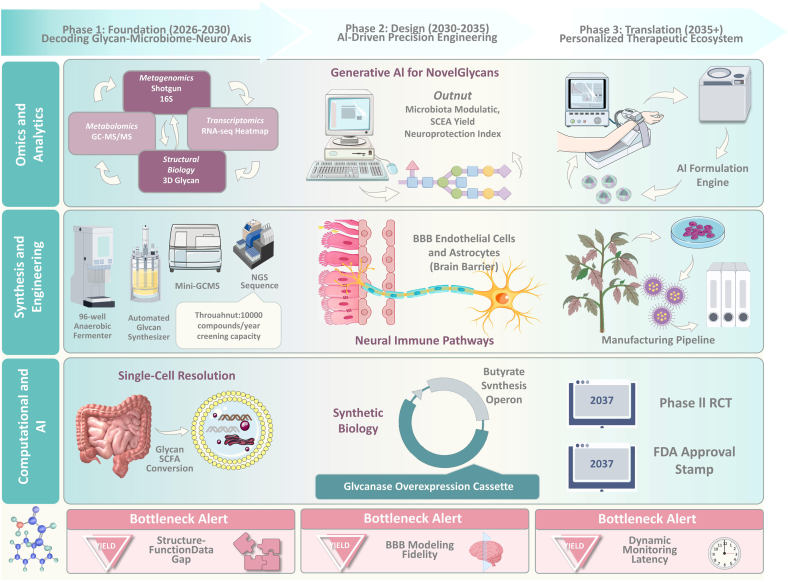
Technology roadmap for the future research and development of precision prebioticsThis figure outlines the three-phase roadmap for advancing engineered polysaccharides in neuroprotection via the gut-brain axis, integrating omics analytics, synthesis engineering, and computational AI technologies to decode the glycan-microbiome-neuro axis, enable AI-driven precision design, and build a personalized therapeutic ecosystem, while highlighting key bottlenecks in yield, BBB modeling, and dynamic monitoring.

Future research should address these challenges through a fundamental paradigm shift from empirical efficacy screening to mechanism-driven precision design, rather than incremental optimization of existing technologies. At the basic research level, the conventional research paradigm that prioritizes phenotypic correlations over causal mechanisms must be abandoned. Systematic studies should be conducted on the relationships among polysaccharide fine structures, strain functional activation, and neural pathway regulation. Synthetic biology-based single-strain functional validation systems, gene-edited animal models, and target-specific pathway blockade techniques should be employed. These approaches will clarify the microbial recognition targets, metabolite effects, and central regulatory molecular mechanisms corresponding to various structural modifications. The primary drivers of individual heterogeneity will also be identified, providing a foundation for precision prebiotic design (Shajahan et al., 2026). In the technical system, polysaccharide engineering technologies must transition from trial-and-error modification to predictive reverse design. Multi-omics big data and artificial intelligence technologies should be leveraged to construct quantitative predictive models. These models will link polysaccharide structural parameters, microbial CAZyme recognition characteristics, and metabolite output. This will enable directional design of polysaccharide structures based on disease pathological target requirements. Existing inefficient screening models will be supplemented and optimized, driving a systematic transformation of the field from discovery-based to design-based research (Rusman et al., 2025). At the top-level design of clinical translation, a clinical research model integrating disease risk stratification, baseline microbiota stratification, and intervention scheme matching should be established. Early intervention windows and pathologically defined disease subtypes should be prioritized. High-quality evidence-based medical evidence should be accumulated through multi-center, large-sample randomized controlled trials. Research norms and evaluation standards aligned with global regulatory frameworks should be established. These efforts will promote the translation of precision nutritional interventions from scientific concepts to accessible and widely implementable clinical tools (Yang et al., 2024a). Furthermore, deep interdisciplinary integration of food science, microbiology, neuroscience, clinical medicine, and regulatory science must be advanced. This will address the current issues of fragmented research efforts and inconsistent standards across disciplines, laying a foundation for standardized and high-quality development of the entire field (Fig. 9).

In summary, engineered food-derived polysaccharides have emerged as a research hotspot in the interdisciplinary field of the microbiota-gut-brain axis and neuroscience. Their advantages include excellent biocompatibility, precise microbiota-targeted regulatory capacity, and multi-target neuroprotective properties. These polysaccharides provide a safe and widely accessible precision nutritional intervention approach for incurable neurological disorders such as AD and PD. They also promote deep interdisciplinary integration of food science, microbiology, and neuroscience, opening new directions for high-quality development of the functional food industry. Despite current challenges including incomplete mechanistic elucidation, significant interventional heterogeneity, and delayed clinical translation, continuous advancement of cutting-edge technologies and deepening basic research will enable engineered food-derived polysaccharides to play important roles in the prevention and treatment of central nervous system disorders and the maintenance of human brain health.

## 8 Conclusions

This study systematically elucidates the core mechanisms and application potential of engineered food-derived polysaccharides as next-generation precision prebiotics in the targeted regulation of intestinal SCFA-producing microbiota and exerting neuroprotective effects via the MGB axis, with functional characteristics significantly distinct from conventional broad-spectrum prebiotics. The results demonstrate that directional modulation of the fine molecular structures of polysaccharides through physical, chemical, biological, and combined modification technologies enables strain-level specific enrichment of target SCFA-producing microbiota, increasing the synthesis efficiency of key neuroprotective SCFAs such as butyrate by 20% to 60% while significantly reducing the risk of non-specific microbiota disturbance. These findings support the structure-activity relationship hypothesis of “polysaccharide fine structure-microbiota targeting-neuroprotective effect”, and reveal that the substrate preferences of CAZymes and the recognition specificity of the PULs system constitute the core molecular basis determining strain targeting. This precise regulatory mode simultaneously addresses the bottleneck problems of conventional prebiotics, including high interindividual response heterogeneity, uncontrollable SCFA metabolism, and low clinical translation efficiency, and exhibits unique application advantages particularly in critical intervention windows such as the MCI stage of AD and the prodromal stage of PD. Notably, the direct correlation between the chain length distribution of oligosaccharide fragments and neuroprotective effects has not been clearly verified, and a single structural modification strategy is difficult to simultaneously meet the intervention requirements of all neurological disorders. However, the construction of an AI-enabled personalized intervention roadmap and a full-process standardized management system provides a feasible approach for overcoming the clinical translation barriers caused by baseline heterogeneity of the host gut microbiota, and lays a foundation for the design of precise nutritional intervention regimens for different disease subtypes. Engineered food-derived polysaccharides with combined targeting specificity, safety, and scalable production potential are expected to become a novel precision nutritional intervention tool for the prevention and treatment of nerve injury-related diseases, providing new solutions for addressing the increasingly severe global public health challenge of central nervous system disorders.

## Author contributions

Yanmei Yin: Data curation; Formal analysis, Visualization, Writing-original draft; Yugang Li: Data curation, Formal analysis, Methodology, Writing-original draft; Xiaobin Zhang: Data curation, Formal analysis, Visualization, Writing-original draft; Mengwei Jia: Conceptualization, Supervision, Funding acquisition, Writing-review and editing; Shu Zhu: Conceptualization; Supervision; Resources; Writing-review and editing; Chengcheng Liu: Conceptualization, Project administration, Methodology, Writing-review and editing.

## Declaration of competing interest

The authors declare that they have no known competing financial interests or personal relationships that could have appeared to influence the work reported in this paper. All authors have approved the final version of this declaration and confirm there are no competing interests to disclose.
